# TRIM8-dependent K63-ubiquitinated PGK1 promotes glycolysis and angiogenesis in gastric cancer via interaction with ACAT1

**DOI:** 10.1038/s41419-025-08015-y

**Published:** 2025-11-03

**Authors:** Anqi Feng, Jianbin Zhang, Zeyu Wang, Zhukai Chen, Kang Fang, Zhaoxing Li, Hanyu Jiang, Zhuyun Leng, Shihan Zhang, Yuan Chu, Jingjing Lian, Tao Chen, Lechi Ye, Meidong Xu, Lingnan He

**Affiliations:** 1 Endoscopy Center, Department of Gastroenterology, Shanghai East Hospital, School of Medicine, Tongji University, No.150, Jimo Road, Pudong New Area, Shanghai, China; 2 School of Medicine, Tongji University, Address: No.500, Zhennan Road, Putuo District, Shanghai, China; 3 Advanced Research Institute, Tongji University, Address: No.1239, Siping Road, Yangpu District, Shanghai, China; 4 Department of Colorectal Surgery, Zhongshan Hospital, Fudan University, Address: No.180, Fenglin Road, Xuhui District, Shanghai, China

**Keywords:** Oncogenesis, Tumour angiogenesis, Gastric cancer, Cancer metabolism

## Abstract

Glycolysis is crucial for promoting cancer progression. However, the precise mechanism underlying glycolysis regulating the angiogenic process remains to be defined. Here, we demonstrate that in human gastric cancer cells, the E3 ligase TRIM8 promotes the K63-linked ubiquitination of the glycolytic enzyme PGK1 and improves its stability, which leads to acetyltransferase ACAT1 recruitment, increased interaction of PGK1 with ACAT1, and subsequent PGK1 acetylation-dependent glycolytic activity. This activity facilitates PGK1-mediated glycolysis, lactate accumulation and triggers a significant increase in endothelial cell migration and tube formation, which ultimately accelerates tumor angiogenesis in gastric cancer. TRIM8 levels are positively correlated with tumor angiogenesis and poor prognosis in gastric cancer patients. These findings elucidate a novel mechanism underlying the upregulation of angiogenesis mediated by K63 ubiquitination-regulated glycolysis in tumor cells and provide a molecular basis for eliminating gastric cancer angiogenesis by targeting TRIM8-dependent PGK1 K63 ubiquitination.

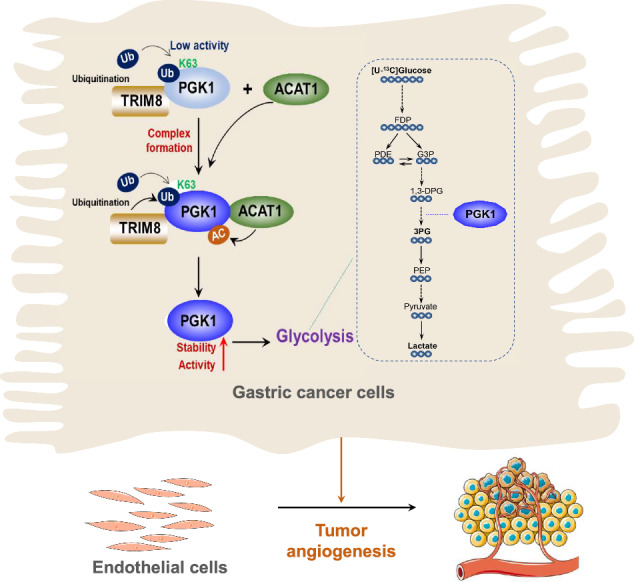

## Introduction

Gastric cancer (GC) is the fifth most common cancer and the third most common cause of cancer death globally [1]. Even with surgical resection and chemotherapy, the overall survival of GC patients is poor. This discouraging prognosis is largely attributed to late-stage diagnosis and limited treatment options [2]. Tumor growth is angiogenesis dependent [3], which is a hallmark of cancer. Anti-angiogenic therapy has a proven survival benefit in GC patients and has attracted increasing attention [4]. Nevertheless, limitations of anti-angiogenic therapies, which are mainly based on the inhibition of endothelial cell (EC) activation by angiogenic factors, especially VEGF, suggest that alternative antiangiogenic strategies might be considered [5]. Highly activated glycolysis with strongly increased lactate production regardless of oxygen availability in tumor cells, referred to as the Warburg effect, provides the metabolic requirements for tumor cell growth and proliferation [6]. Aerobic glycolysis also causes angiogenesis process through many pathways. Upregulation of glycolysis leads to microenvironmental acidosis, which results in angiogenesis [7]. Acidification of the tumor microenvironment by lactate and H^+^ promotes the secretion of angiogenic factors (e.g.,VEGF), thereby inducing tumor angiogenesis [8]. The production of pyruvate and lactate by glycolysis can induce the expression of HIF-1α and accelerate the angiogenesis process by mediating the transcription of angiogenic factors [9]. Metabolic reprogramming can control angiogenesis opens new horizons for treating this process under pathological conditions through metabolic approximation rather than targeting proangiogenic molecules [10]. Therefore, further investigation of the molecular mechanisms that underly the metabolic features that regulate the angiogenic process is crucial for the development of effective therapeutic strategies.

Recent studies have revealed that cancer cells are dependent on protein stability mechanisms, including protein folding and degradation [11]. Previous work has established that E3 ligases target PFKP, a key metabolic enzyme in glycolysis, for proteasomal degradation [12]. In cancer cells, suppressing E3 ligase function may result in persistent glycolysis [13]. The expression and degradation of core proangiogenic proteins are regulated by selective ubiquitin‒proteasome system [14]. Therefore, targeting the regulation of the ubiquitin–proteasome system is a promising strategy for the management of glycolysis that regulates the angiogenic process. Nevertheless, the regulatory metabolic functions that target the ubiquitin–proteasome system remain poorly understood, especially in cancers.

Tripartite motif-containing (TRIM) proteins have sequential tripartite structural motifs (RBCC): a RING finger domain, 1 or 2 B-box motifs, and a coiled-coil domain [15]. Members of the family of tripartite motif (TRIM)-containing proteins can be defined as E3 ubiquitin ligases, as they contain a RING-finger domain [16]. Accumulating evidences have further shown that TRIM family genes are involved in carcinogenesis and cancer progression [17]. TRIM family proteins are involved in a broad range of biological processes, such as cell differentiation, growth, proliferation, and apoptosis [18–20]. Recently, more attention has been given to the functions of TRIM8 in cancer progression: TRIM8 regulates critical cellular functions in cancers, including proliferation and migration, gene transcription, the cell cycle, and stemness [21–24]. However, the oncogenic function and underlying mechanism of TRIM8 in gastric cancer and glycolysis-mediated angiogenesis remain inconclusive.

In the present study, we investigate the role of TRIM8 in metabolism-promoted tumor angiogenesis and reveal a novel mechanism by which TRIM8 reprograms tumor cell glycolysis to promote tumor angiogenesis in gastric cancer.

## Materials and methods

### Cell lines and cell culture

The human gastric cancer cell lines SGC7901 were purchased from American Type Culture Collection (ATCC). BGC823 cell lines were purchased from Shanghai Zhong Qiao Xin Zhou Biotechnology Co., Ltd. All GC cell lines were identified by short tandem repeat analysis, and the results of mycoplasma test were negative. Cells were cultured with RPMI-1640 medium (Gibco, San Francisco, CA, USA) containing10% fetal bovine serum (Gibco) with 100 U/mL penicillin and streptomycin (Servicebio, Wuhan, China) at 37 °C in a humidified incubator of 5% CO2.

### HUVEC extraction and identification

Human umbilical vein endothelial cells (HUVECs) were obtained from human umbilical veins using Type I collagenase Hank’s solution as previously described [25]. HUVECs were cultured in Endothelial Cell Medium (ECM) (cat. No.1001, ScienceCell, San Diego, California, USA) containing 25 ml of fetal bovine serum (FBS, Cat. No. 0025), 5 ml of endothelial cell growth supplement (ECGS, Cat. No. 1052) and 5 ml of antibiotic solution (P/S, Cat. No. 0503). Cells were identified by immunofluorescence staining of anti- von Willebrand factor (VWF) (Bs-0586R, Bioss) and anti-PECAM1(ab9498, Abcam) for cell surface antigens.

### In vitro co-culture system

In vitro co-culture system between HUVECs and GC cells was established by using a Transwell with six-well plates in which HUVECs were indirectly co-cultured with GC cells and separated from GC cells by a semipermeable membrane (pore size of 0.6 μm). After 24 h of co-culture, HUVECs were collected for subsequent experiments such as migration assay and tube formation assay. Tumor cell-conditioned medium (CM) after culture mimics the components of the tumor microenvironment (TME) in vivo.

### Animal studies and clinical specimens

BALB/c nude mice (female, 4–6 weeks) were purchased from the Cavens Laboratory Animal Co. Ltd and housed under standard pathogen-free conditions in Experimental Animal Center of Shanghai East Hospital, Tongji University School of Medicine. The mice were randomly divided into groups of five mice each. 60 human samples come from the Endoscopy Center of Shanghai East Hospital. All samples were obtained with the patients’ informed consent, and the samples were processed histologically.

### Plasmid and lentivirus construction and transfection

The transfection procedure was performed as previously described [26]. The plasmids and lentiviruses targeting human TRIM8, PGK1, ACAT1 and non-targeting controls were designed and constructed from Hanbio Biotechnology Co.Ltd. TRIM8, PGK1, and ACAT1 overexpression plasmids and control plasmids were purchased from Hanbio Biotechnology Co.Ltd. Target cells were seeded and incubated overnight prior to infection. Medium was replaced with 1:2 diluted viral supernatant supplemented with 8 μg/mL polybrene, and incubated for 6–8 h, followed by replacement with growth medium. The control and shRNA cells were selected using puromycin (2 μg/mL) prior to the experiments.

### RNA extraction and real-time qPCR

Total RNA was extracted using Trizol (TAKARA, Japan). RNA was reverse transcribed using the Reverse Transcription system (TAKARA, Japan) and PCR was conducted with TB Green PCR Master Mix (TAKARA, Japan) using a 7500 Fast Real-Time PCR System (Applied Biosystems). Samples were normalized to glyceraldehyde-3-phosphate dehydrogenase (GAPDH). n = three independent experiments. Primers used in this study are listed in Supplementary Table 1.

### In vitro proliferation assay

CCK-8 cell proliferation assay -Cells were seeded in 96-well plates at 1000 cells/well. Cell viability was quantified every using CCK-8 Kit (Dojindo Laboratories, Kumamoto, Japan) at 450 nm, based on the manufacturer’s protocol. n= three independent experiments.

Colony formation assay- GC cells (1 × 10^4^cells) were plated into each well of a 12-well plate and cultured at 37 °C for 10 days, with adding fresh growth medium every 3 days. The colonies were fixed with 4% paraformaldehyde and stained with 1% crystal violet solution for 20 min. Then the colonies were counted. n= three independent experiments.

### In vitro HUVEC migration assay

Migration assays were performed using 24-well insert (membrane pore size, 3 mm; Corning, USA). 1000 HUVEC cells were resuspended in the serum-free ECM medium, transferred to the upper chamber, and incubated at 37 °C for 18 h. n = 3 wells per group. Complete ECM medium with 20% serum in the lower chamber was used as a chemoattractant. After 18 h, the medium was aspirated, and cells were removed from the inside surface of the upper chamber using cotton swabs. The cells were fixed with 4% paraformaldehyde for 30 min and stained with 1% crystal violet (Sigma-Aldrich, St. Louis, MO, USA) and photographed.

### Endothelial cell tube formation assay

The μ-Slide Angiogenesis plate (ibidi, Martin Reid, Germany) was coated with growth factor-reduced Matrigel (Corning, BD Biosciences, CA, USA) and polymerized in the incubator at 37 °C for 30 min. 1×104 HUVECs pre-treated with Calcein AM were resuspended and seeded into the μ-Slide Angiogenesis plate. n = 3 wells per group. After a 7 hr incubation of conditioned ECM medium with tumor supernatants, the tube network was photographed using a fluorescence microscope. The NIH Image J with the Angiogenesis plugin software was used on the tube network to detect total nodes/tubes /mesh in combination.

### Rat aortic ring assay

The rat aortic ring assay was performed as previously described with minor modifications [27]. The thoracic aorta was excised from a 9-week-old male Sprague–Dawley rat (Viton Lihua Biological Co., Ltd, Beijing, China). The thoracic aorta was cut into 1–2 mm long rings and rinsed with ice-cold phosphate-buffered saline (PBS) three times. n = 3 rings per group. The rings then were placed into 100 ul Matrigel-coated 48 well plates (Corning, BD) and incubated at 37 °C in 5% CO_2_ for 30–45 min. ECM (cat. No.1001, ScienceCell, San Diego, California, USA) containing 25 ml of fetal bovine serum (FBS, Cat. No. 0025), 5 ml of endothelial cell growth supplement (ECGS, Cat. No. 1052) and 5 ml of antibiotic solution (P/S, Cat. No. 0503) were added to the wells and incubated at 37 °C in 5% CO_2_ for 7 days. The rings were incubated with tumor-conditioned medium. At the end of incubation, the supernatant was discarded, and digital images of the aortic rings were generated. The number of microvessels emerging from the aortic rings was counted using the Image J software.

### In vivo tumorigenesis

Animals were housed under pathogen-free conditions in Experimental Animal Center of Shanghai East Hospital, Tongji University School of Medicine and were given autoclaved food and water and libitum. 2 × 10^6^ gastric cancer cells were injected subcutaneously into the right upper flanks of mice as previously described. n = 5 mice per group. Tumors were measured and tumor volume was calculated every two days by the formula (width^2^× length)/2. Tumors were dissected out and weighed at the end of the experiments.

### Immunohistochemistry (IHC) assay and immunofluorescence (IF) staining

Sections of tumors were processed as previously described. Sections were deparaffinized in xylene and rehydrated in a graded series of ethanol solutions, and antigen unmasked with 10 mM of sodium citrate (pH 6.0) for 3 cycles of 4 min each at 100 °C. After incubated with 3% H_2_O_2_ for 10 min to block endogenous peroxidase activity and blocked in 10% (v/v) goat serum in PBS for 1 h at room temperature, the sections were incubated overnight with antibodies against as follows: anti-TRIM8(Proteintech,27463-1-AP), anti-PECAM1(ab9498, Abcam) followed by one hour incubation at room temperature with the secondary antibody. Sections were washed in PBS, counterstained with hematoxylin, dehydrated and mounted. The whole tissue section was scored with staining intensity and percentage and the scoring scale was graded as follows: 0 points (no staining), 1 point (light brown staining), 2 points (brown staining) and 3 points (dark brown staining). The percentage of positive cells is divided into 4 levels: 1 point ( < 5%), 2 points (5–30%), 3 points (31–60%), 4 points (61–100%). The IHC staining score was calculated as follows: intensity score × percentage score. n = 20 samples per group.

### SDS–PAGE and western blotting analysis

Proteins were extracted in RIPA buffer containing phosphatase and protease inhibitor mixes (Beyotime Biotechnology, China) at 4 °C for 10–20 min. Proteins were resolved by SDS-PAGE and transferred onto nitrocellulose membrane. The membrane was blocked by 5% skimmed milk before immunoblotting with indicated antibodies followed by horseradish peroxidase (HRP)-conjugated secondary antibodies. Densitometry was performed using Millipore immobilon western chemilum HRP substrate and gray value was quantified with Image J. The primary antibodies used were anti-TRIM8 (Proteintech,27463-1-AP), PGK1 (Proteintech,17811-1-AP), anti-ACAT1 (Cell Signaling Technology, #44276), anti-ubiquitin (Santa Cruz, #sc-8017), anti-Acetylated-Lysine (Ac-K) (Cell Signaling Technology,#9441), anti-GAPDH (Cell Signaling Technology, #2118), anti-Tubulin (Cell Signaling Technology, #5568), anti-His-Tag (Santa Cruz, sc-8036), anti-HA-Tag (Santa Cruz, sc-7392), anti-DYKDDDDK (Flag) Tag (Cell Signaling Technology, #14793). n= three independent experiments.

### Mass spectrometry analysis

SCG7901 cells stably overexpressed with Flag-TRIM8 were lysed and immunoprecipitated with the flag antibody. n = 3 samples per group. The conjugated proteins were eluted by SDS-PAGE gels and observed with Pierce Silver Stain. Proteins in the gel were extracted again, and the extracts were dried in a SpeedVac vacuum centrifuge. The products were performed by liquid chromatography-tandem mass spectrometry (LC–MS/MS) (24600, Thermo) analysis.

### Immunoprecipitation (IP) assay

Immunoprecipitation was performed using protein A Immunoprecipitation (Roche) according to the manufacturer’s protocol. Cells were lysed (lysis buffer: 50 mM Tris, 150 mM NaCl, 2 mM EDTA, protease inhibitor cocktail (Complete Mini, Roche), 0.5% Triton X-100) and incubated 1 hr on ice. Cell lysates containing protein was incubated with the indicated antibody-conjugated beads at 4 °C overnight. Proteins conjugated to the beads were eluted and subjected to western blotting assays. n= three independent experiments.

### Measurement of glucose consumption and lactate production

Glucose consumption and lactate production was performed as previously described [28]. In brief, cells were seeded, and the medium was changed 6 h later with non-serum DMEM. Cells were then incubated for another 20 h. The culture medium was collected to measure the glucose and lactate concentrations. The glucose level was determined using a glucose assay kit (Sigma). The lactate level was determined using a lactate assay kit (E-BC-K044-M, Elabscience). Glucose consumption and lactate production were normalized to cell numbers.

n= three independent experiments.

### Glycolytic rate assay

Cells were plated at a density of 5 × 10^4^/well in an XF24 plate for 24 h. The media were exchanged for XF24 media (Agilent Technologies, Sana Clara, USA) 1 h before the assay. Glucose, oligomycin and 2-DG were diluted into XF24 media and loaded into the accompanying cartridge to achieve final concentrations of 10 mM, 1 mM and 100 mM respectively. Injections of the drugs occurred at the time points specified. The extracellular acidification rate (ECAR) was monitored using a Seahorse Bioscience XF24 Excellular Flux Anaylser. When ACAT1-mediated acetylation was involved, cells were pretreated with or without 0.5μM K-604 dihydrochloride (MCE, #217094-32-1, China), a potent and selective ACAT1 enzyme activity inhibitor. n= three independent experiments.

### Statistical analysis

All experiments were repeated at least three times, and the results were expressed as means ± SD. All statistical analyses were performed using GraphPad Prism software. Student’s t-test or the one-way analysis of variance (ANOVA) were used to analyze the data, and chi-square test was used to analyze differences in other variables, as appropriate. P value of ≤0.05 was considered statistically significant for all datasets. (*p < 0.05, **p < 0.01, ***p < 0.001).

## Results

### The expression of TRIM8 is correlated with the progression and angiogenesis of GC

**Fig. 1:**
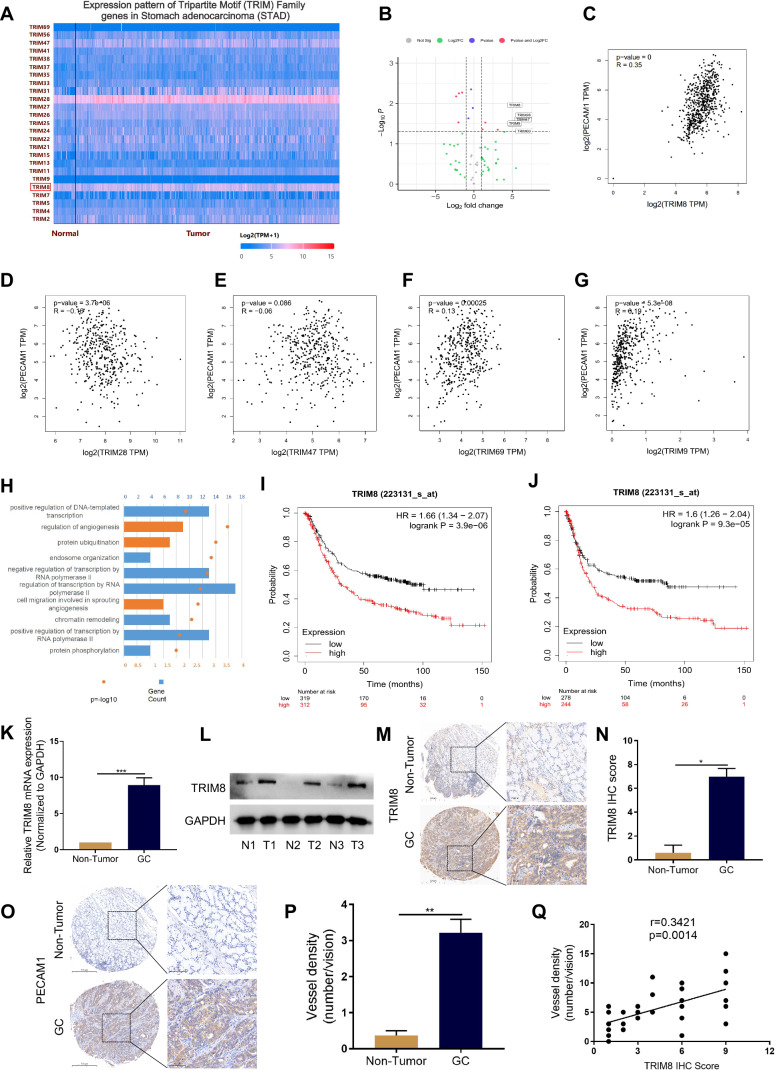
Overexpression of TRIM8 is correlated with vessel density in human GC tissues and predicts a poor prognosis. **A**, **B** The expression of genes encoding E3 ubiquitin ligase in the TRIM family in TCGA dataset. **C**–**G** Correlation analysis of TRIM8, TRIM9, TRIM28, TRIM47, TRIM69 and vessel density in GC. **H** Overexpression of TRIM8 mRNA in GC tissues than normal tissues in TCGA dataset. **I** The overall survival analysis was plotted using Kaplan–Meier Plotter for patients with GC. **J** The disease-free survival analysis was plotted using Kaplan–Meier Plotter for patients with GC. **K** TRIM8 expression level in GC tissues and adjacent non-tumor tissues detected by qRT-PCR. ***, p < 0.001. **L** TRIM8 expression level in GC tissues and adjacent non-tumor tissues was detected by western blot. **M** Representative IHC staining for TRIM8 in GC and adjacent nontumor tissues. **N** Quantification of M. *, p < 0.5. **O** Representative IHC staining for PECAM1 in GC and adjacent nontumor tissues. **P** Quantification of O. **, p < 0.01. **Q** Correlation analysis of TRIM8 and vessel density in GC.

To determine the potential role of the TRIM family in GC, we first examined the expression levels of the TRIM family in human GC tissues and adjacent nontumor tissues using the TCGA database. We found that the levels of 5 TRIM family members were significantly greater in GC tumors than in peritumor tissues (Fig. 1A, B, Fig. S1A). Notably, further bioinformatic analyses of TRIM family members and PECAM1 expression in GC through the GEPIA database revealed that TRIM8 expression was positively correlated with the vessel density of GC tissues (Fig. 1C–G). Enrichment (Gene Ontology; GO) analysis of TRIM8-related genes in the TCGA database revealed enrichment in biological processes relevant to angiogenesis (Fig. 1H). Kaplan–Meier Plotter analysis revealed that TRIM8 expression was negatively correlated with the survival and progression-free survival of GC patients in the TCGA database (Fig. 1I‒J). To validate the correlation between TRIM8 and angiogenesis in GC tissues, we determined the expression patterns of TRIM8 and PECAM1 in our cohort of GC patients. Western blot and immunohistochemistry (IHC) staining revealed greater expression of TRIM8 in tumors than in peritumor tissues (Fig. 1K–N). Consistently, significantly higher expression of PECAM1 was also detected in tumors than in peritumor tissues (Fig. 1O‒P). Moreover, further analysis revealed that TRIM8 expression was positively related to the vessel density of GC tissues (Fig. S1B, Fig. 1Q). Overall, these data indicate that the upregulation of TRIM8 expression is positively correlated with angiogenesis in human GC and predicts poor prognosis in GC patients.

### TRIM8 promotes angiogenesis in GC

**Fig. 2:**
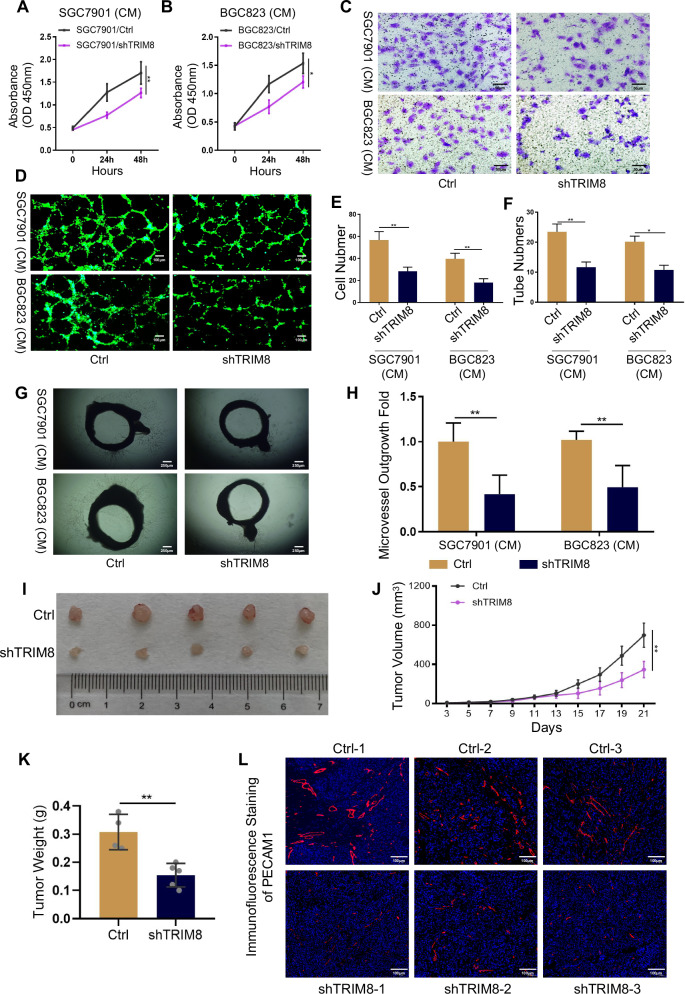
TRIM8 promotes angiogenesis in vitro and in vivo. **A**, **B** CCK-8 assay of HUVECs co-cultured with GC cells transduced with shTRIM8 or control. *, p < 0.05, **, p < 0.01. **C** Transwell assay on HUVECs to investigate the effect of CM from GC cells transduced with shTRIM8 or control. Scale bar = 50 μm. Magnification, ×100. Data shown is representative of 3 independent experiments. **, p < 0.01. **D** In vitro tube formation to investigate the effect of CM from GC cells transduced with shTRIM8 or control. Scale bar = 100μm. Magnification, ×200. **E** Quantification of (**C**). **, p < 0.01. **F** Quantification of (**D**). *, p < 0.05, **, p < 0.01. **G**, **H** Ring sections were cut out from freshly isolated aorta of euthanized rats. Representative images and quantification of the rat aortic ring assay treated with GC cells transduced with shTRIM8 or control. Scale bar = 250 μm. Magnification, ×40. **, *p* < 0.01. **I** Images of subcutaneous tumors formed by SGC7901/shTRIM8 and control SGC7901 cells. **J** Growth curve of tumors described in (**G**). **K** Average tumor weights from nude mice. **L** PECAM1 immunofluorescence of tumors described in (**I**).

To study the function of TRIM8 in GC angiogenesis, we used plasmid systems to knock down TRIM8 in SGC7901 and BGC823 cells, two common GC cell lines. Conditional medium (CM) from SGC7901 and BGC823 cells stably silencing TRIM8 decreased HUVEC growth (Fig. 2A, B). Moreover, Transwell assays confirmed that CM from both cell lines with stable TRIM8 silencing decreased HUVEC migration (Fig. 2C, E). Tumor cells may regulate endothelial cell tube formation by altering their proliferation and migration. To examine this possibility, we performed endothelial cell tube formation assays. Compared with controls, CM from SGC7901/shTRIM8 and BGC823/shTRIM8 cells elicited significantly reduced tube formation by HUVECs, as reflected by reduced tube-like structures or meshes and cell connection nodes on Matrigel (Fig. 2D, F). We next investigated whether TRIM8 promotes blood vessel growth in ex vivo models of angiogenesis. Rat aortic rings were prepared using tissue from wild-type rats. Compared with those in control GC cells, vessel outgrowth and branching from rat aortic rings in the absence of other added growth factors were significantly decreased after the addition of CM from SGC7901/shTRIM8 and BGC823/shTRIM8 cell lines (Fig. 2G, H). We further verified the in vivo proangiogenic function of TRIM8 in GC via a xenograft mouse model. Compared with control mice, mice injected with TRIM8-knockdown GC cells presented significantly decreased tumor progression, as reflected by reduced tumor size and weight (Fig. 2I–K). Furthermore, two-dimensional (2D) colony formation assay and CCK-8 assay demonstrated that TRIM8 knockdown significantly inhibited the proliferation of SGC7901 and BGC823 cells (Fig. S1C, D). Similarly, IF of PECAM1 revealed that the silencing of TRIM8 led to a lower vessel density in the tumors (Fig. 2L). Taken together, these in vitro and in vivo results reveal a critical role of TRIM8 in the regulation of GC angiogenesis.

### TRIM8 promotes K63-linked ubiquitination of PGK1 and its stability

**Fig. 3:**
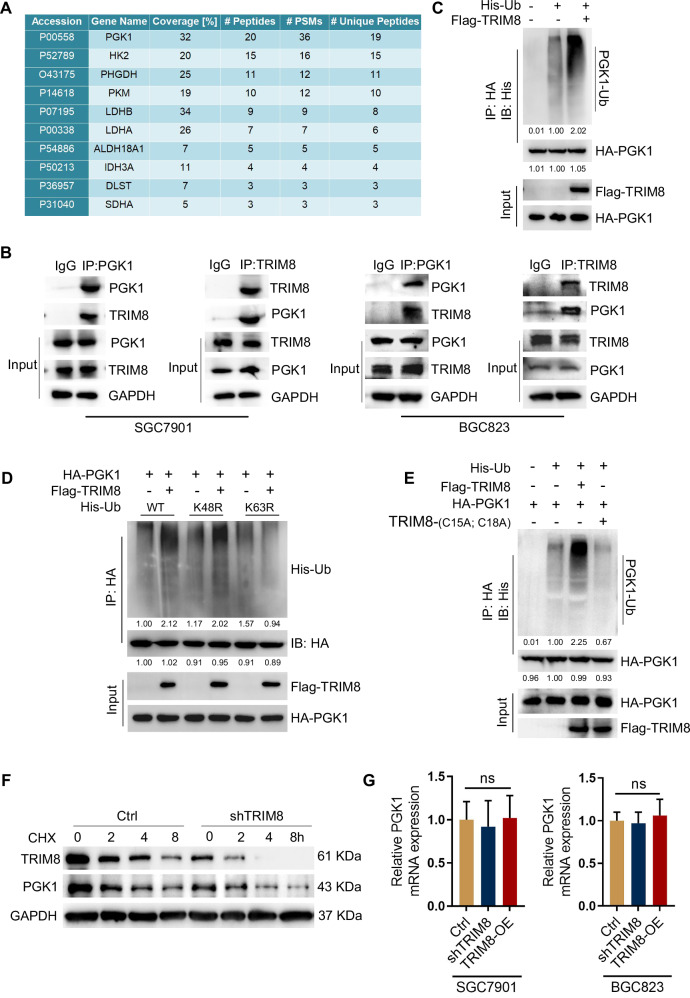
TRIM8 promotes K63-linked ubiquitination of PGK1 and its stability. **A** Mass spectrometric analysis of TRIM8-binding proteins commonly related to metabolism in SGC7901 cells. **B** Endogenous co-immunoprecipitation of TRIM8 and PGK1 in SGC7901 cells and BGC823 cells. **C** SGC7901 cells were transfected with indicated plasmids for ubiquitination assay. **D** In screening for potential lysine ubiquitination types, the ubiquitination of HA-PGK1 in response to TRIM8 overexpression was examined in GC cells transfected with the wild-type (WT) and mutated His-Ub plasmids. K63R, Ub only Lys63 residue was mutated. K48R, Ub only Lys48 residue was mutated. **E** An immunoprecipitation assay was used to examine PGK1 ubiquitination in HEK293T cells that were transfected with HA-PGK1, His-Ub, Flag-TRIM8, and/or a Flag-TRIM8 mutant (C15A, C18A). **F** Representative WB analyses of PGK1 expression in shTRIM8 SGC7901 cells that were treated with the protein synthesis inhibitor cycloheximide (CHX; 50 µg/ml) for 8 h before extraction. **G** Real-time qPCR of PGK1 mRNA levels after silencing of TRIM8 in SGC7901 and BGC823 cell lines.

To examine the metabolism-related mechanism underlying TRIM8-mediated angiogenesis, we immunoprecipitated TRIM8 from SGC7901 cells and analyzed the TRIM8-associated metabolic proteins by mass spectrometry analysis (Supplementary Table 2). We showed that PGK1, a master metabolic enzyme of glycolysis, was a TRIM8-interacting protein (Fig. 3A). This interaction was validated by coimmunoprecipitation analyses, which revealed that endogenous TRIM8 was associated with endogenous PGK1 in SGC7901 and BGC823 cells (Fig. 3B). In addition, ectopic expression of the TRIM8 E3 ligase readily induced the ubiquitination of PGK1 (Fig. 3C). Knockdown of TRIM8 in SGC7901 and BGC823 cells lead to decreased PGK1 ubiquitination (Fig. S2A). These results indicate that TRIM8 directly binds to PGK1 and promotes its ubiquitination.

K48 and K63 ubiquitin linkages are the most abundant ubiquitin linkage types and account for ~80% of the total ubiquitin linkages in mammalian cells. Cellular proteins conjugated to K48-linked Ub chains are targeted to proteasomes for degradation, whereas proteins conjugated to K63-ubiquitin chains are protected from proteasome-mediated degradation, regulating lysosomal functions and the inflammatory response [29, 30]. Screening of mutated forms of ubiquitin for potential lysine ubiquitination types revealed that both wild-type (WT) Ub and K63 (Ub with the intact Lys63 residue alone) could be linked to PGK1 by TRIM8 (Fig. S2B, C), but not K48 (Ub with the intact Lys48 residue alone) (Fig. S2B). Moreover, TRIM8 failed to link K63R (Ub only Lys63 residue was mutated) to PGK1(Fig. 3D and Fig. S2C). These data reveal that TRIM8 promote only K63-linked, not K48-linked, ubiquitination of PGK1. Consistent with these findings, an analysis of PGK1 decay in the presence of the translational inhibitor cycloheximide revealed that deletion of TRIM8 decreased PGK1 protein expression during treatment (Fig. 3F). In contrast, the degradation of the PGK1 protein was decelerated during treatment under TRIM8 overexpression (Fig. S2D). Besides, both TRIM8 knockdown and overexpression failed to alter PGK1 gene (mRNA) expression (Fig. 3G). The RING domain of TRIM8 is responsible for its E3 ligase activity [31]. We demonstrated that TRIM8, not the TRIM8-(C15A; C18A) E3 ligase dead mutant, induced PGK1 ubiquitination in vitro (Fig. 3E). These results confirm that TRIM8 is involved in PGK1 protein stability. Together, these data suggest that TRIM8 is a direct E3 ligase for PGK1 and promotes its stability.

### K63-ubiquitination of PGK1 promotes glycolysis and angiogenesis in GC cells

**Fig. 4:**
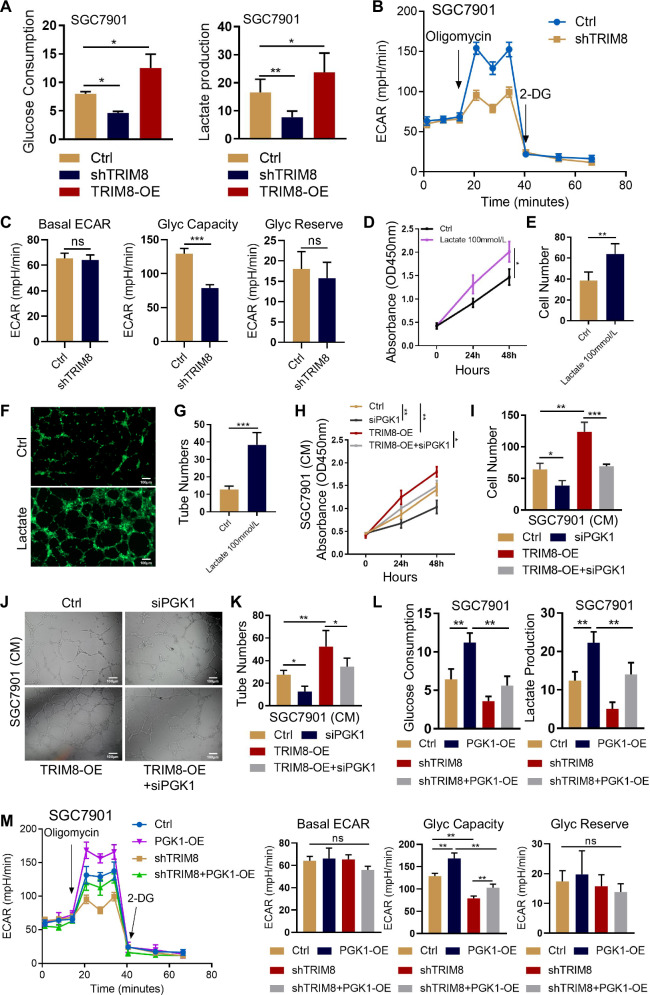
K63- ubiquitination of PGK1 promotes glycolysis and angiogenesis in GC cells. **A** SGC7901 cells with or without TRIM8 depletion and TRIM8 overexpression were analyzed for glucose consumption and lactate production. **B** The glycolytic rate of cells in (**A**) was measured by Seahorse. **C** Quantification of (**B**). Basal ECAR: Basic glycolysis rate. Glyc Capacity: The maximal glycolytic rate induced by oligomycin-mediated inhibition of mitochondrial respiration. Glyc Reserve: The additional acidification capacity induced by the glucose analog 2-DG. ***, p < 0.001. ns non-significant. **D** CCK-8 assay of HUVECs treated with 100 mM/L lactate. *, p < 0.05. **E** Transwell assay on HUVECs to investigate the effect of 100 mM/L lactate. Data shown is representative of 3 independent experiments. **, p < 0.01. **F** Tube formation of HUVECs was shown in representative images after treated with lactate. Scale bar = 100 μm. Magnification, ×200. **G** Quantification of (**F**). ***, p < 0.001. **H** SGC7901 cells stably overexpressing TRIM8 or control vector were transfected with control or PGK1 siRNA. CCK-8 assay of HUVECs co-cultured with SGC7901 cells transduced with indicated vectors and plasmids. *, p < 0.05, **, p < 0.01. **I** SGC7901 cells stably overexpressing TRIM8 or control vector were transfected with control or PGK1 siRNA. Transwell assay of HUVECs co-cultured with SGC7901 cells transduced with indicated vectors and plasmids. Data shown is representative of 3 independent experiments. *, p < 0.05, **, p < 0.01. ***, p < 0.001. **J** SGC7901 cells stably overexpressing TRIM8 or control vector were transfected with control or PGK1 siRNA. Tube formation of HUVECs co-cultured with SGC7901 cells transduced with indicated vectors and plasmids was shown in representative images. Scale bar = 100 μm. Magnification, ×200. **K** Quantification of (**J**). *, p < 0.05, **, p < 0.01. **L** SGC7901 Cells stably overexpressing PGK1 or control vector were transfected with control or TRIM8 shRNA. Cells were analyzed for glucose consumption and lactate production. Data shown is representative of three independent experiments. **M**, **N** SGC7901 Cells stably overexpressing PGK1 or control vector were transfected with control or TRIM8 shRNA. The glycolytic rate of cells was measured by Seahorse. Data shown is representative of three independent experiments. **, p < 0.01. ns non-significant.

Glycolysis is critical for energy production in tumor cells and is the fundamental basis for biosynthesis. Phosphoglycerate kinase 1 (PGK1) is an important enzyme in the metabolic glycolysis pathway. To determine the functional consequences of TRIM8-mediated PGK1 K63-ubiquitination, we examined the effect of ubiquitination on glycolysis in GC cells. We showed that PGK1-induced glycolysis was much lower in GC cells with silenced TRIM8 than in those with its control counterpart, as evidenced by lower glucose uptake and lactate production (Fig. 4A, Fig. S3A). These results were further supported by the real-time extracellular acidification rate (ECAR), consistently showing that the TRIM8-depleted GC cells, unlike that of control cells, was unable to promote the glycolytic rate (Fig. 4B‒C, Fig. S3B‒C). The metabolic reprogramming via aerobic glycolysis drives lactate overproduction. Several studies have shown that accumulated lactate acts as a critical regulator of endothelial cell migration, tube formation, and tumor angiogenesis [32, 33]. Consistently, treatment of HUVECs with lactate promoted endothelial cell growth and migration, resulting in increased tube formation (Fig. 4D–G). To determine whether PGK1 K63 ubiquitination-induced lactate accumulation affects tumor angiogenesis, we performed endothelial cell migration and tube formation assays to test whether PGK1 silencing would recapitulate the effect of TRIM8 suppression on angiogenesis. Consistent with the known impact of PGK1 on glycolysis and lactate accumulation, knockdown of PGK1 in GC cells triggered a significant decrease in endothelial cell migration and tube formation. PGK1 silencing, which resulted in reduced K63-ubiquitinated PGK1, counteracted the proangiogenic effects of TRIM8 overexpression (Fig. 4H–K, Fig. S3D–G). These results suggest that K63 ubiquitination is critical for the PGK1 proangiogenic phenotype in gastric cancer. Of note, the overexpression of PGK1, which has higher glycolytic activity compared to its control counterpart, did not counteract the impaired glycolysis of GC cells induced by TRIM8 silencing, as evidenced by the inadequate increase in glucose uptake and lactate production (Fig. 4L, Fig. S3H–I). In concordance with these observations, ECAR confirmed that silencing TRIM8 suppressed the ability of PGK1 overexpression to enhance the glycolytic rate in GC cells (Fig. 4M, N, Fig. S3J). Overall, our data support K63-ubiquitinated PGK1 as a key mediator of TRIM8 proangiogenic effect and there may be other factors involved in TRIM8-mediated glycolysis.

### ACAT1-mediated PGK1 acetylation promotes PGK1 activity, GC cell glycolysis and tumor angiogenesis

**Fig. 5:**
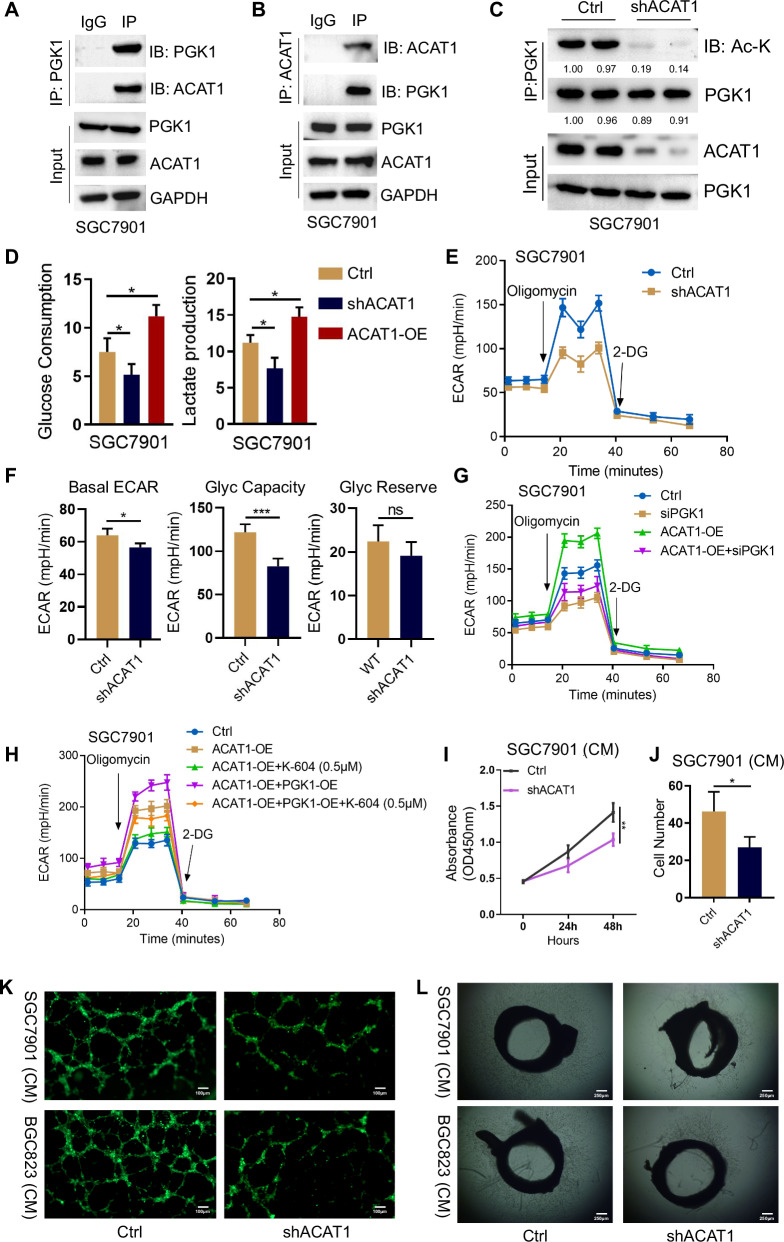
ACAT1 -mediated PGK1 acetylation promotes PGK1 activity, GC cell glycolysis and tumor angiogenesis. **A** PGK1 immunoprecipitation from whole cell lysates of SGC7901 cells followed by Western blot with ACAT1 and PGK1 antibody. **B** ACAT1 immunoprecipitation from whole cell lysates of SGC7901 cells followed by Western blot with ACAT1 and PGK1 antibody. **C** PGK1 immunoprecipitation from whole cell lysates of SGC7901 cells transfected with control or ACAT1 shRNA followed by Western blot with ACAT1 and Ac- lysine antibody. Input shows no effect of ACAT1 knockdown on PGK1 expression. **D** SGC7901 cells with or without ACAT1 depletion and ACAT1 overexpression were analyzed for glucose consumption and lactate production. **E** The glycolytic rate of cells in (**D**) was measured by Seahorse. **F** Quantification of (**E**). *, p < 0.05, ***, p < 0.001. ns non-significant. **G** The glycolytic rate of SGC7901 cells transfected with indicated plasmids was measured by Seahorse. **H** The glycolytic rate of SGC7901 cells transfected with indicated plasmids was measured by Seahorse. Cells were pretreated with or without K-604(0.5 μM), a potent and selective ACAT1 enzyme activity inhibitor. **I** CCK-8 assay of HUVECs co-cultured with SGC7901 cells transduced with control or ACAT1 shRNA. **J** Transwell assay on HUVECs co-cultured with SGC7901 cells transfected with control or ACAT1 shRNA. **K** Tube formation of HUVECs co-cultured with GC cells transduced with control or ACAT1 shRNA was shown in representative images. Scale bar = 100 μm. Magnification, ×200. **L** Representative images of the rat aortic ring assay treated with GC cells transduced with shACAT1 or control. Scale bar = 250 μm. Magnification, ×40.

Posttranslational modification is a major means to modulate PGK1 glycolytic enzyme activity [34]. Multiple acetylation sites have been identified on PGK1 through large-scale studies [35, 36]. Mass spectrometry analysis identified a candidate list of acetyltransferases may be responsible for TRIM8-mediated PGK1 glycolytic enzyme activity, which include Acyl-CoA cholesterol acyltransferase 1(ACAT1), N-alpha-acetyltransferase 10(NAA10), N-alpha-acetyltransferase 15(NAA15), dihydrolipoamide acetyltransferase(DLAT) and histone acetyltransferase 1(HAT1). Immunoprecipitation (IP) followed by Western blot showed ACAT1 interacted with PGK1 (Fig. 5A, B) and promoted PGK1 acetylation (Fig. 5C). To determine the role of ACAT1 in PGK1 glycolytic enzyme activity, we generated GC cells stably transduced with lentiviral particles carrying either a shRNA targeting ACAT1 (shACAT1) or a control or cells stably overexpressing ACAT1 (ACAT1-OE). Glucose and lactate assays showed that ACAT1 knockdown significantly abrogated glycolysis in GC cells, as evidenced by altered glucose consumption and lactate production (Fig. 5D, Fig. S4A). These results were further supported by ECAR, consistently showing that ACAT1 knockdown was able to decrease the glycolytic rate of GC cells (Fig. 5E, F, Fig. S4B, C). We next investigated whether ACAT1 ectopic expression is able to promote glycolysis and if acetylation of PGK1 contributes to that effect. Silencing of PGK1 strongly counteracted the promoting glycolysis effect of ACAT1 overexpression (Fig. 5G, Fig. S4D–F). To confirm whether the promoting glycolysis effect of ACAT1 overexpression were dependent on acetylation of PGK1, GC cells were pretreated with K-604 dihydrochloride, a potent and selective ACAT1 enzyme activity inhibitor. As expected, the promoting glycolysis effect induced by ACAT1 overexpression was ablated by treatment of K-604 (Fig. 5H, Fig. S5A–C). Overall, our data support acetylated PGK1 as a key mediator of ACAT1 pro-glycolytic effect. We next examined the effects of ACAT1 in GC cells on tumor angiogenesis. As expected, ACAT1 depletion resulted in decreased tumor angiogenesis, as detected by decreased endothelial cell proliferation, migration and tube formation (Fig. 5I–K, Fig. S5D–F). Compared with those of their control counterparts, vessel outgrowth and branching from rat aortic rings in the absence of other added growth factors were significantly decreased after the addition of CM from SGC7901/shACAT1 and BGC823/shACAT1 cells (Fig. 5L, Fig. S5G). These results indicate that ACAT1-mediated PGK1 acetylation is specifically required for PGK1 glycolytic enzyme activity, glycolysis, and tumor angiogenesis.

### ACAT1 recruitment and PGK1 acetylation are dependent on TRIM8-mediated PGK1 K63-linked ubiquitination

Posttranslational modifications often result in alterations in protein structure and subsequent protein‒protein interactions [37]. K63-linked ubiquitination-modified proteins interact with acetyltransferases [38], and ubiquitin E3 ligases promote the recruitment of acetyltransferase to form protein complexes [39], specifically regulating acetylation modification.

**Fig. 6:**
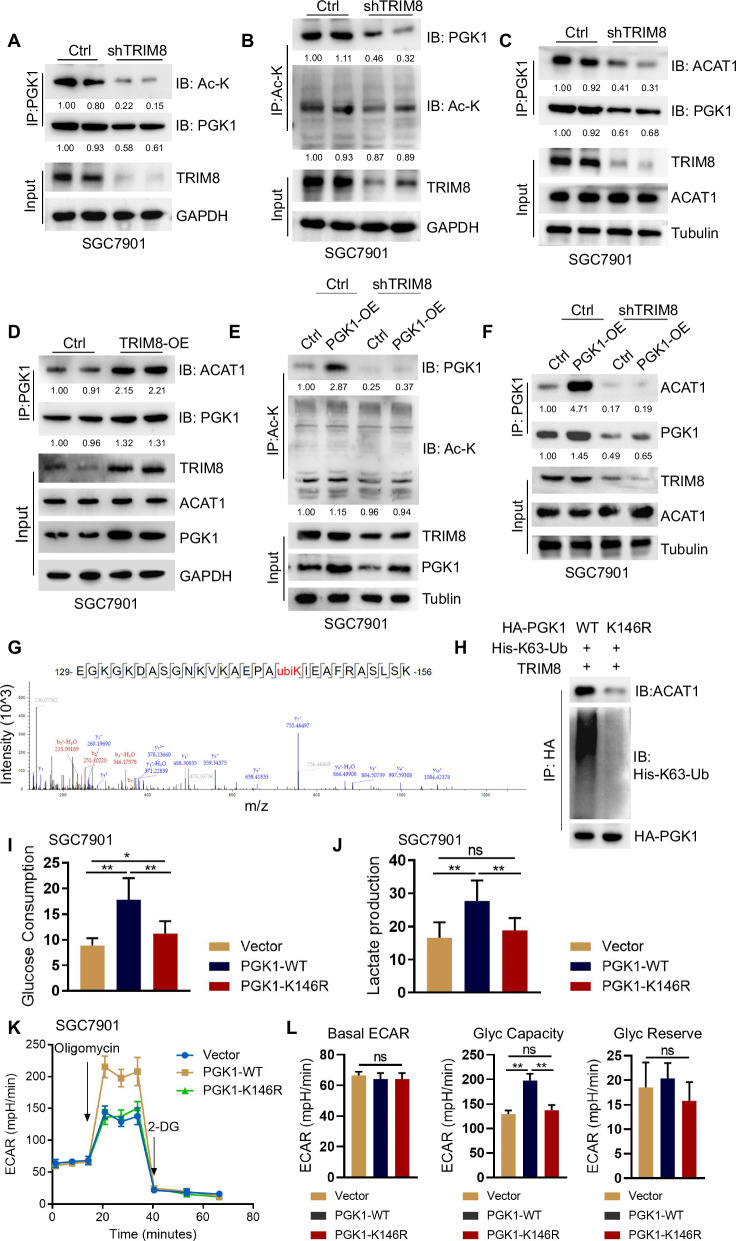
ACAT1 recruitment and PGK1 acetylation are dependent on TRIM8-mediated PGK1 K63-linked ubiquitination. **A** PGK1 immunoprecipitation from whole cell lysates of SGC7901 cells transfected with control or TRIM8 shRNA followed by Western blot with Ac- lysine and PGK1 antibodies. **B** Ac- lysine immunoprecipitation from whole cell lysates of SGC7901 cells transfected with control or TRIM8 shRNA followed by Western blot with PGK1 and Ac- lysine antibodies. **C** PGK1 immunoprecipitation from whole cell lysates of SGC7901 cells transfected with control or TRIM8 shRNA followed by Western blot with ACAT1 and PGK1 antibodies. Input shows no effect of TRIM8 knockdown on ACAT1 expression. **D** PGK1 immunoprecipitation from whole cell lysates of SGC7901 cells SGC7901 cells stably overexpressing TRIM8 or control vector followed by Western blot with ACAT1 and PGK1 antibodies. **E** Western blot of PGK1 and TRIM8 in SGC7901 cells stably overexpressing PGK1 or control vector and transfected with control or TRIM8 shRNA. Ac- lysine IP on SGC7901 cells lysates stably overexpressing PGK1 or control vector and transfected with control or TRIM8 shRNA followed by Western blot with PGK1 and Ac- lysine antibodies. **F** Western blot of ACAT1 and TRIM8 in SGC7901 cells stably overexpressing PGK1 or control vector and transfected with control or TRIM8 shRNA. PGK1 IP on SGC7901 cells lysates stably overexpressing PGK1 or control vector and transfected with control or TRIM8 shRNA followed by Western blot with PGK1 and ACAT1 antibodies. Input shows no effect of TRIM8 knockdown or overexpressing PGK1 on ACAT1 expression. **G** Mass spectrometric analysis was performed of a tryptic fragment. The results suggested that TRIM8 preferentially ubiquitinates PGK1 at K146. **H** Immunoprecipitation assay was performed for the SGC7901 cells transfected with PGK1 WT or K146R to access its K63-ubiquitination and binding with ACAT1. SGC7901 cells transfected with PGK1 WT or K146R were analyzed for glucose consumption (**I**) and lactate production (**J**). **K**–**L** The glycolytic rate of cells in (**I**) was measured by Seahorse. **, p < 0.01. ns non-significant.

To determine whether TRIM8-mediated K63-ubiquitination regulates PGK1 acetylation, we performed immunoblotting with an Ac-lysine antibody. IP of PGK1 followed by Western blot revealed that Ac-lysine binding to the PGK1 protein was lower in shTRIM8-transfected SGC7901 cells than in control cells (Fig. 6A).

Ac-lysine enrichment followed by Western blot showed reduced PGK1 in shTRIM8- compared to control cells, consistent with lower acetylation on PGK1 proteins (Fig. 6B). Mechanistically, the endogenous interaction between PGK1 and ACAT1 was markedly reduced in TRIM8-knockdown GC cells while the ACAT1 protein level remained unaffected by TRIM8 knockdown (Fig. 6C). In contrast, the endogenous interaction between PGK1 and ACAT1 was markedly increased in TRIM8-overexpressed GC cells while the ACAT1 protein level remained unaffected by TRIM8-overexpression (Fig. 6D, Figure S6A). Remarkably, TRIM8 deficiency in GC cell lines impaired PGK1 acetylation upon PGK1 overexpression (Fig. 6E). The endogenous interaction between PGK1 and ACAT1 upon PGK1 overexpression was markedly reduced in TRIM8-silenced GC cells (Fig. 6F). Hence, TRIM8 serves as an essential regulator of PGK1 activity by recruiting ACAT1 to PGK1 and subsequently acetylating PGK1. These results indicate that PGK1 acetylation is specifically mediated by TRIM8. To directly investigate the functional role of TRIM8-mediated K63-linked ubiquitination of PGK1 in these processes, we next determined the TRIM8-dependent ubiquitination site(s) on PGK1. Mass spectrometry analysis of immunoprecipitated PGK1 showed that it was ubiquitinated at K146 by TRIM8 (Fig. 6G). Mutation of K146R in PGK1 also reduced the in vitro ubiquitination of PGK1 by TRIM8. In the presence of TRIM8, PGK1 that is deficient in K63-linked ubiquitination also did not interact with ACAT1 in the co-immunoprecipitation assay (Fig. 6H) and was insufficient to induce enhanced PGK1 glycolytic activity in GC cells (Fig. 6I–L, Fig. S6B–E). These results indicate that the binding of PGK1 to ACAT1 is specifically mediated by TRIM8 and its induced K63- ubiquitination. Together, these data suggest that K63-linked PGK1 ubiquitination at K146 by TRIM8 is needed for ACAT1 recruitment, PGK1 acetylation, and PGK1 glycolytic activity.

## Discussion

Although it has long been speculated that the ubiquitin–proteasome system plays important roles in regulating angiogenesis of cancer [40], there are no comprehensive studies supporting this assertion. There is currently little understanding of how ubiquitin–proteasome system controls angiogenesis, hindering the development of ubiquitin-based therapeutic strategies. We demonstrated that TRIM8 stabilizes PGK1 through K63 ubiquitination and recruits ACAT1, driving PGK1 acetylation, thereby enhancing PGK1-mediated glycolysis and ultimately leading to lactate accumulation and tumor angiogenesis. Consequently, TRIM8-mediated PGK1 K63 ubiquitination and ACAT1-dependent PGK1 acetylation are required for glycolysis, which promotes lactate accumulation and tumor angiogenesis. Our findings uncover a novel mechanism by which TRIM8 promotes tumor angiogenesis by regulating tumor cell metabolism and implicates a therapeutic potential to disrupt the connection between metabolism and angiogenesis by inhibiting PGK1 K63 ubiquitination.

The E3 ligase TRIM8 plays important but divergent roles in various types of cancer via multiple mechanisms. TRIM8 has been shown to induce HCC progression through mediating the ubiquitination of hepatocyte nuclear factor 1α (HNF1α) and promoting its protein degradation [41]. TRIM8 also ubiquitinates and degrades EWS/FLI, a driver fusion-TF in Ewing sarcoma [22]. However, whether TRIM8 regulates tumor cell metabolism remains unknown. In this study, we demonstrated that TRIM8 regulates gastric cancer cell glycolysis by mediating PGK1 K63 ubiquitination. K63 ubiquitination stabilizes PGK1 and recruits ACAT1, driving PGK1 acetylation and promoting tumor angiogenesis in gastric cancer. These results indicate that these posttranslational modifications are important in GC metabolism and angiogenesis.

Metabolic enzymes are subjected to multiple types of PTMs, such as phosphorylation, ubiquitination and acetylation. PGK1 is K48 ubiquitinated and degraded by TRIM50, which inhibits glycolysis and the malignant progression of gastric cancer [22]. PGK1 is also acetylated at K323, which promotes its enzymatic activity and cancer cell metabolism, resulting in the promotion of liver cancer cell proliferation and tumorigenesis [34]. ARD1 acetylates PGK1 at K388, which does not affect glycolysis, is specifically required for glutamine deprivation-induced PGK1-mediated autophagy in glioblastoma [28]. In addition, we showed that K63 ubiquitination was required for PGK1-induced glycolysis and angiogenesis in gastric cancer cells expressing TRIM8, further supporting the essential role of TRIM8-mediated PGK1 K63 ubiquitination in the metabolism and angiogenesis of gastric cancer. Together, these findings indicate that PGK1 function can be regulated by different PTMs in response to various stimuli or the tumor microenvironment, which subsequently promote different aspects of cancer properties.

We identified K63 ubiquitination as a positive regulator of PGK1 acetylation. TRIM8 and its induced K63 ubiquitination promote the interaction of PGK1 with ACAT1, thereby facilitating PGK1 acetylation and downstream glycolysis for lactate accumulation and tumor angiogenesis. This finding is in agreement with previous evidence that TRAF6 E3 ligase and its induced K63 ubiquitination are required for p300 recruitment and p53 acetylation [39]. The E3 ligase TRIM8 and K63 ubiquitination promote acetyltransferase recruitment and acetylation highlights the crucial role of K63 ubiquitination as a PTM in PGK1 function and metabolism.

Solid tumors rely on a sustained vascular supply for oxygen and nutrient delivery to fuel rapid growth [5]. Cell metabolism reprogramming is one of the most important features of cancer and plays an integral role in the progression of cancer. Accordingly, tumor cells commonly display enhanced glycolytic activity under aerobic conditions, a metabolic hallmark termed the Warburg effect [6]. Glycolysis provides enough energy and metabolic intermediates to ensure the smooth progress of cell biosynthesis [42]. This elevated glycolytic flux results in significant lactate accumulation, thereby contributing to the tumor microenvironment and promoting tumor progression [43]. Lactate produced by glycolysis can accumulate in the tumor microenvironment, further stimulating the growth of endothelial cells and accelerating tumor angiogenesis and the metastasis of colorectal cancer [32]. We showed that TRIM8 and its induced K63 ubiquitination resulted in PGK1-dependent glycolysis and lactate accumulation in gastric cancer cells, which ultimately promoted tumor angiogenesis. These findings suggest that targeting metabolism is a potentially promising approach for cancer therapeutics. Approaches that inhibit TRIM8-mediated K63 ubiquitination and PGK1-regulated glycolysis likely increase the efficacy of tumor angiogenesis treatment.

In summary, our findings elucidate a novel mechanism underlying the upregulation of angiogenesis mediated by K63 ubiquitination-regulated glycolysis in tumor cells and provide a molecular basis for eliminating gastric cancer angiogenesis by targeting TRIM8-dependent PGK1 K63 ubiquitination.

## Conclusions

Our findings highlight the pro-angiogenic role of TRIM8 in gastric cancer through K63 ubiquitination regulated-glycolysis and providing a potential strategy to eliminate gastric cancer angiogenesis by targeting TRIM8 -dependent PGK1 K63 ubiquitination.

## Supplementary information

Supplementary Figure 1

Supplementary Figure 2

Supplementary Figure 3

Supplementary Figure 4

Supplementary Figure 5

Supplementary Figure 6

Supplementary Information

Supplementary Table 1

Supplementary Table 2. mass spectrometry analysis

Uncropped original Western blots

## Data Availability

The datasets used and/or analysed during the current study are available from the corresponding author on reasonable request.
