# Supplementary figures and images for: TRIM8-dependent K63-ubiquitinated PGK1 promotes glycolysis and angiogenesis in gastric cancer via interaction with ACAT1
Source: Cell Death Dis. 2025 Nov 3;16(1):780. doi: 10.1038/s41419-025-08015-y (PMC12583530; doi:10.1038/s41419-025-08015-y)

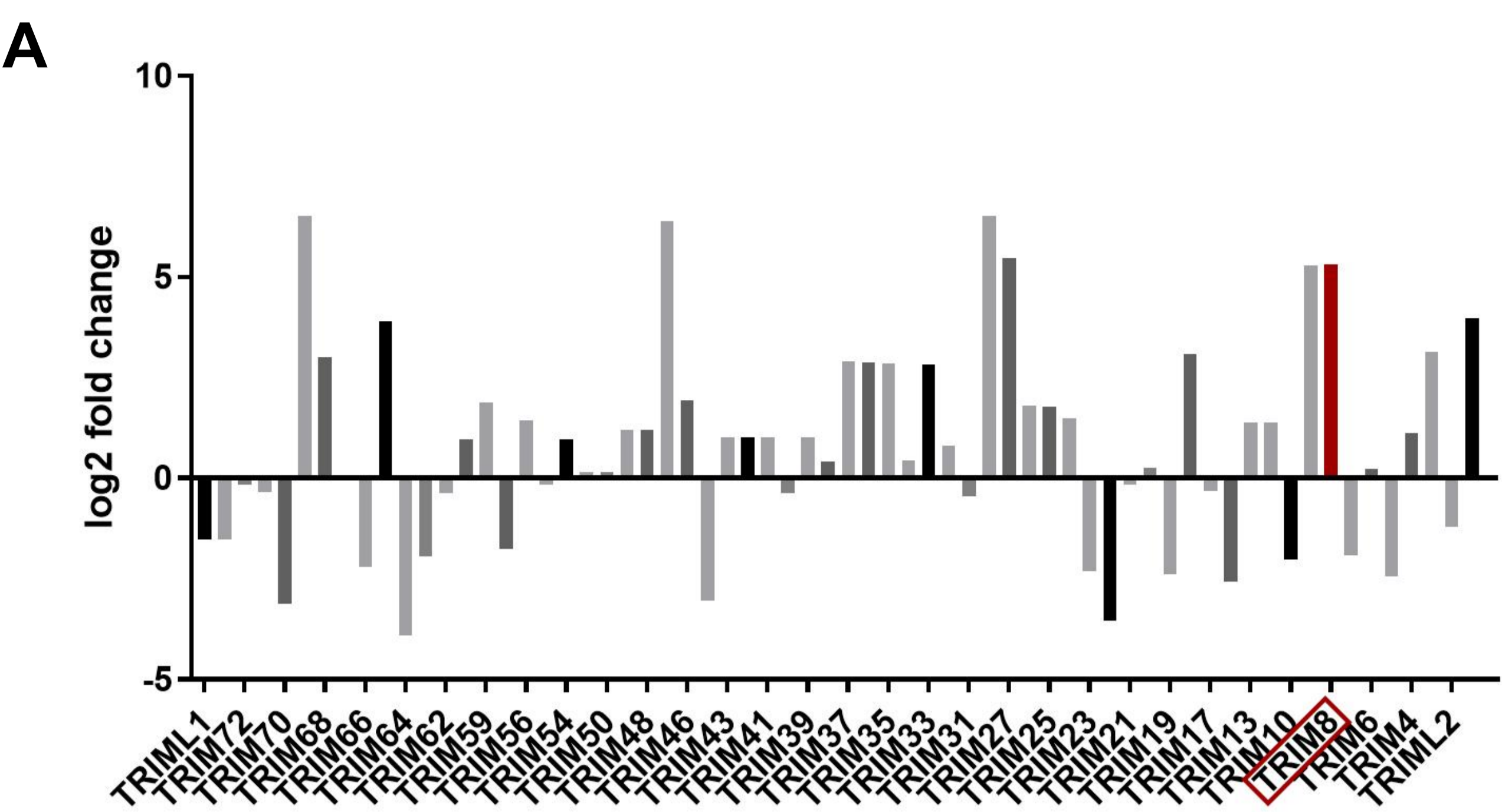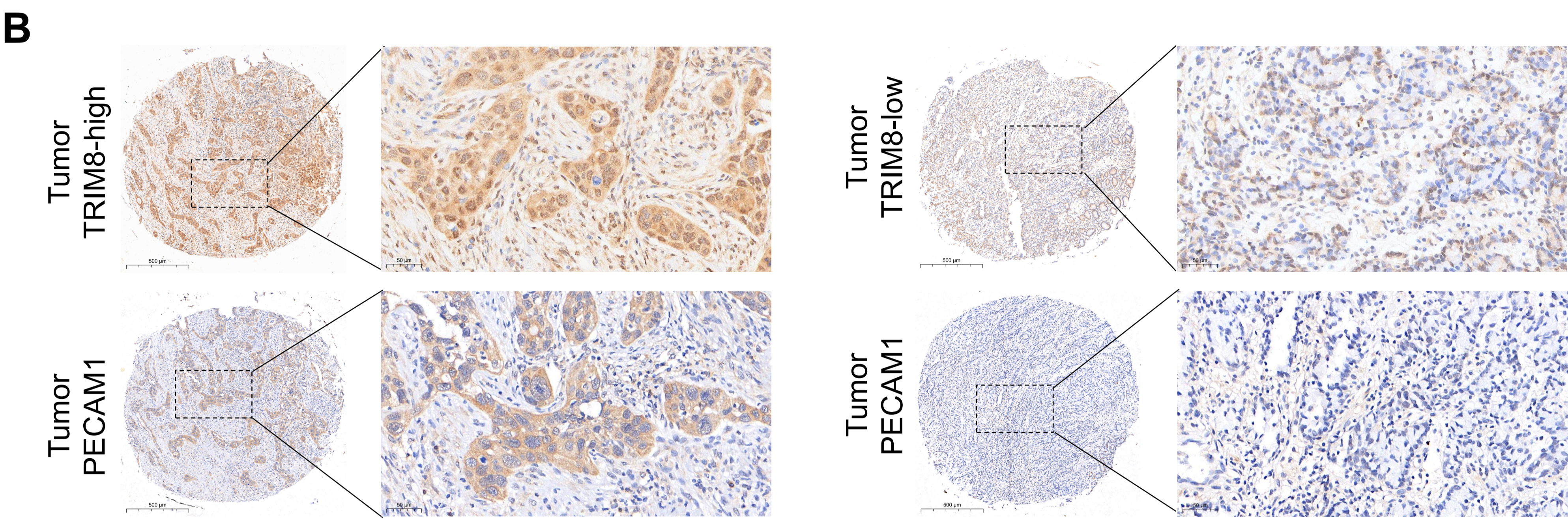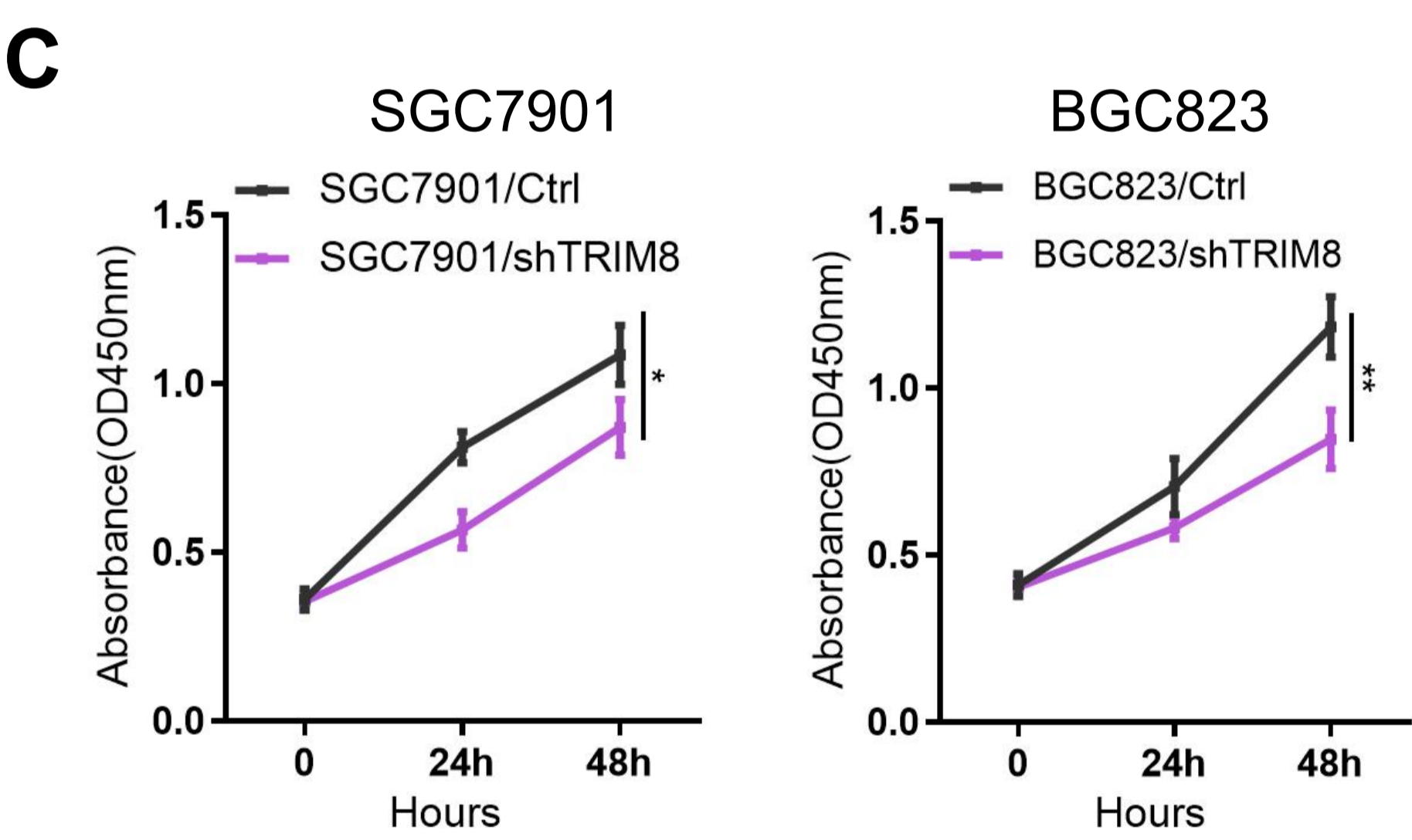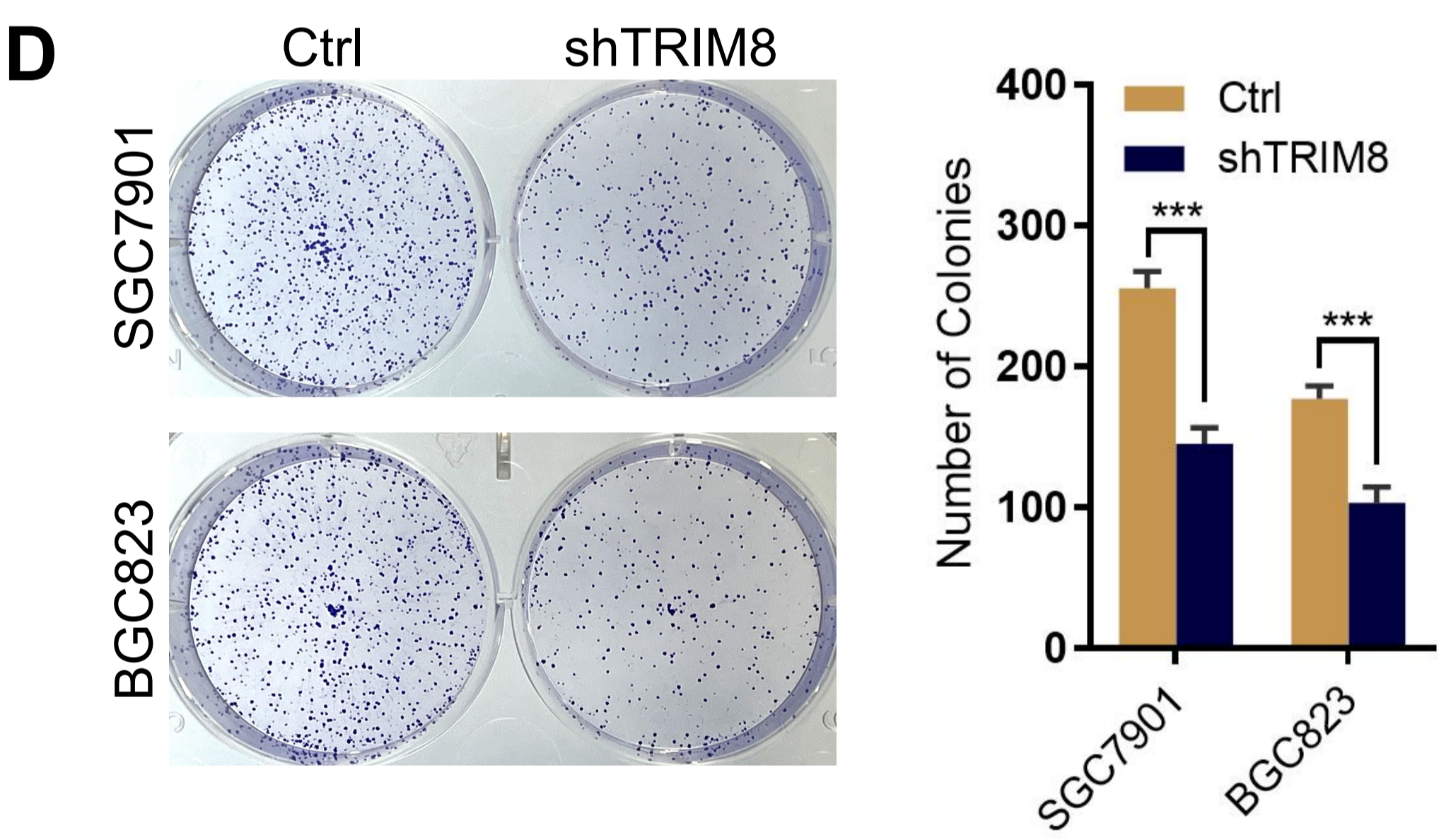

Supplement: Supplementary file 1 — Supplementary Figure 1 [file 41419_2025_8015_MOESM1_ESM.pdf]

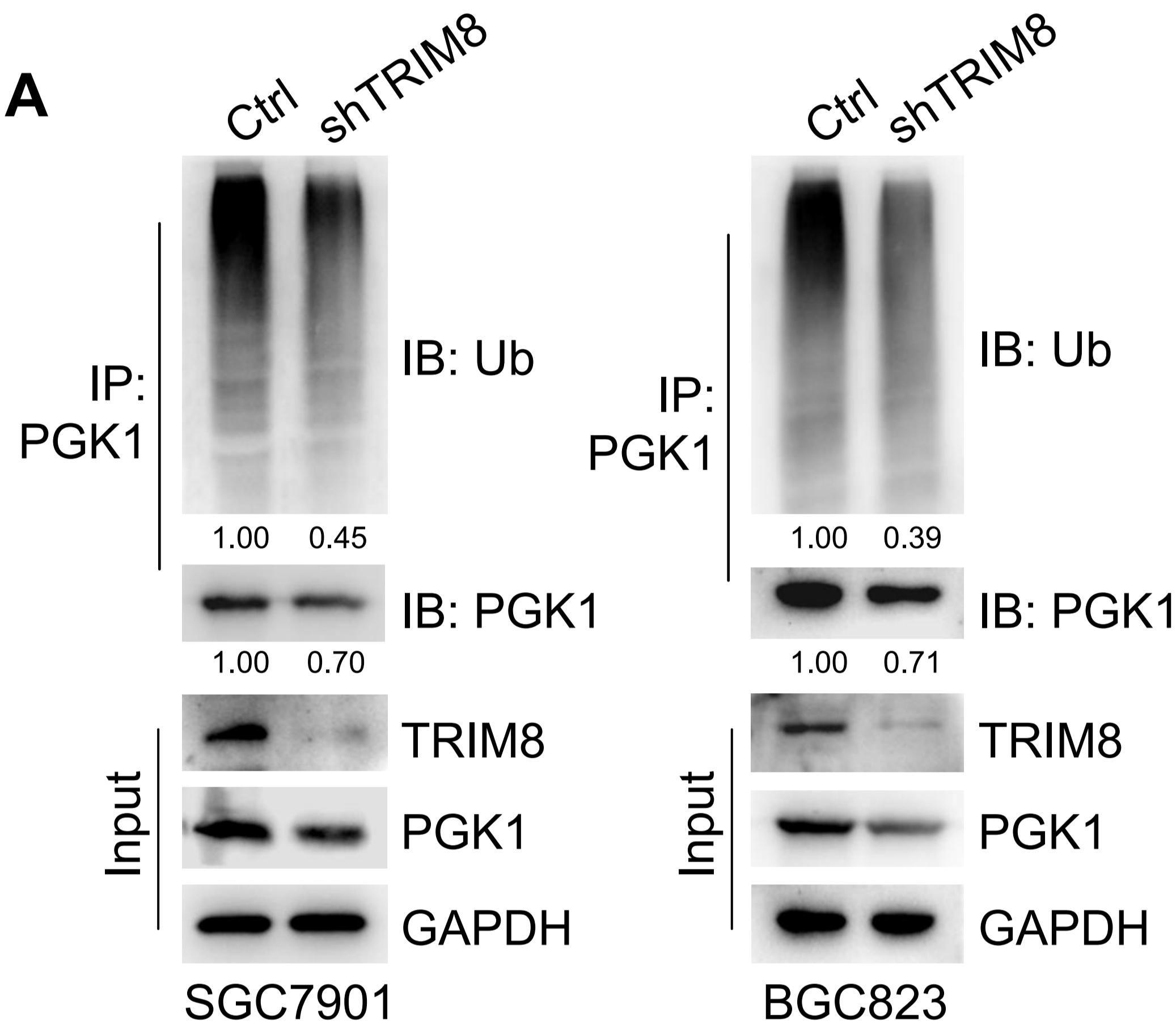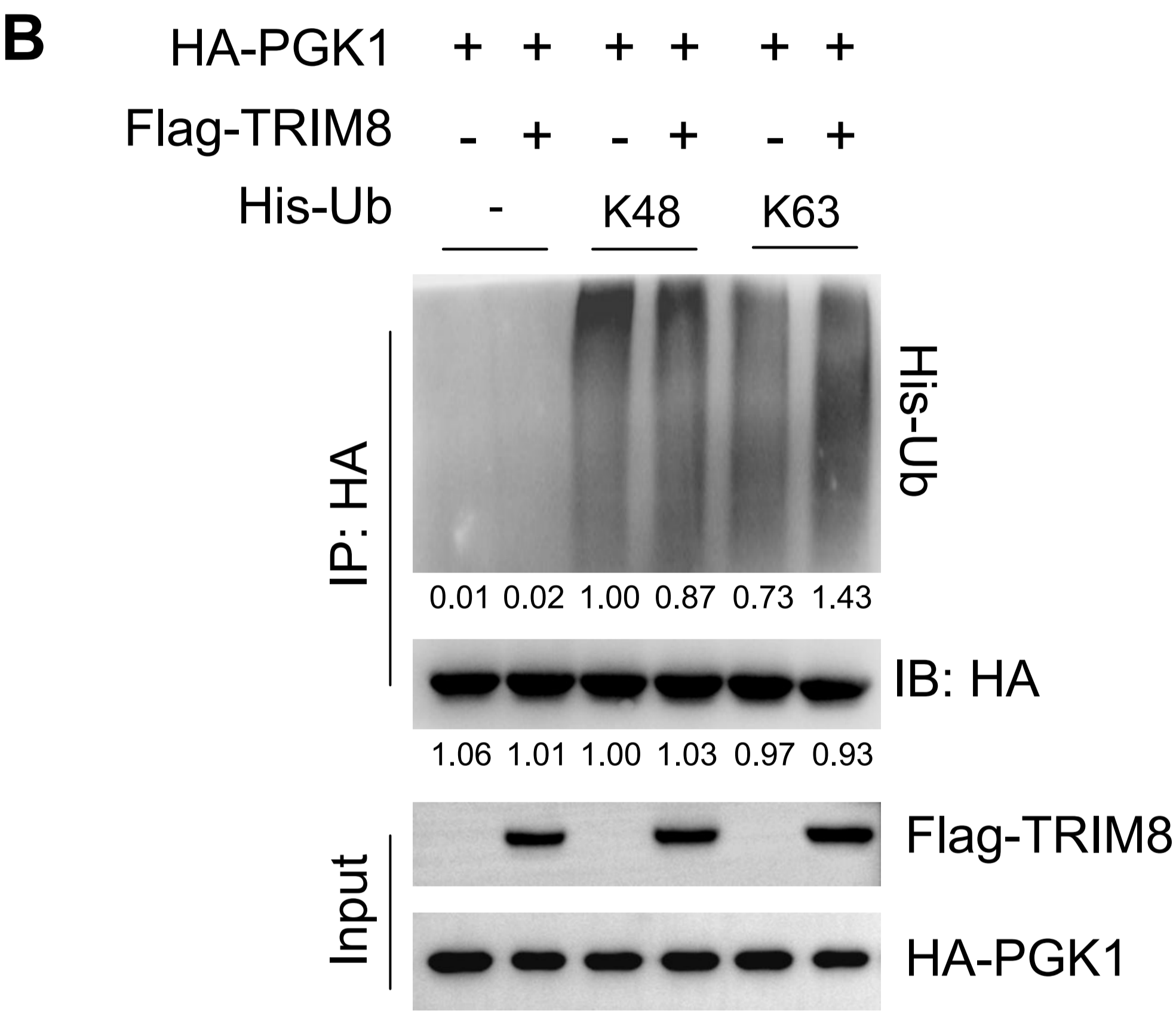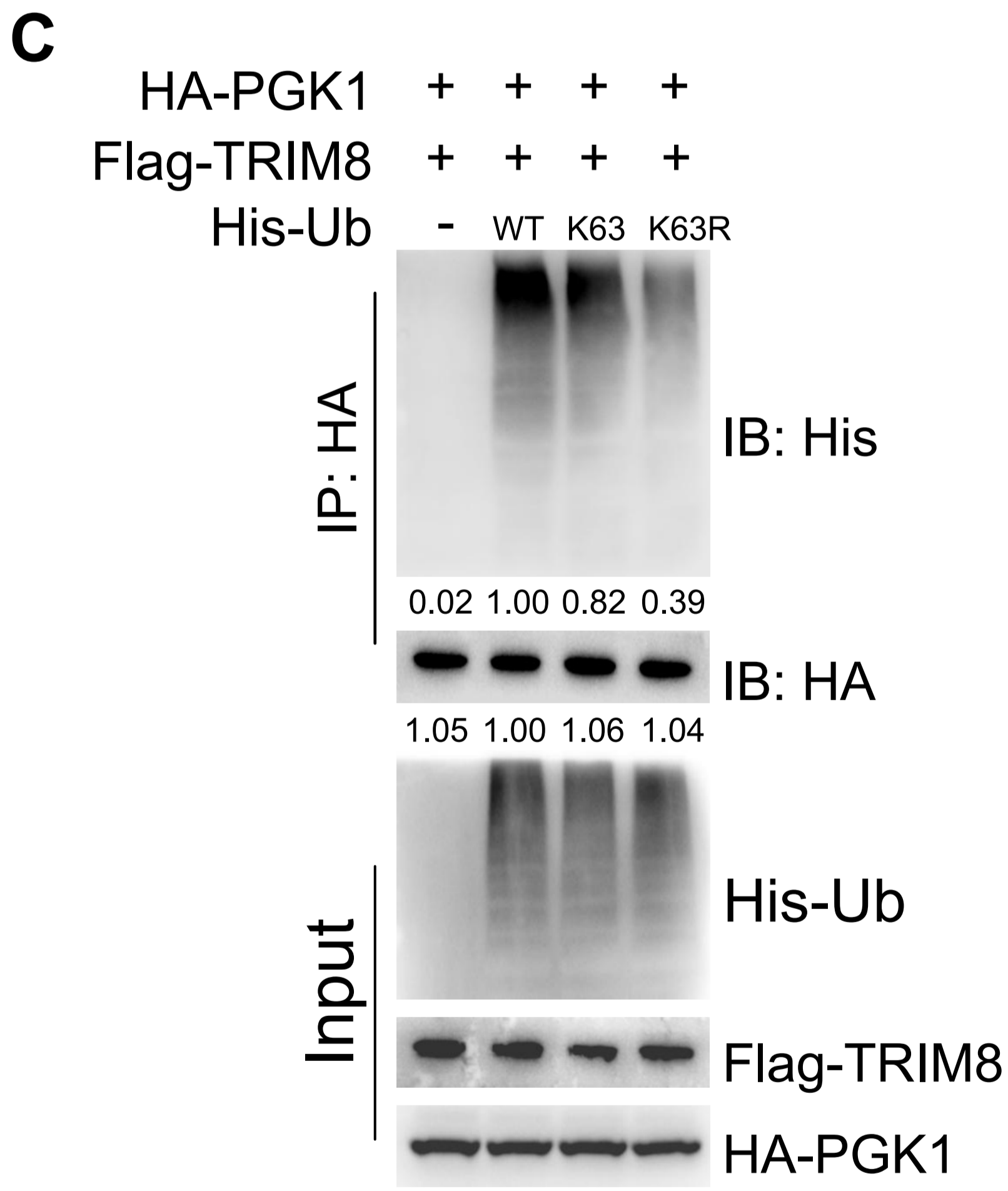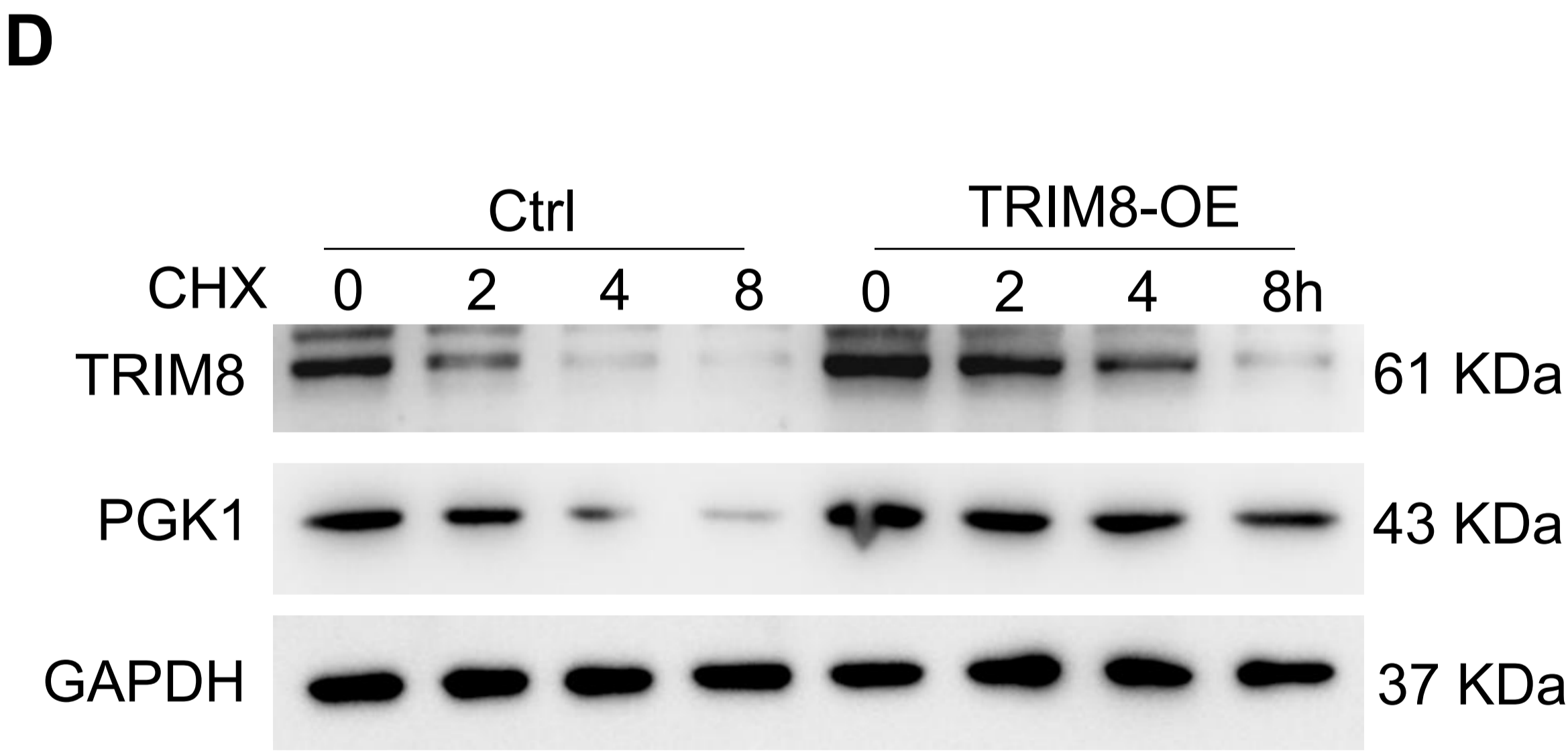

Supplement: Supplementary file 2 — Supplementary Figure 2 [file 41419_2025_8015_MOESM2_ESM.pdf]

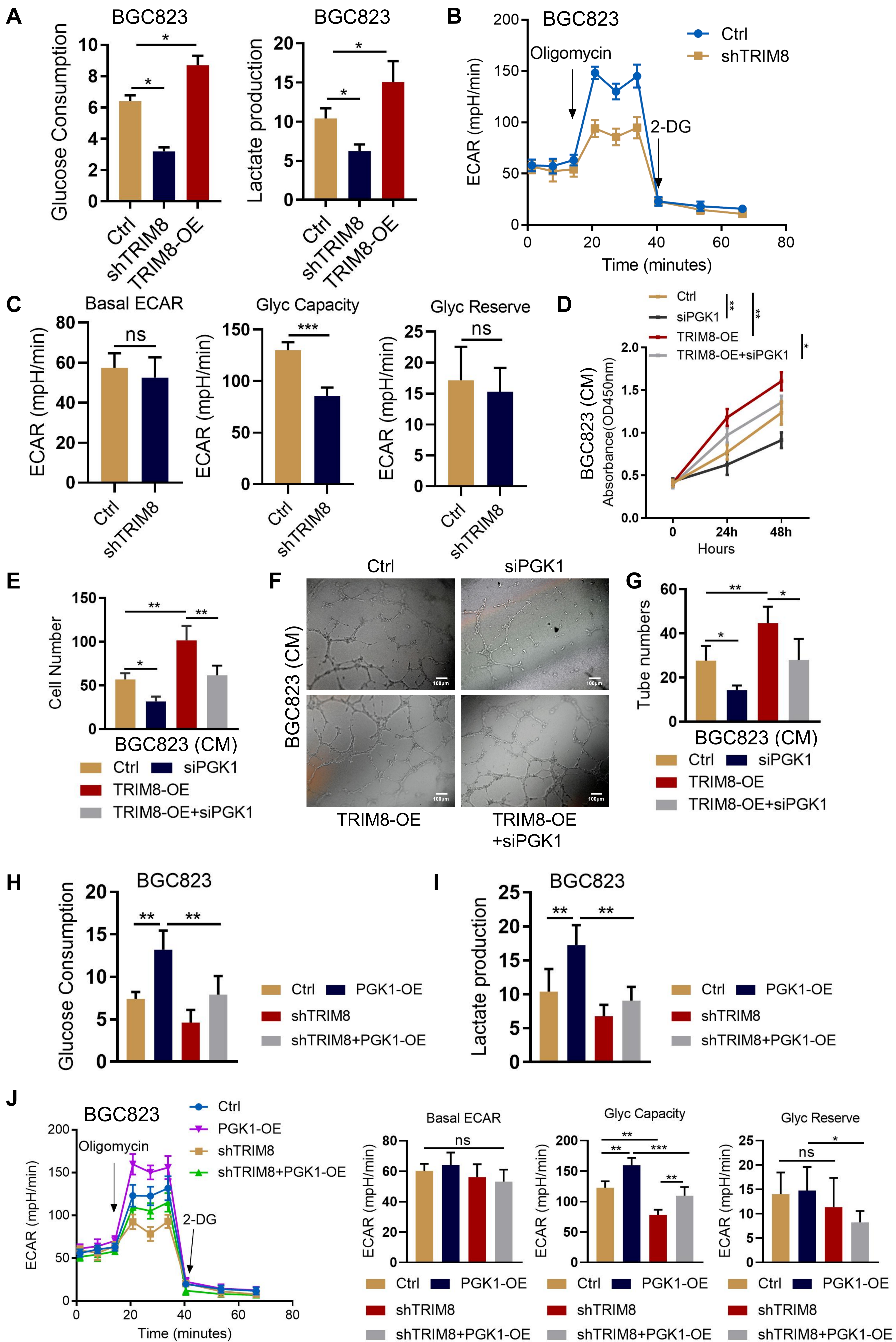

Supplement: Supplementary file 3 — Supplementary Figure 3 [file 41419_2025_8015_MOESM3_ESM.pdf]

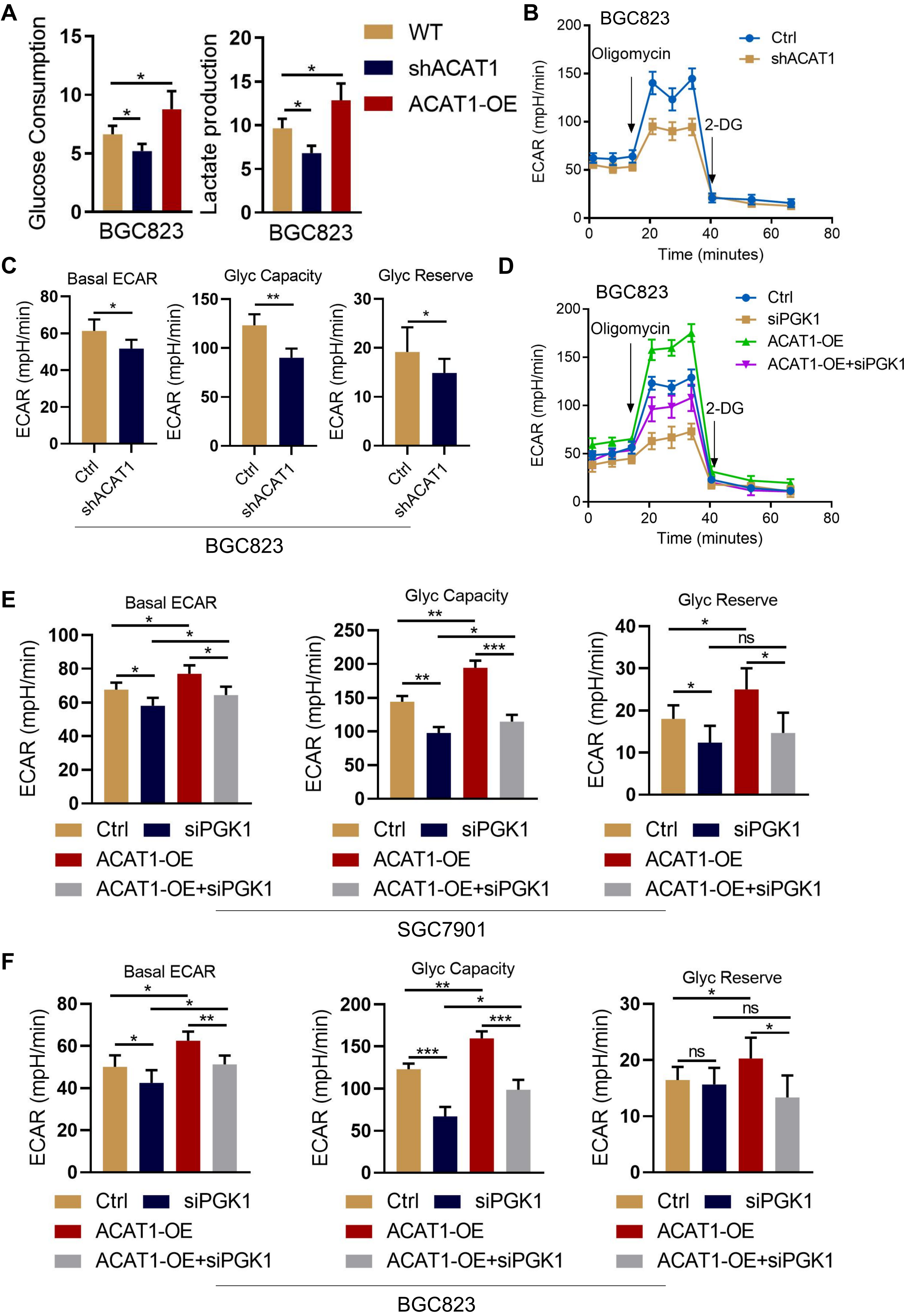

Supplement: Supplementary file 4 — Supplementary Figure 4 [file 41419_2025_8015_MOESM4_ESM.pdf]

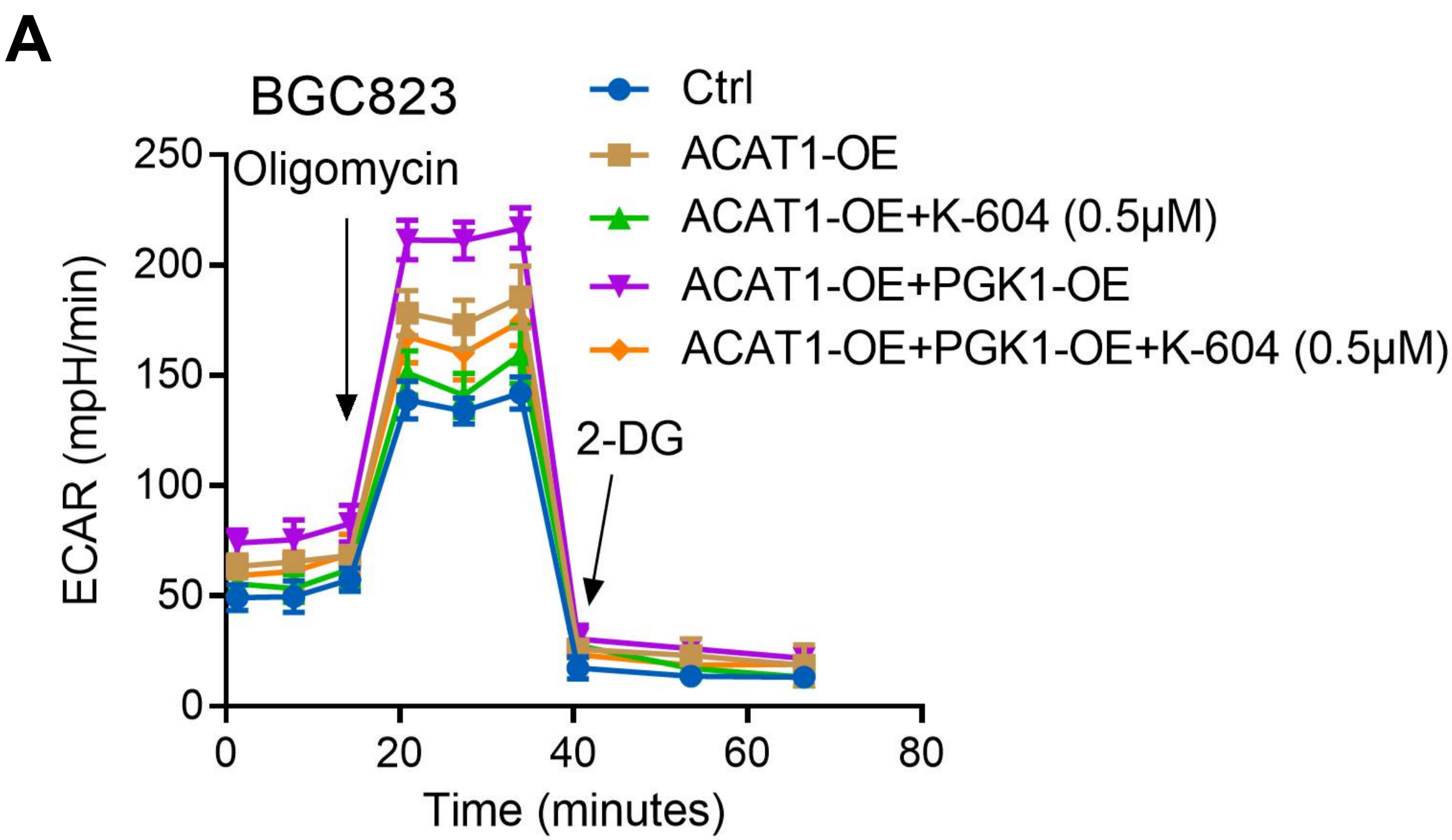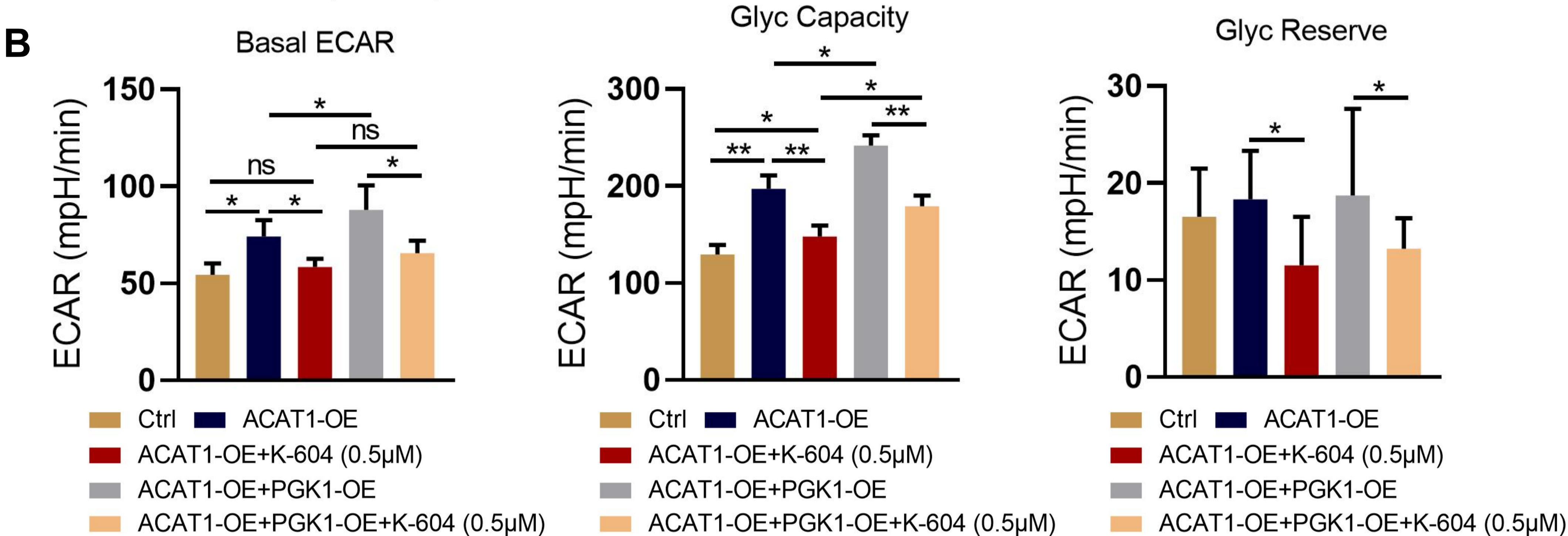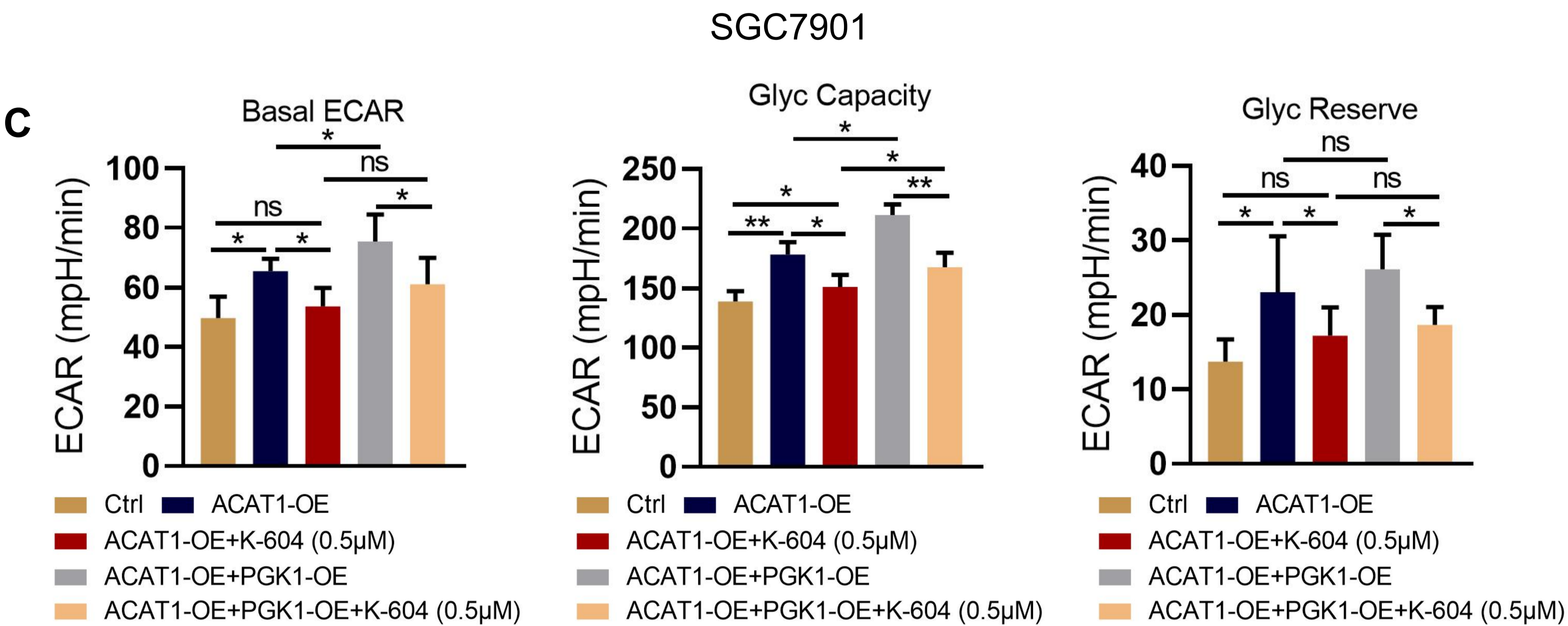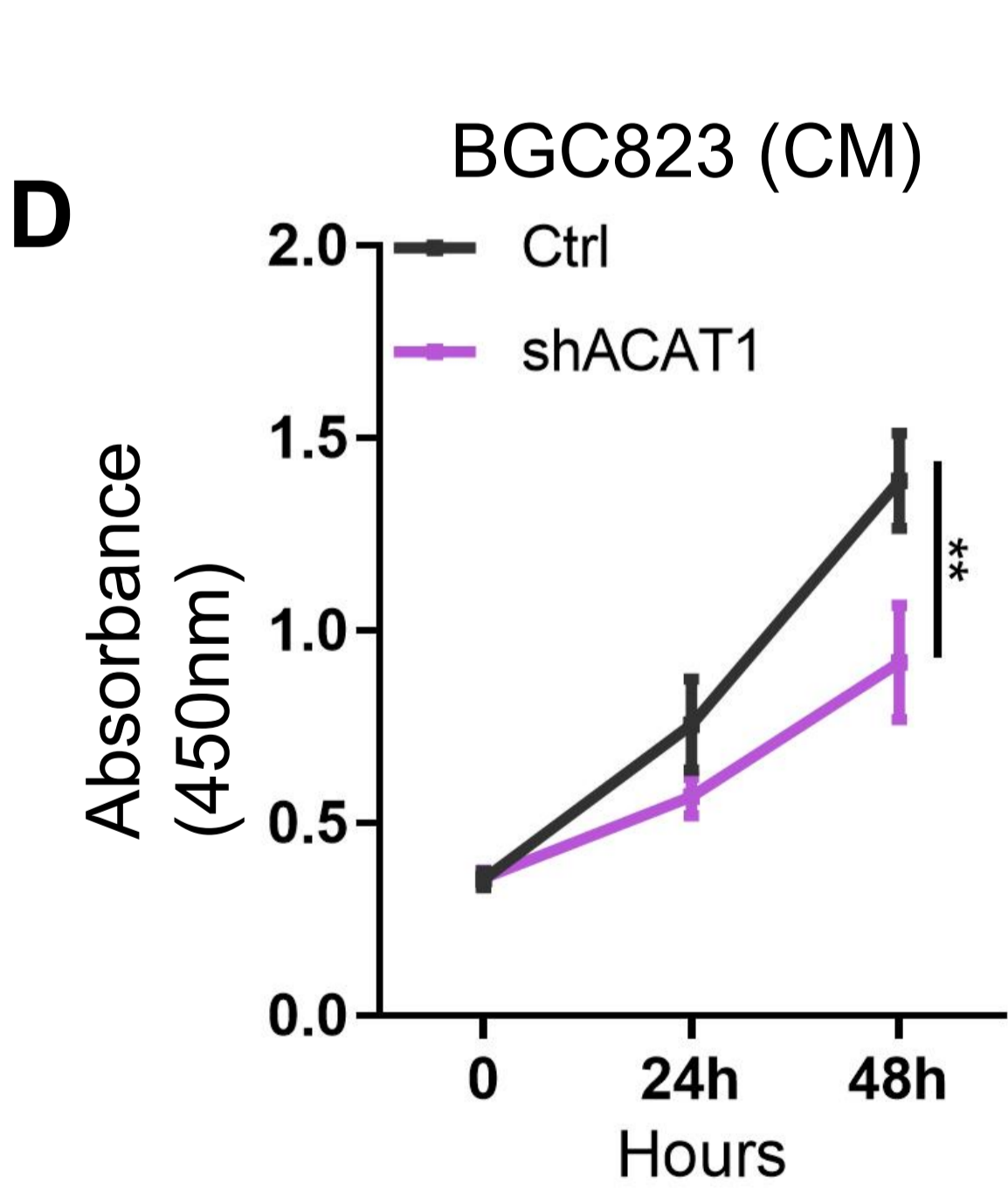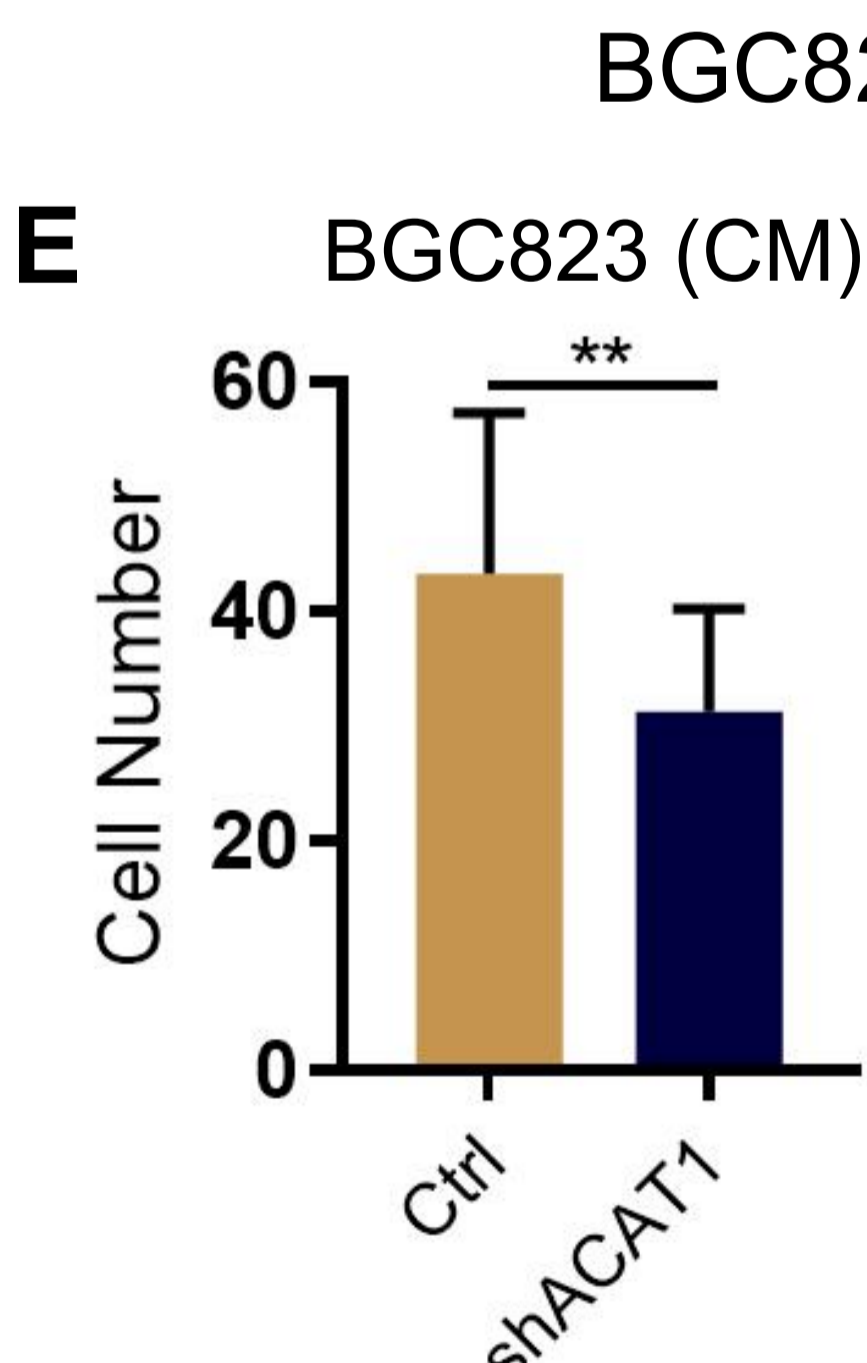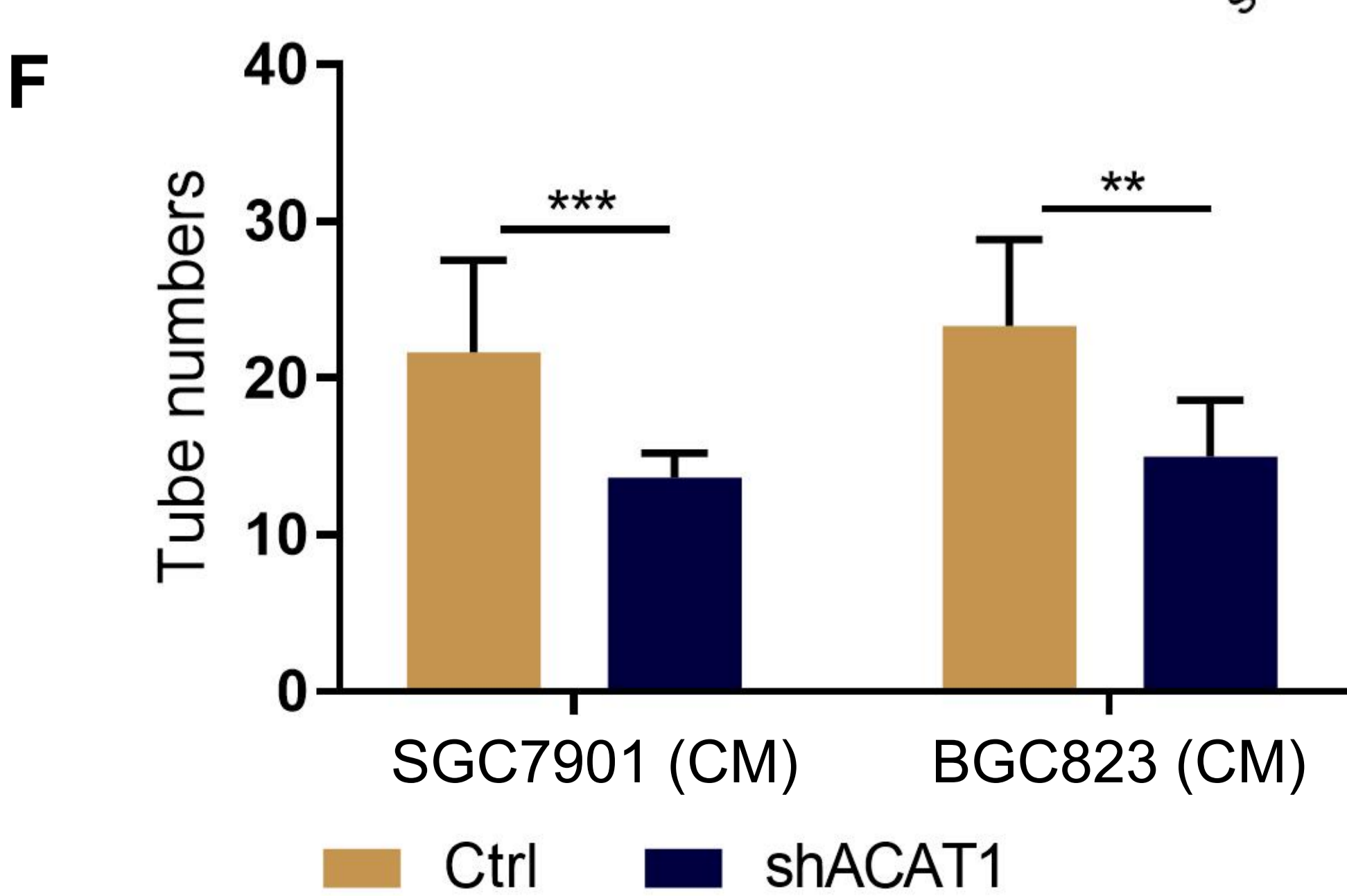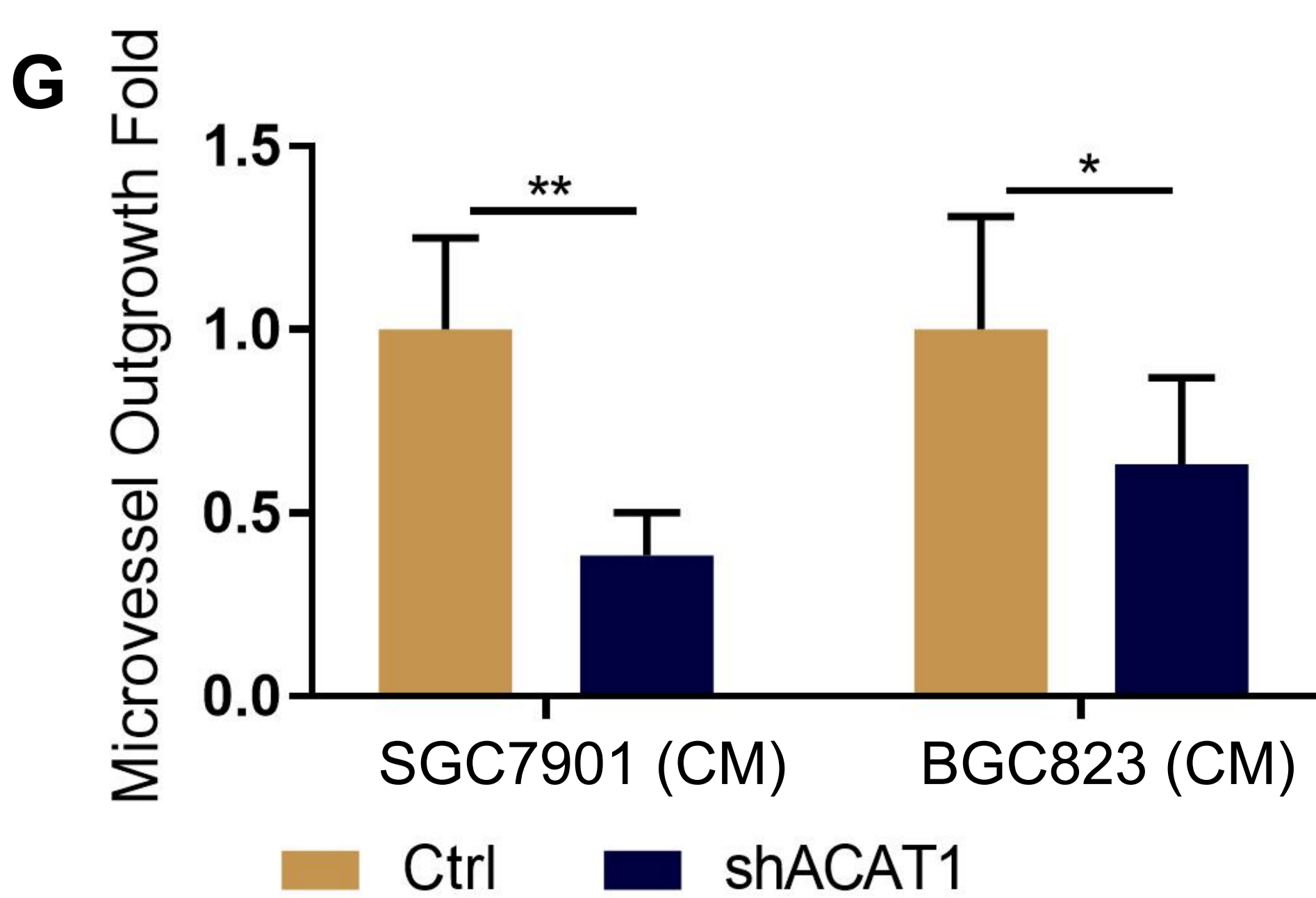

Supplement: Supplementary file 5 — Supplementary Figure 5 [file 41419_2025_8015_MOESM5_ESM.pdf]

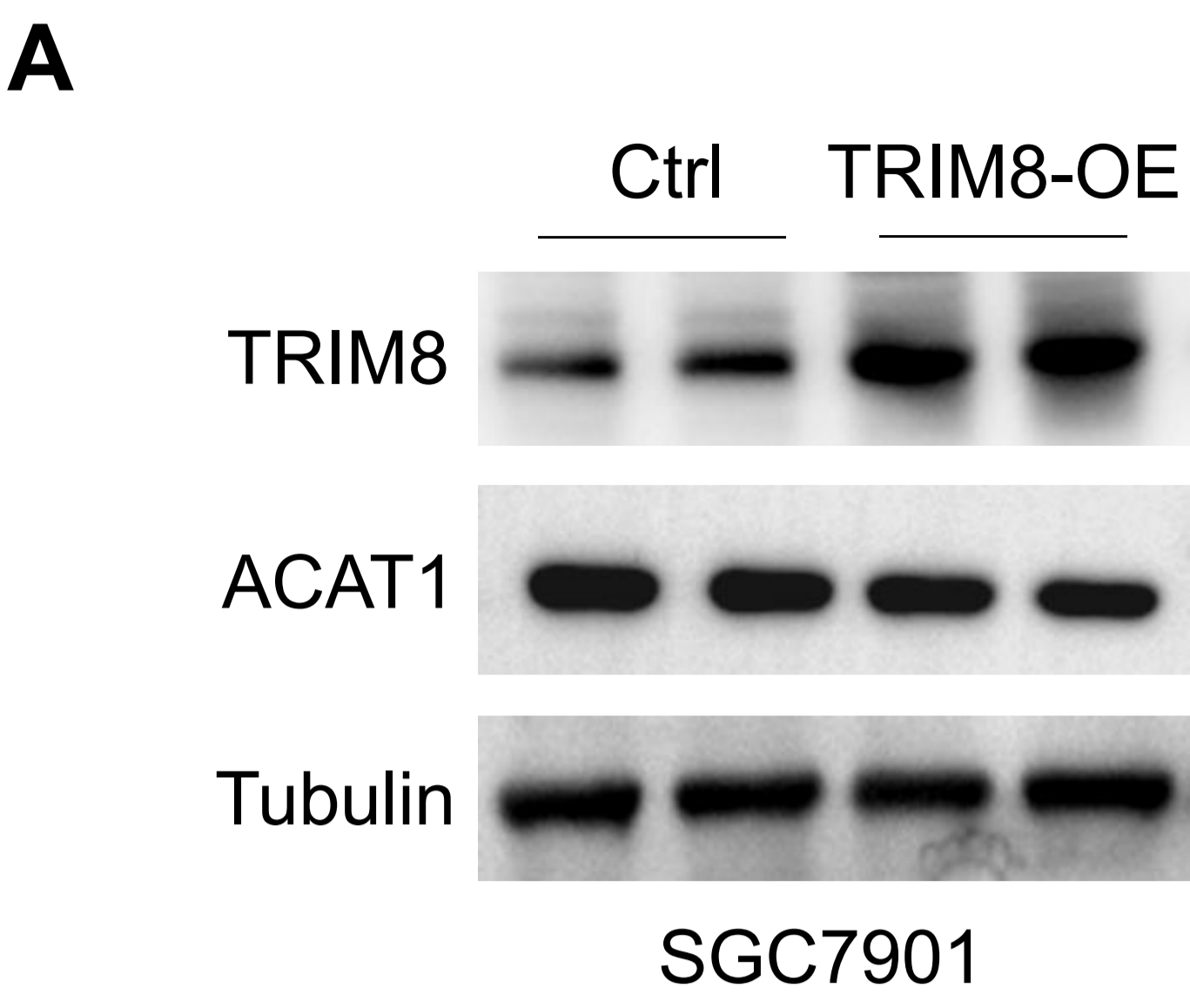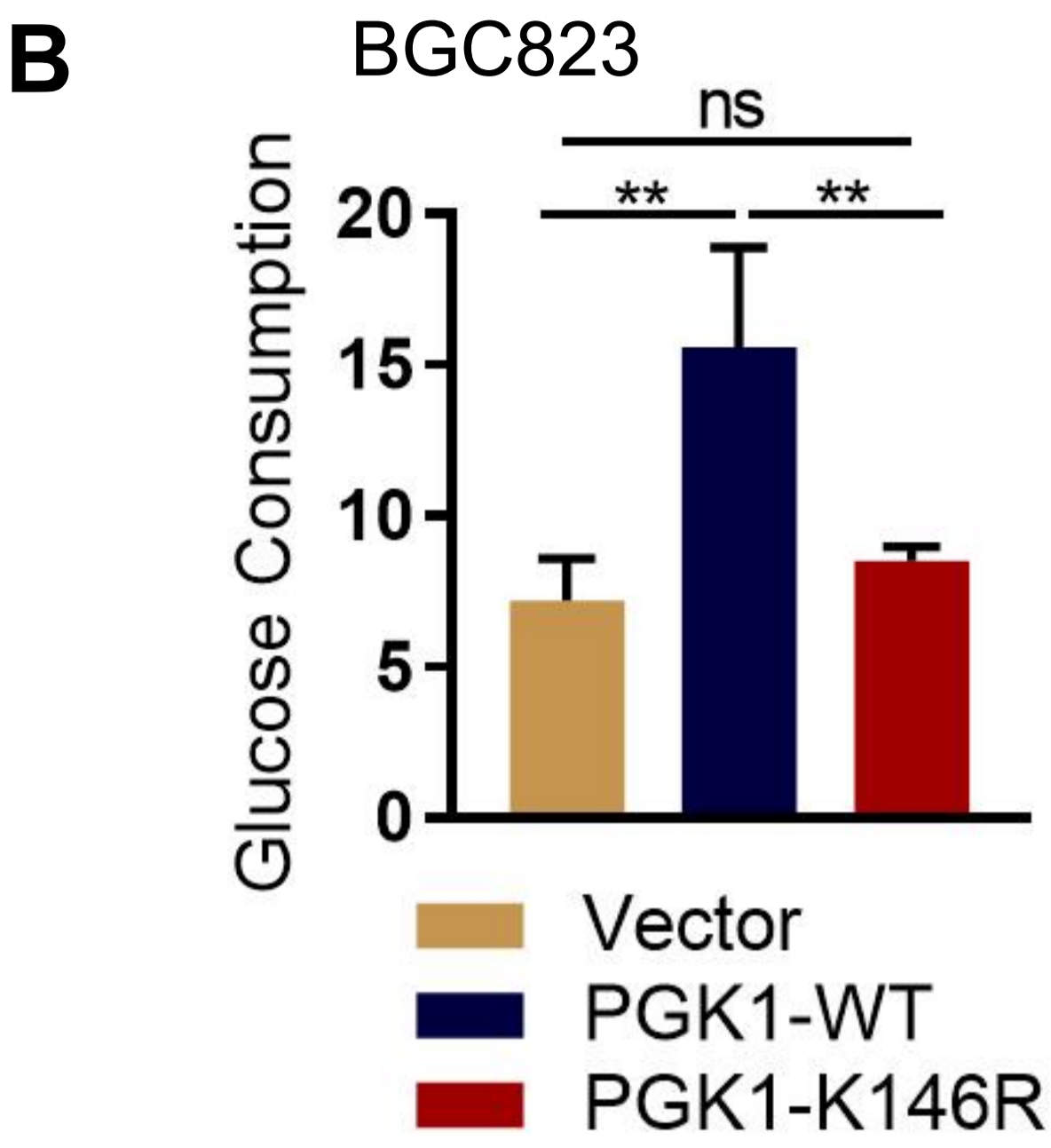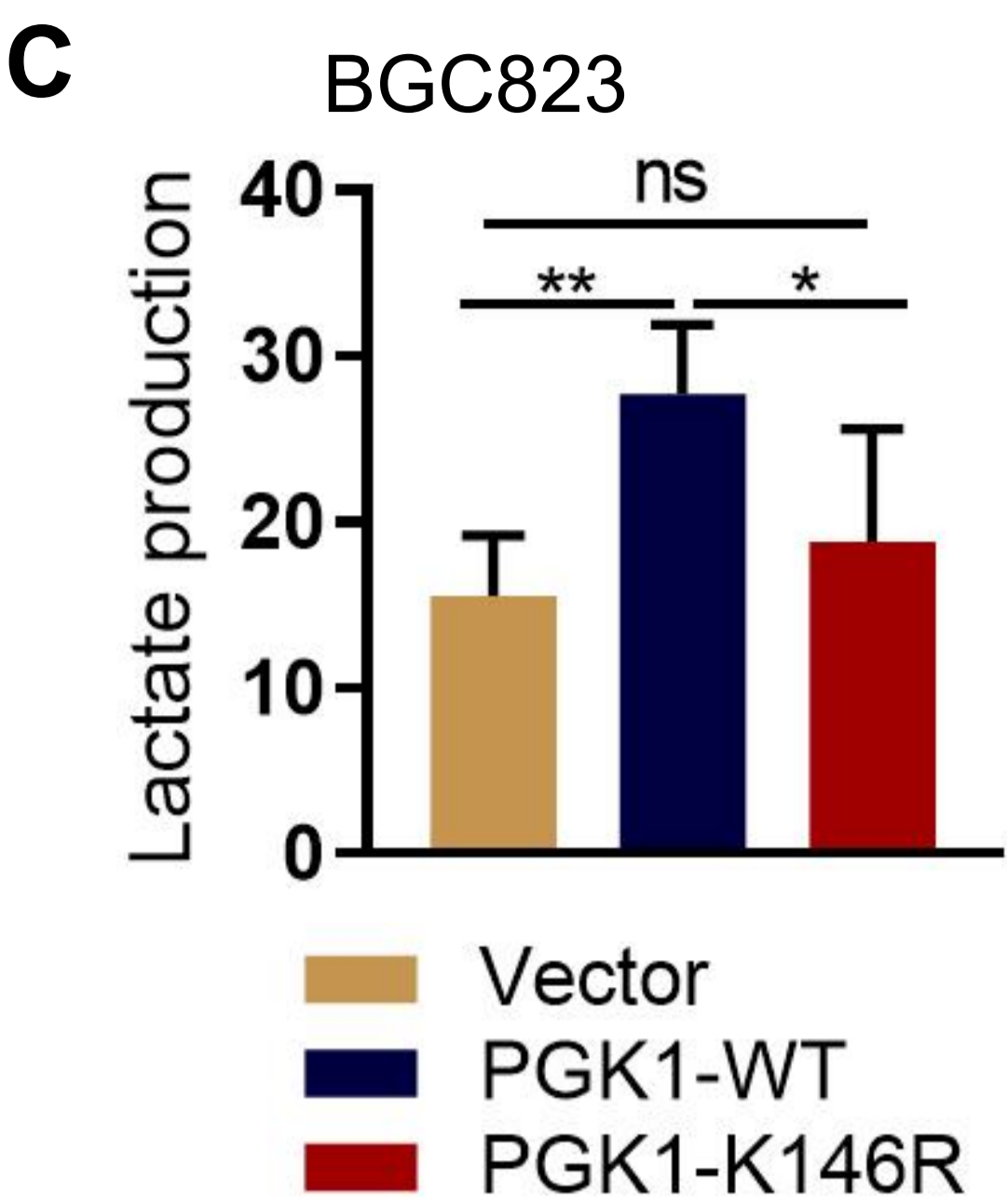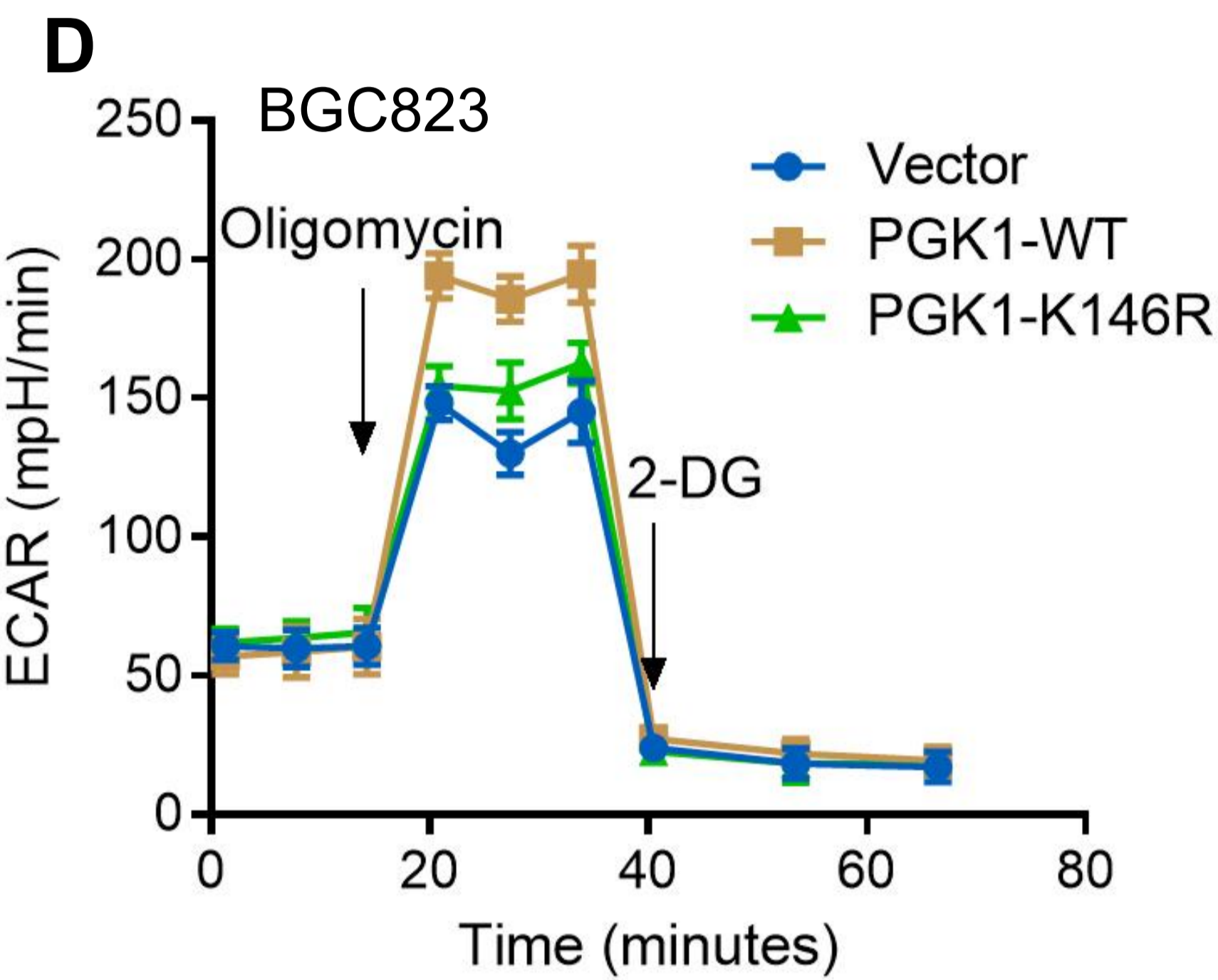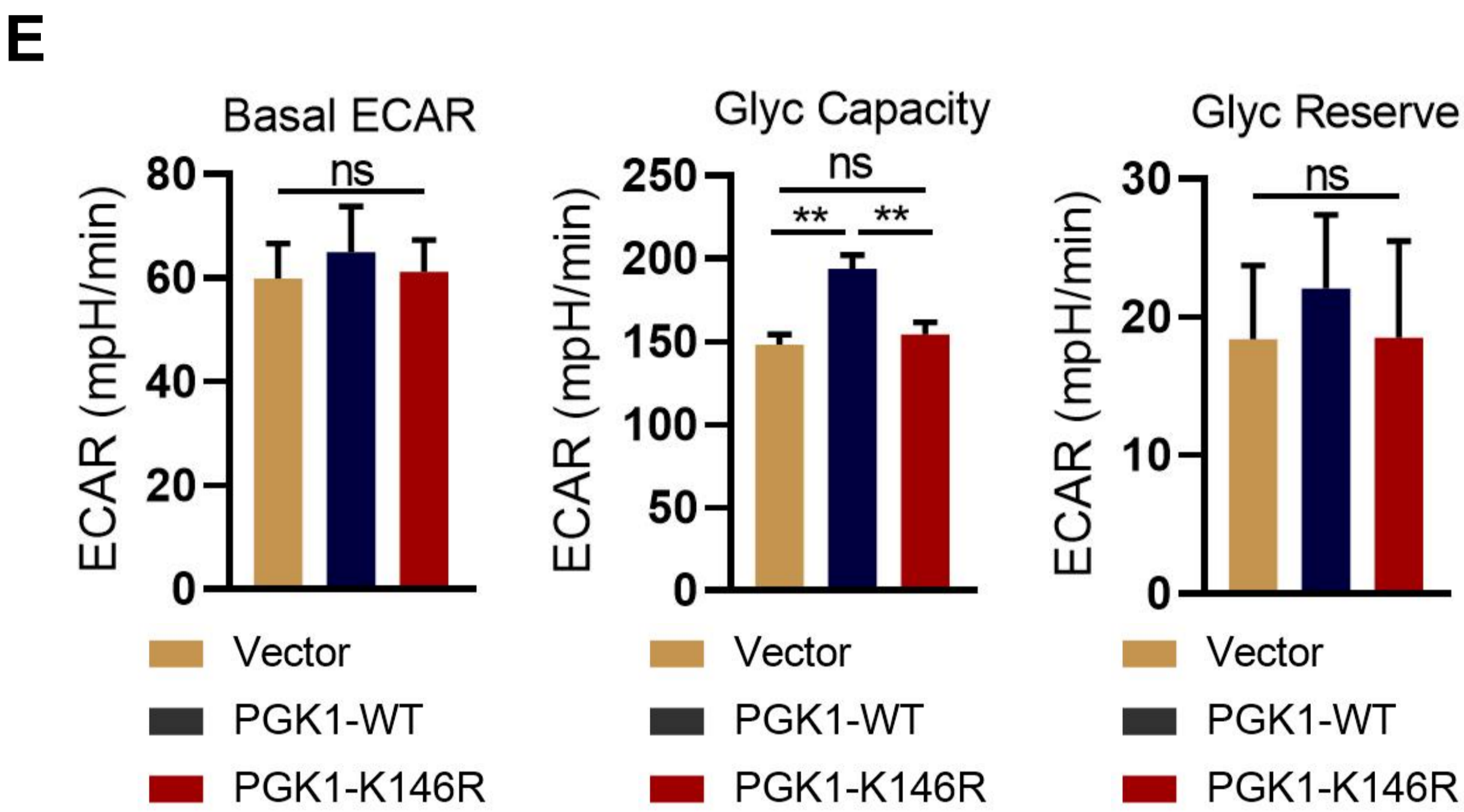

Supplement: Supplementary file 6 — Supplementary Figure 6 [file 41419_2025_8015_MOESM6_ESM.pdf]
