# Supplementary material for: TRIM8-dependent K63-ubiquitinated PGK1 promotes glycolysis and angiogenesis in gastric cancer via interaction with ACAT1
Source: Cell Death Dis. 2025 Nov 3;16(1):780. doi: 10.1038/s41419-025-08015-y (PMC12583530; doi:10.1038/s41419-025-08015-y)
Supplement: Supplementary file 7 — Supplementary Information [file 41419_2025_8015_MOESM7_ESM.docx]

**TRIM8-dependent** **K63-ubiquitinated PGK1 promotes glycolysis and angiogenesis in gastric cancer via interaction with ACAT1**

Anqi Feng^1*^, Jianbin Zhang^1*^, Zeyu Wang^1*^, Zhukai Chen^1^, Kang Fang^1^, Zhaoxing Li^1^, Hanyu Jiang^1^, Zhuyun Leng^1^, Shihan Zhang^1^, Yuan Chu^1^, Jingjing Lian^1^, Tao Chen^1,2,3^, Lechi Ye^4#^, Meidong Xu^1#^, Lingnan He^1,2,3#^

^1^Endoscopy Center, Department of Gastroenterology, Shanghai East Hospital, School of Medicine, Tongji University, Shanghai, China. Address: No.150, Jimo Road, Pudong New Area, Shanghai, China.

^2^School of Medicine, Tongji University, Shanghai, China. Address: No.500, Zhennan Road, Putuo District, Shanghai, China.

^3^Advanced Research Institute, Tongji University, Shanghai, China. Address: No.1239, Siping Road, Yangpu District, Shanghai, China.

^4^Department of Colorectal Surgery, Zhongshan Hospital, Fudan University, Shanghai, China. Address: No.180, Fenglin Road, Xuhui District, Shanghai, China.

**Supplementary Information**

**Supplementary Information 1:** **Supplementary Table 1.** Primers for qRT-PCR.

**Supplementary Information 2: Supplementary Table 2.** Mass spectrometric analysis analyzed the TRIM8-associated proteins.

**Supplementary Information 3: Supplementary Figure 1(Figure S1). related to Figure 1 and Figure 2.**

(A) The bar chart illustrates the expression fold changes of genes encoding E3 ubiquitin ligase in the TRIM family in TCGA dataset. (B) IHC staining with anti-TRIM8 or anti-PGK1 antibody was performed on human gastric cancer specimens. Representative photos of tumor tissues with low or high TRIM8 and PECAM1 expression from the same samples were shown (magnification: ×40 and ×200). (C) CCK-8 assay of GC cell lines transduced with shTRIM8 or control. *, p < 0.05, **, p < 0.01. (D) Colony formation assay of GC cell lines transduced with shTRIM8 or control. ***, p < 0.001.

**Supplementary Information 4: Supplementary Figure 2(Figure S2). TRIM8 promotes K63-linked ubiquitination of PGK1 and its stability, related to Figure 3**

(A) Immunoprecipitation assay was performed for the cytosol fraction of GC cell lines transduced with shTRIM8 or control. PGK1 IP on SGC7901 cells lysates transduced with shTRIM8 or control followed by Western blot with PGK1 and Ub antibodies. (B-C) In screening for potential lysine ubiquitination types, the ubiquitination of HA-PGK1 in response to TRIM8 overexpression was examined in GC cells transfected with the wild-type (WT) and Ub with the intact Lys48 or K63 residue alone or mutated His-Ub plasmids. K63, Ub with the intact Lys63 residue alone. K48, Ub with the intact Lys48 residue alone. K63R, Ub only Lys63 residue was mutated. (D) Representative WB analyses of PGK1 expression in TRIM8 over-expressed SGC7901 cells that were treated with the protein synthesis inhibitor cycloheximide (CHX; 50 µg/ml) for 8 h before extraction.

**Supplementary Information 5:** **Supplementary** **Figure 3 (Figure S3).** **K63- ubiquitination of PGK1 promotes glycolysis and angiogenesis in GC cells, related to Figure 4**

(A) BGC823 cells with or without TRIM8 depletion and TRIM8 overexpression were analyzed for glucose consumption and lactate production. (B-C) The glycolytic rate of cells in A was measured by Seahorse. ***, p < 0.001. ns, non-significant. (D) BGC823 cells stably overexpressing TRIM8 or control vector were transfected with control or PGK1 siRNA. CCK-8 assay of HUVECs co-cultured with BGC823 cells transduced with indicated vectors and plasmids. *, p < 0.05, **, p < 0.01. (E) BGC823 cells stably overexpressing TRIM8 or control vector were transfected with control or PGK1 siRNA. Transwell assay of HUVECs co-cultured with BGC823 cells transduced with indicated vectors and plasmids. Data shown is representative of 3 independent experiments. *, p < 0.05, **, p < 0.01. (F-G) BGC823 cells stably overexpressing TRIM8 or control vector were transfected with control or PGK1 siRNA. Tube formation of HUVECs co-cultured with BGC823 cells transduced with indicated vectors and plasmids was shown in representative images. Scale bar = 100μm. Magnification, 200×. *, p < 0.05, **, p < 0.01. (H-I) BGC823 Cells stably overexpressing PGK1 or control vector were transfected with control or TRIM8 shRNA. Cells were analyzed for glucose consumption(H) and lactate production(I). Data shown is representative of three independent experiments. (J) BGC823 Cells stably overexpressing PGK1 or control vector were transfected with control or TRIM8 shRNA. The glycolytic rate of cells was measured by Seahorse. Data shown is representative of three independent experiments. *, p < 0.05, **, p < 0.01. ***, p < 0.001. ns, non-significant.

**Supplementary Information 6: Supplementary Figure 4 (Figure S4). ACAT1-mediated PGK1 acetylation promotes PGK1 activity, GC cell glycolysis and tumor angiogenesis, related to Figure 5**

(A) BGC823 cells with or without ACAT1 depletion and ACAT1 overexpression were analyzed for glucose consumption and lactate production. *, p < 0.05 (B) BGC823 cells with control or ACAT1 depletion. The glycolytic rate of cells was measured by Seahorse. (C) Quantification of B. *, p < 0.05, **, p < 0.01. (D) The glycolytic rate of BGC823 cells transfected with indicated plasmids was measured by Seahorse. (E) Quantification of Figure 5G. *, p < 0.05, **, p < 0.01. ***, p < 0.001. ns, non-significant. (F) Quantification of D. *, p < 0.05, **, p < 0.01. ***, p < 0.001. ns, non-significant.

**Supplementary Information 7:** **Supplementary Figure 5 (Figure S5).** **ACAT1 -mediated PGK1 acetylation promotes PGK1 activity, GC cell glycolysis and tumor angiogenesis, related to Figure 5**

(A)The glycolytic rate of BGC823 cells transfected with indicated plasmids was measured by Seahorse. Cells were pretreated with or without K-604(0.5μM), a potent and selective ACAT1 enzyme activity inhibitor. (B) Quantification of Figure 5H. *, p < 0.05, **, p < 0.01. ns, non-significant. (C) Quantification of A. *, p < 0.05, **, p < 0.01. ns, non-significant.

(D) CCK-8 assay of HUVECs co-cultured with BGC823 cells transduced with control or ACAT1 shRNA. (E) Transwell assay on HUVECs co-cultured with BGC823 cells transfected with control or ACAT1 shRNA. (F) Quantification of Figure 5K. **, p < 0.01. ***, p < 0.001. (G) Quantification of Figure 5L. *, p < 0.05. **, p < 0.01.

**Supplementary Information 8:** **Supplementary Figure 6 (Figure S6).** **ACAT1 recruitment and PGK1 acetylation are dependent on TRIM8-mediated PGK1 K63-linked ubiquitination, related to Figure 6**

(A) Western blot of ACAT1 and TRIM8 in SGC7901 cells stably overexpressing TRIM8 or control vector. (B-C) BGC823 cells transfected with PGK1 WT or K146R were analyzed for glucose consumption(B) and lactate production (C). (D) The glycolytic rate of cells in B was measured by Seahorse. (E) Quantification of D. **, p < 0.01. ns, non-significant.

**Supplementary Information 9: Uncropped original Western blots**.
