# Supplementary material for: TRIM8-dependent K63-ubiquitinated PGK1 promotes glycolysis and angiogenesis in gastric cancer via interaction with ACAT1
Source: Cell Death Dis. 2025 Nov 3;16(1):780. doi: 10.1038/s41419-025-08015-y (PMC12583530; doi:10.1038/s41419-025-08015-y)
Supplement: Supplementary file 8 — Supplementary Table 1 [file 41419_2025_8015_MOESM8_ESM.docx]

Supplementary Table 1. Primer for qRT-PCR

| **Number** | **Primer for qRT-PCR (human)** | **Forward primer** | **Reverse primer** |
| --- | --- | --- | --- |
| 1 | β-Actin | CACTCTTCCAGCCTTCCTTC | GTACAGGTCTTTGCGGATGT |
| 2 | TRIM8 | TCTGTGGACAACTGTTACTGTTCTTC | GCGGGAGCGGGTGTGAG |
| 3 | PGK1 | GAGCCCAGAGCGACCCTTC | AGCCATTCCACCACCAATAATCATC |
| 4 | ACAT1 | CTGACGCTGCTGTAGAACCTATTG | GGCTTCATTTACTTCCCACATTGC |
