# Supplementary material for: TRIM8-dependent K63-ubiquitinated PGK1 promotes glycolysis and angiogenesis in gastric cancer via interaction with ACAT1
Source: Cell Death Dis. 2025 Nov 3;16(1):780. doi: 10.1038/s41419-025-08015-y (PMC12583530; doi:10.1038/s41419-025-08015-y)

Figure 1L

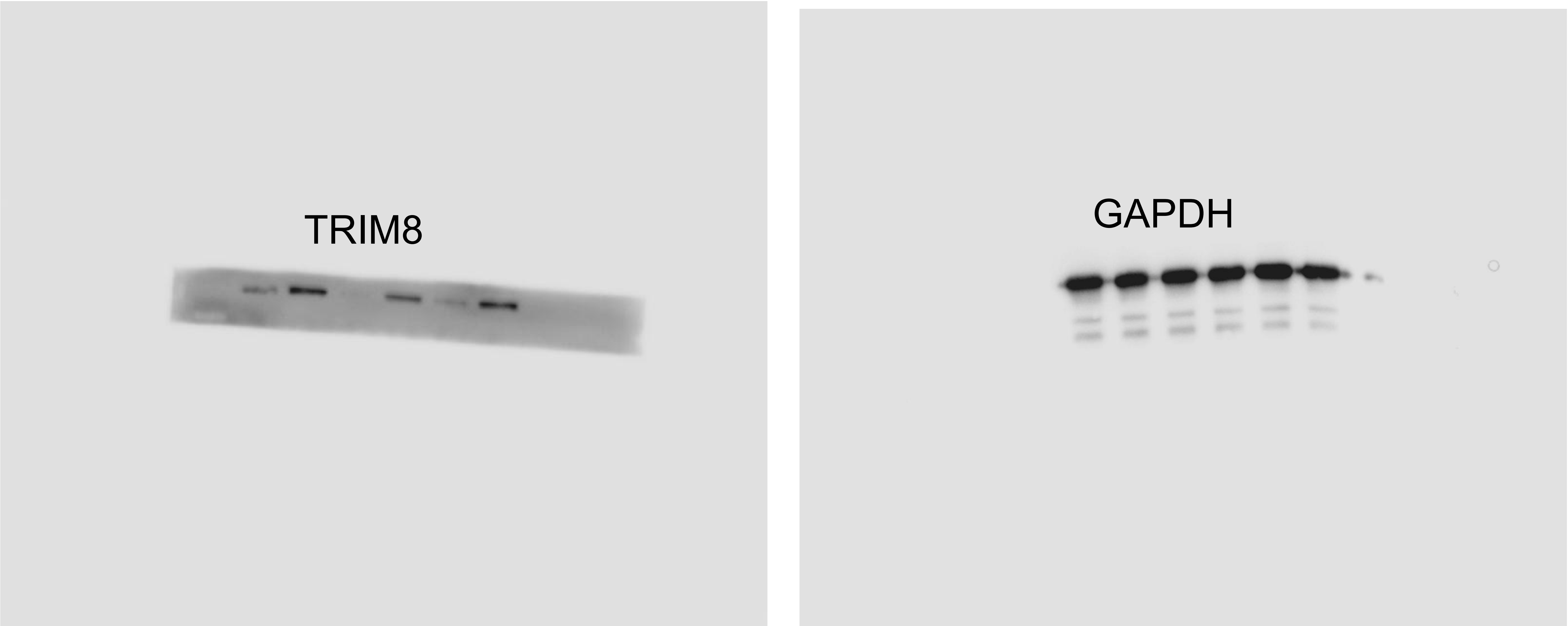

Figure 3B

SGC7901

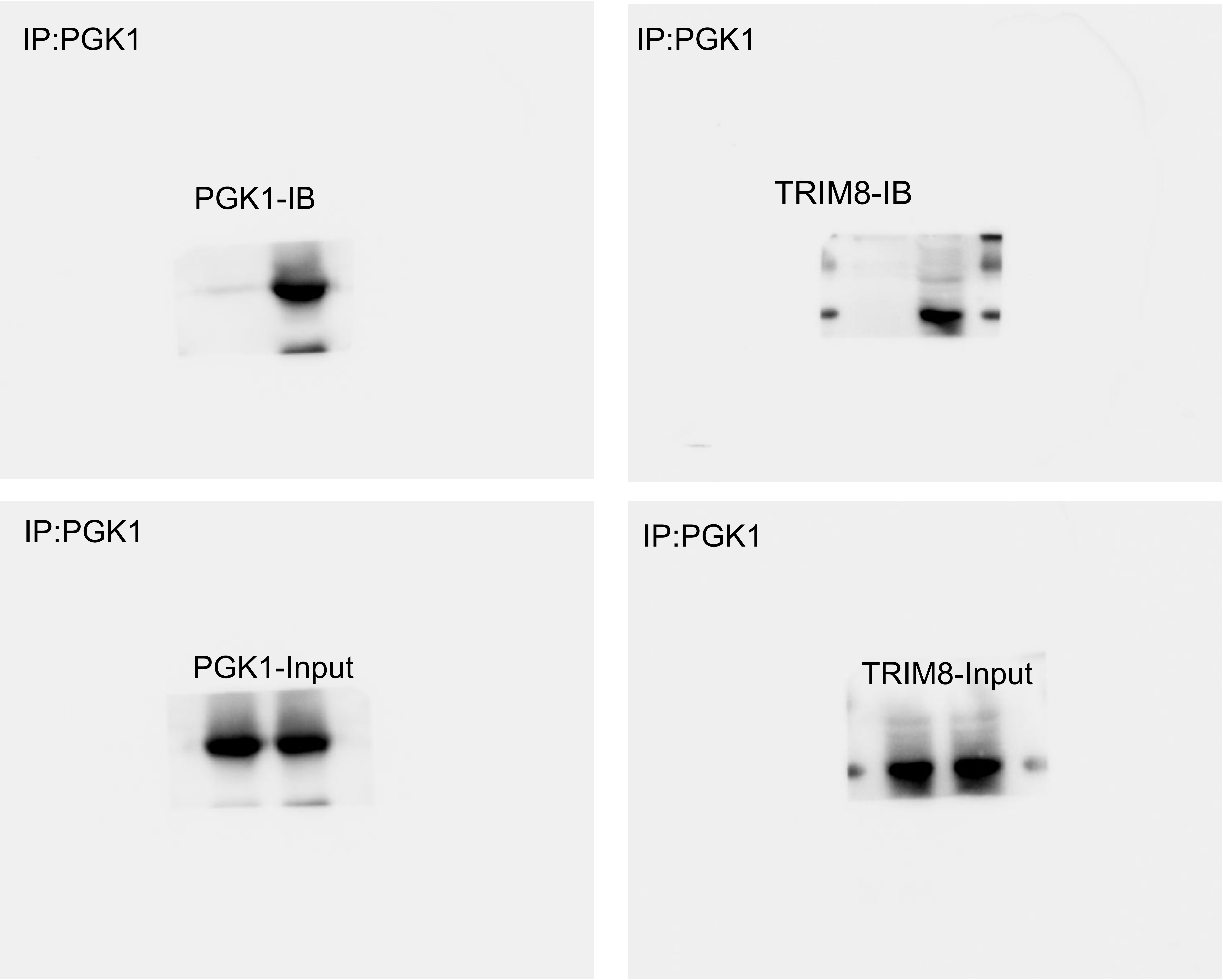

Figure 3B

SGC7901

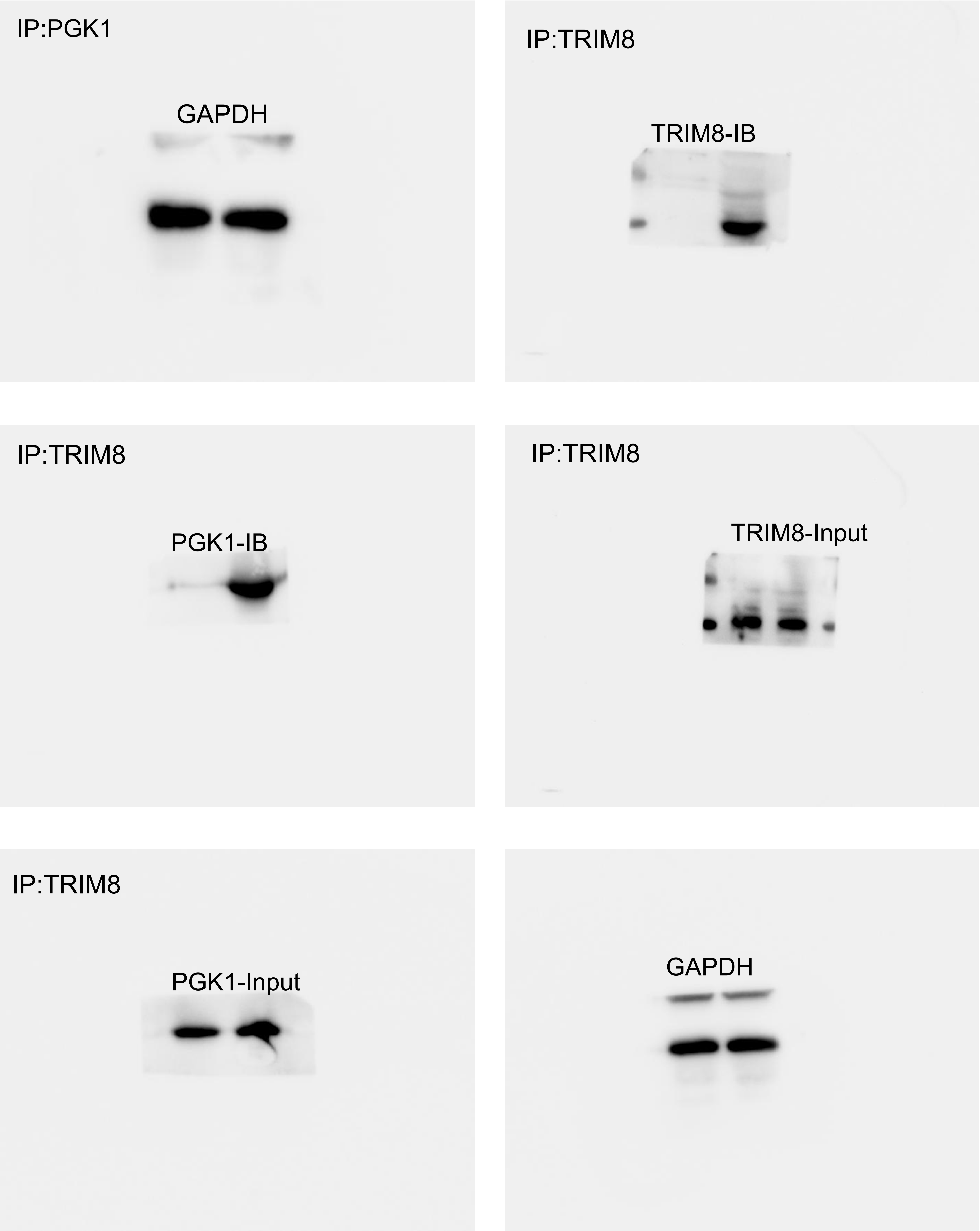

Figure 3B

BGC823

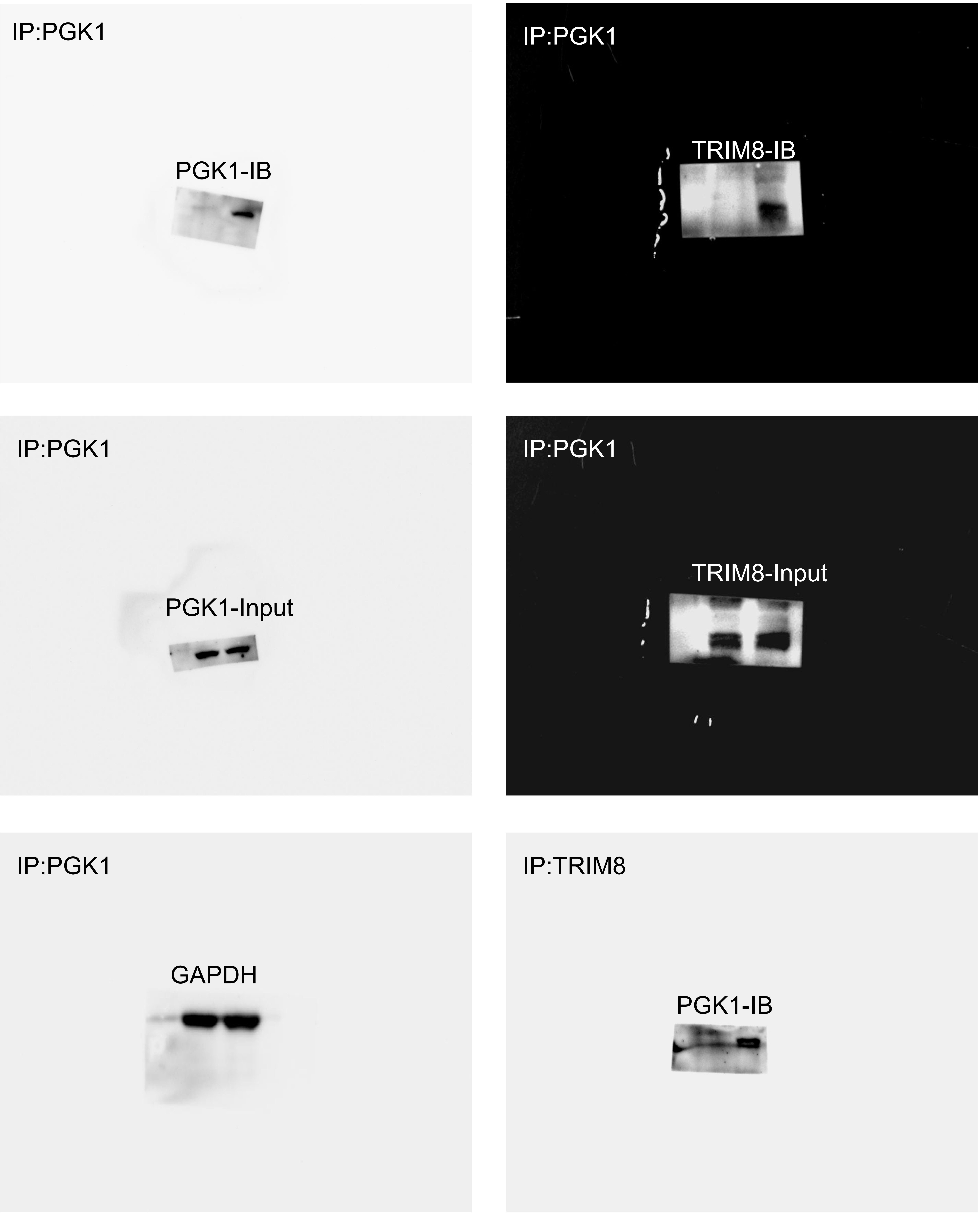

Figure 3B

BGC823

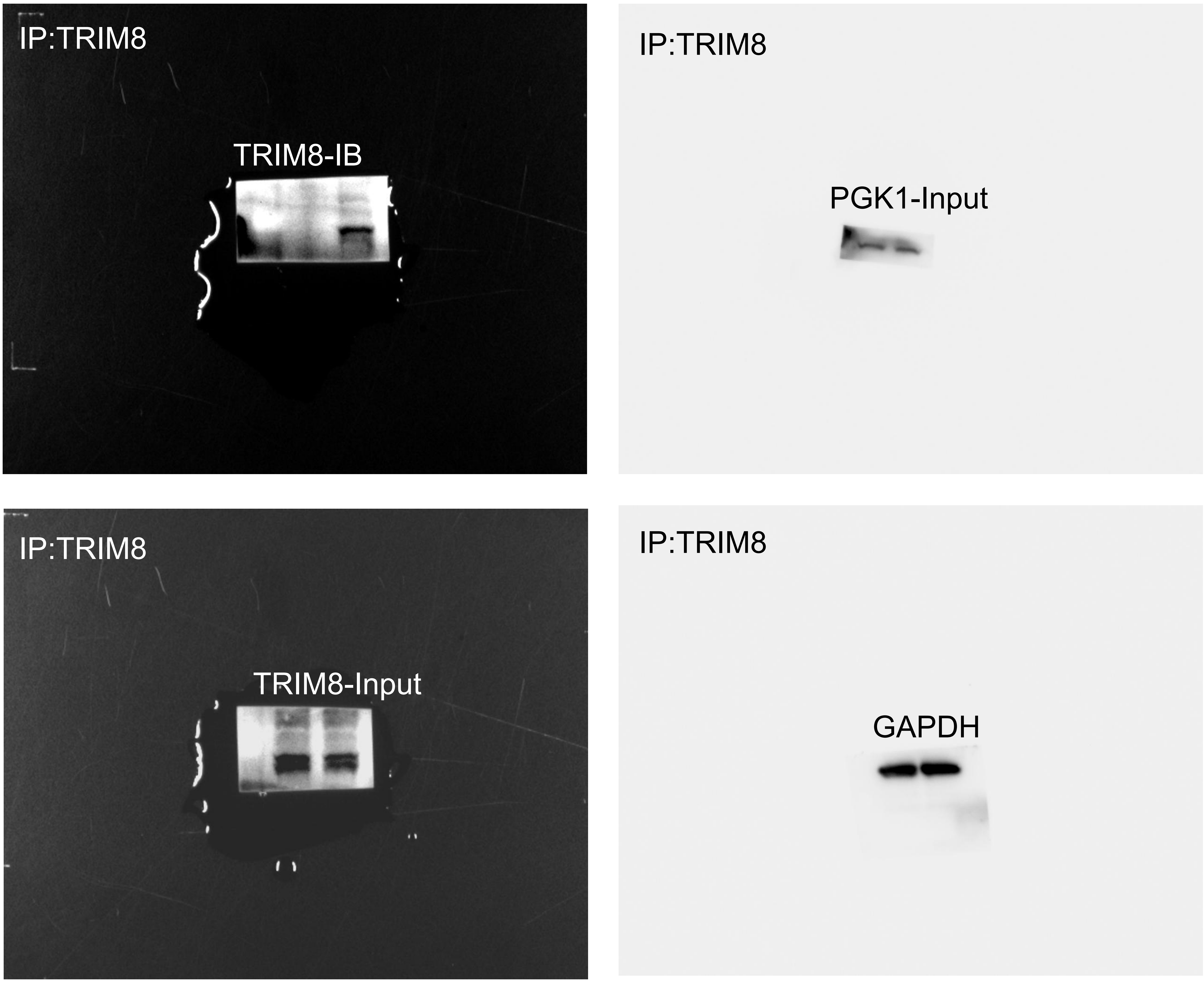

Figure 3C

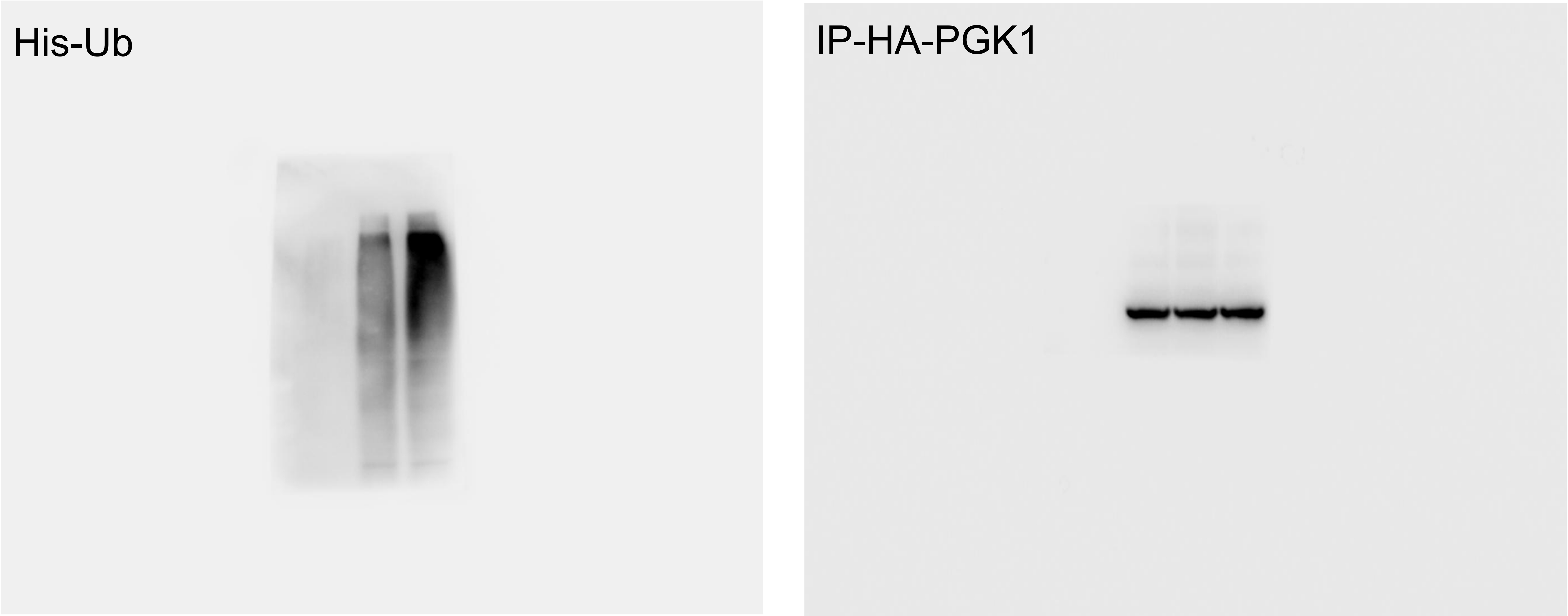

Figure 3C

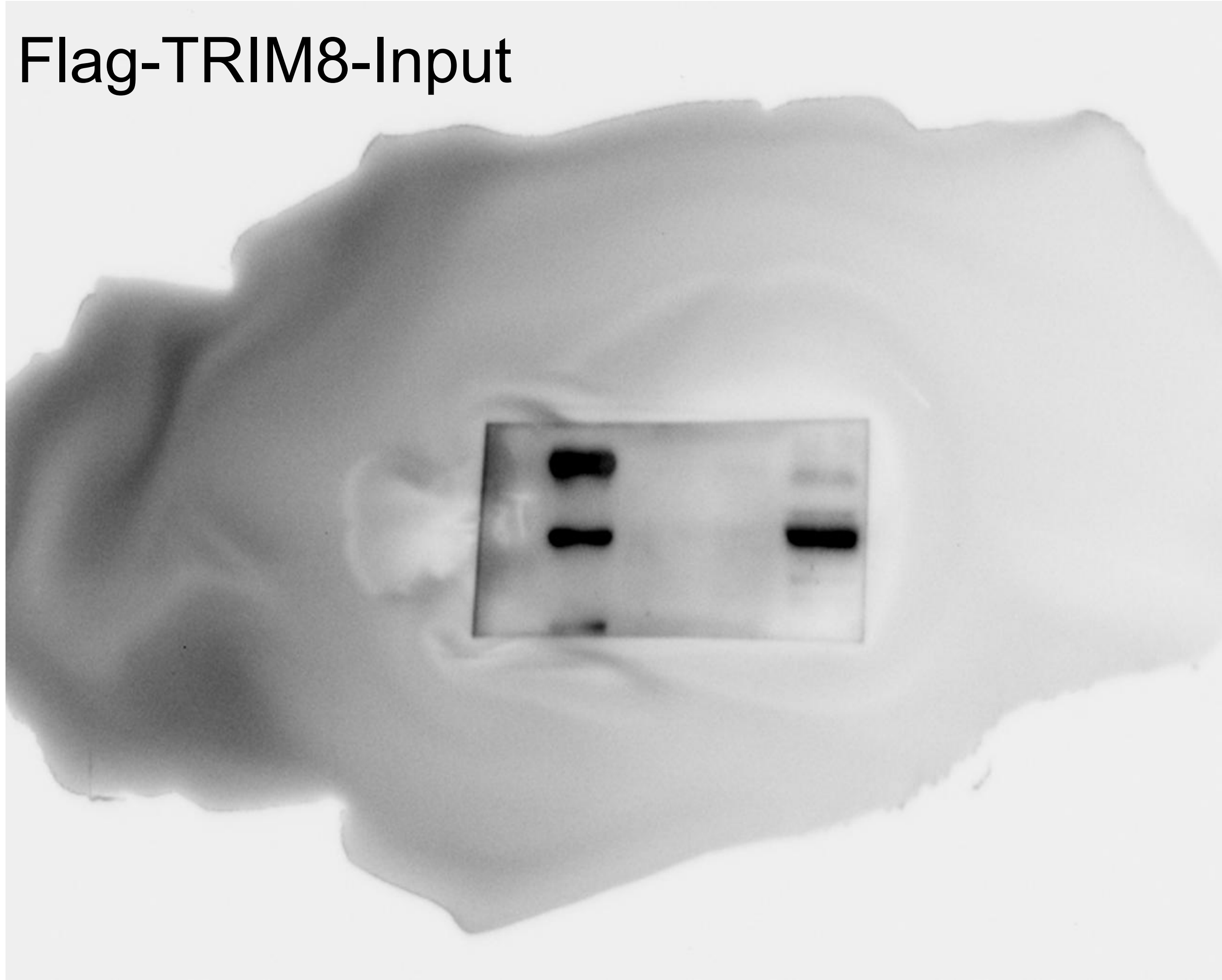

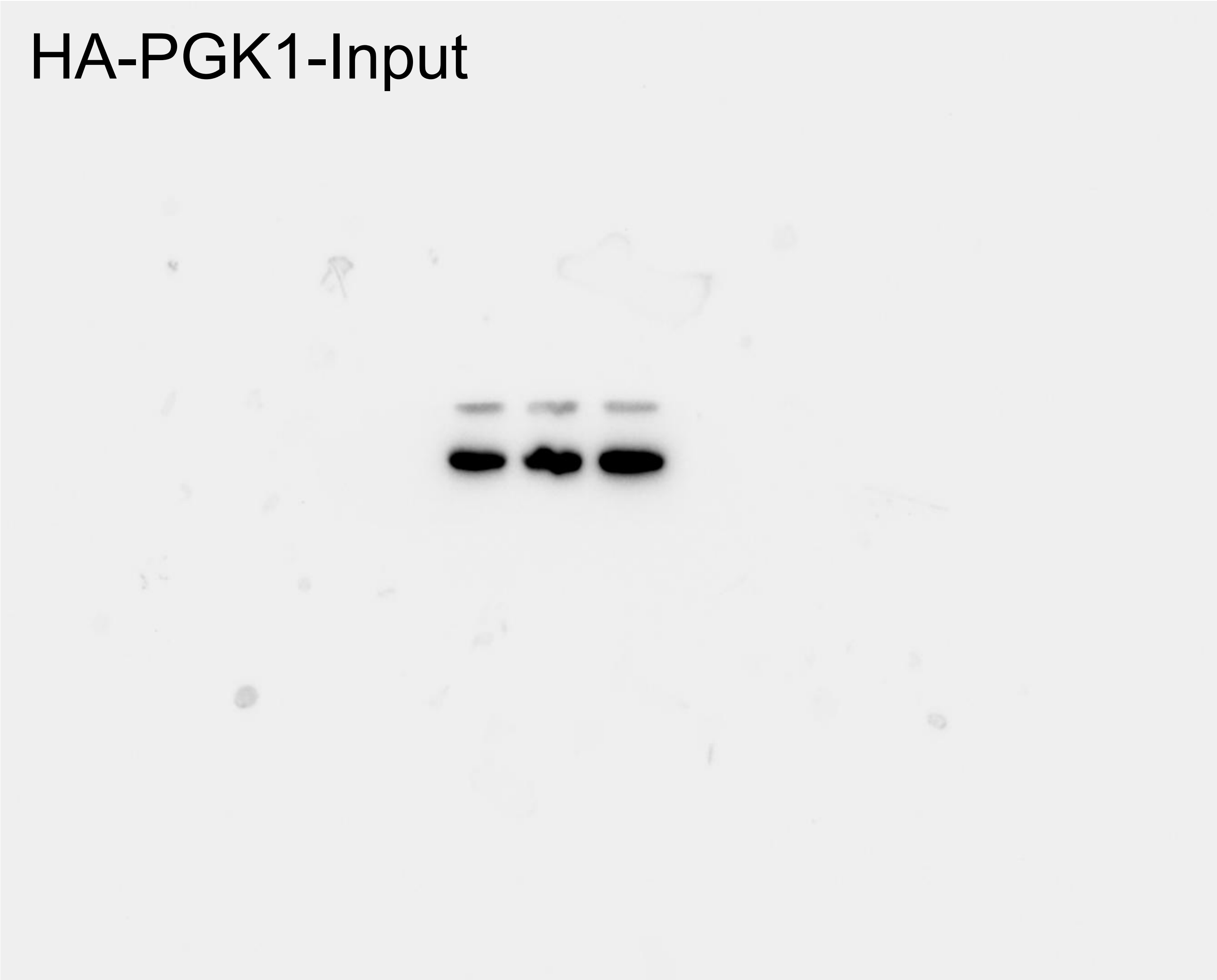

Figure 3D

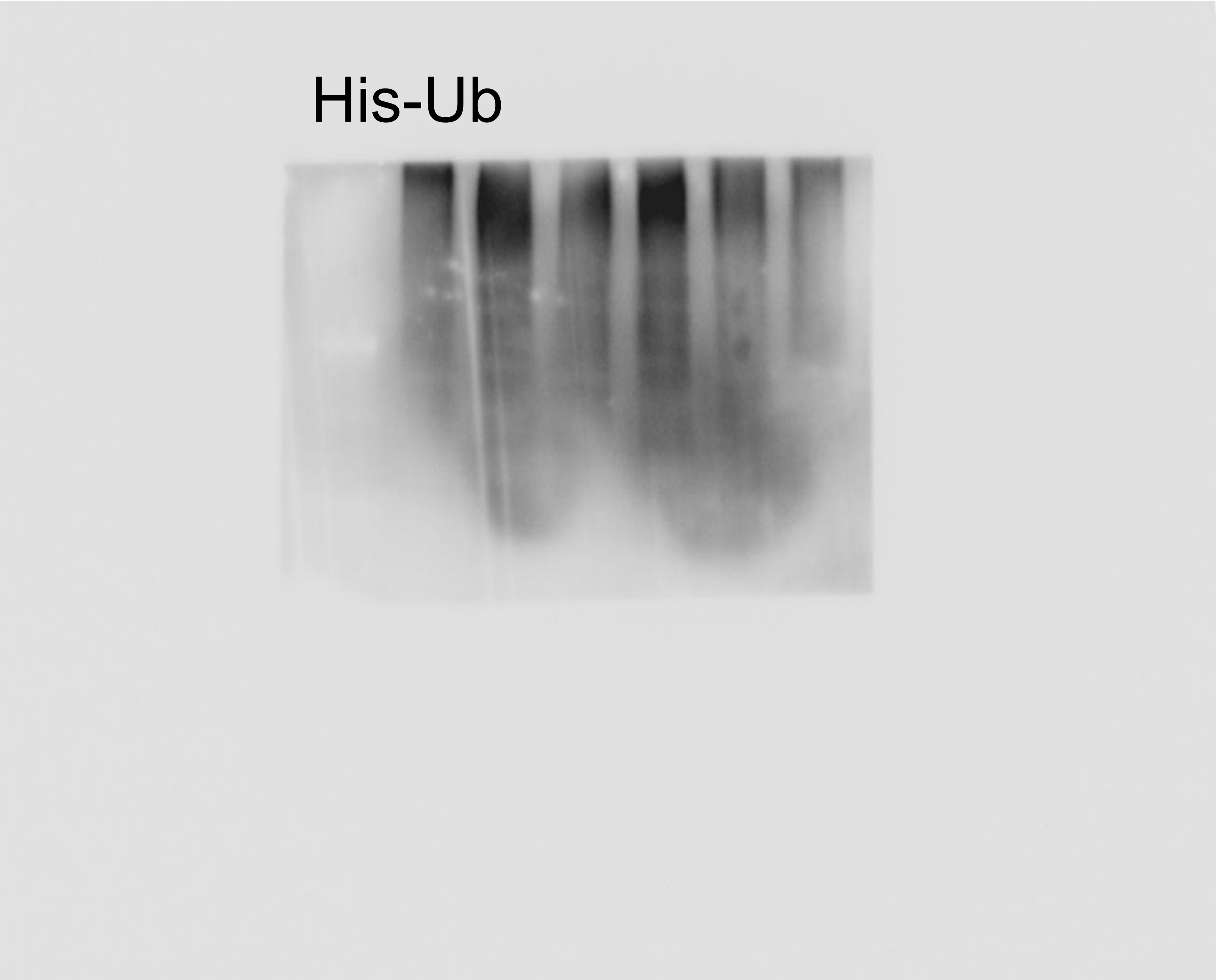

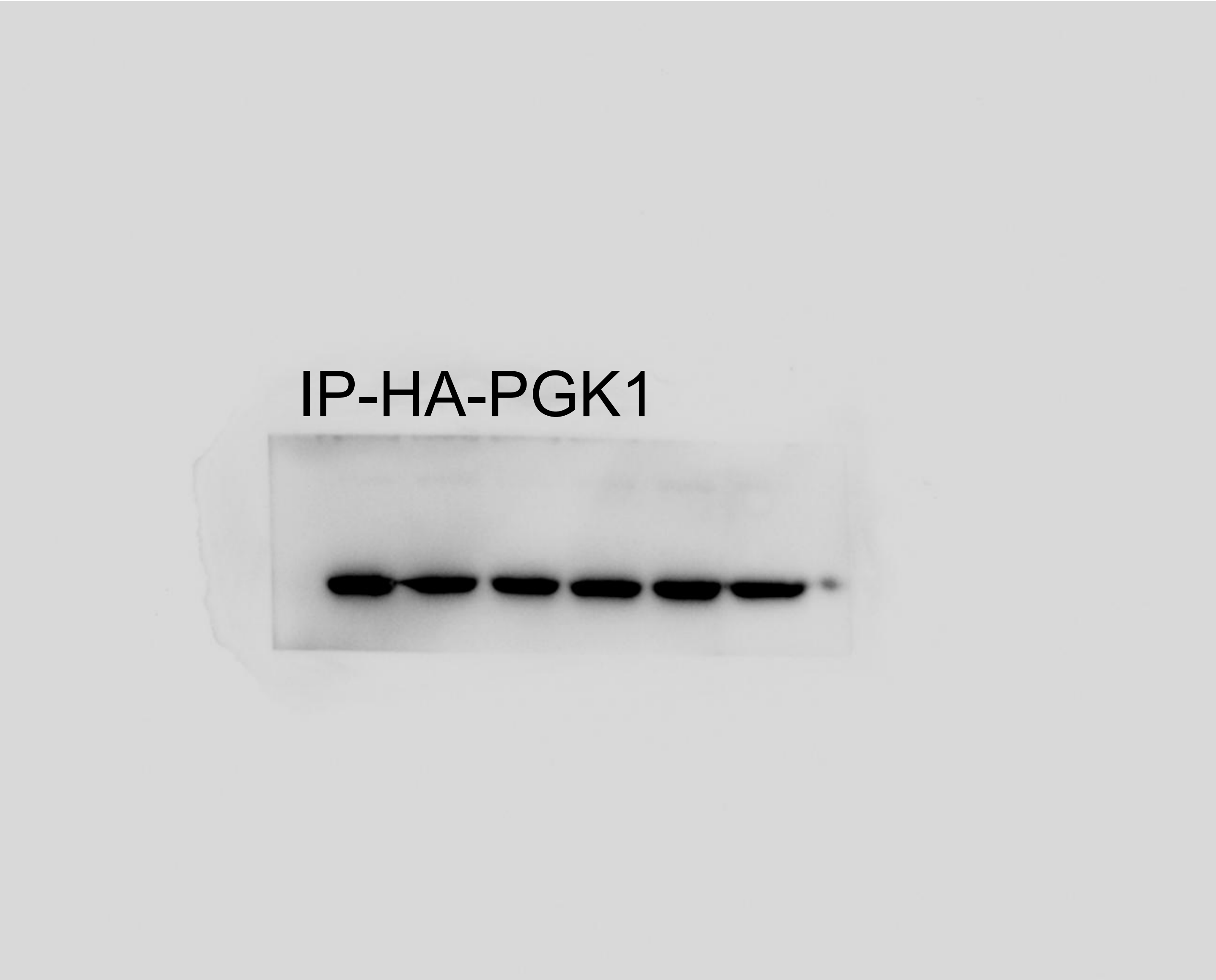

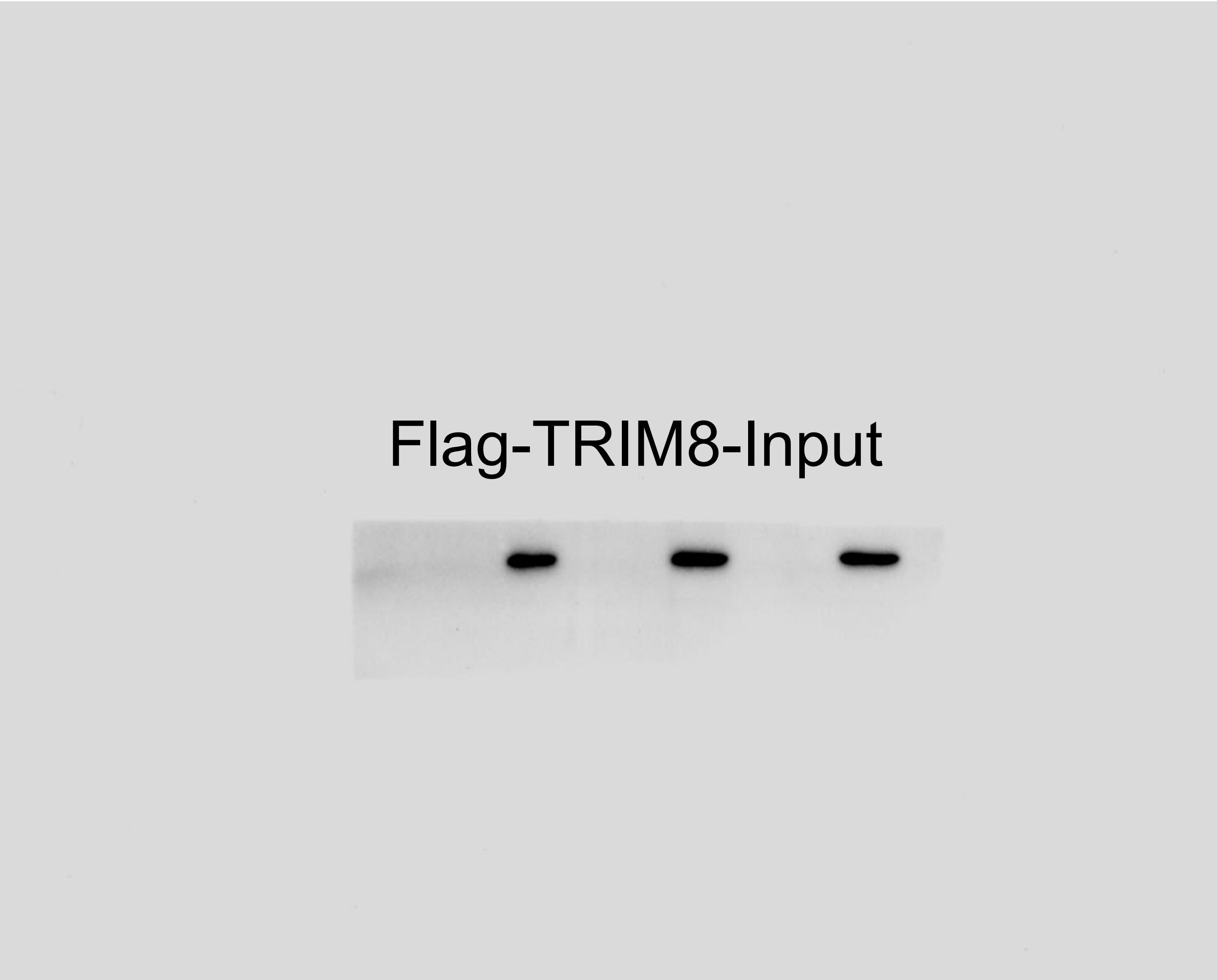

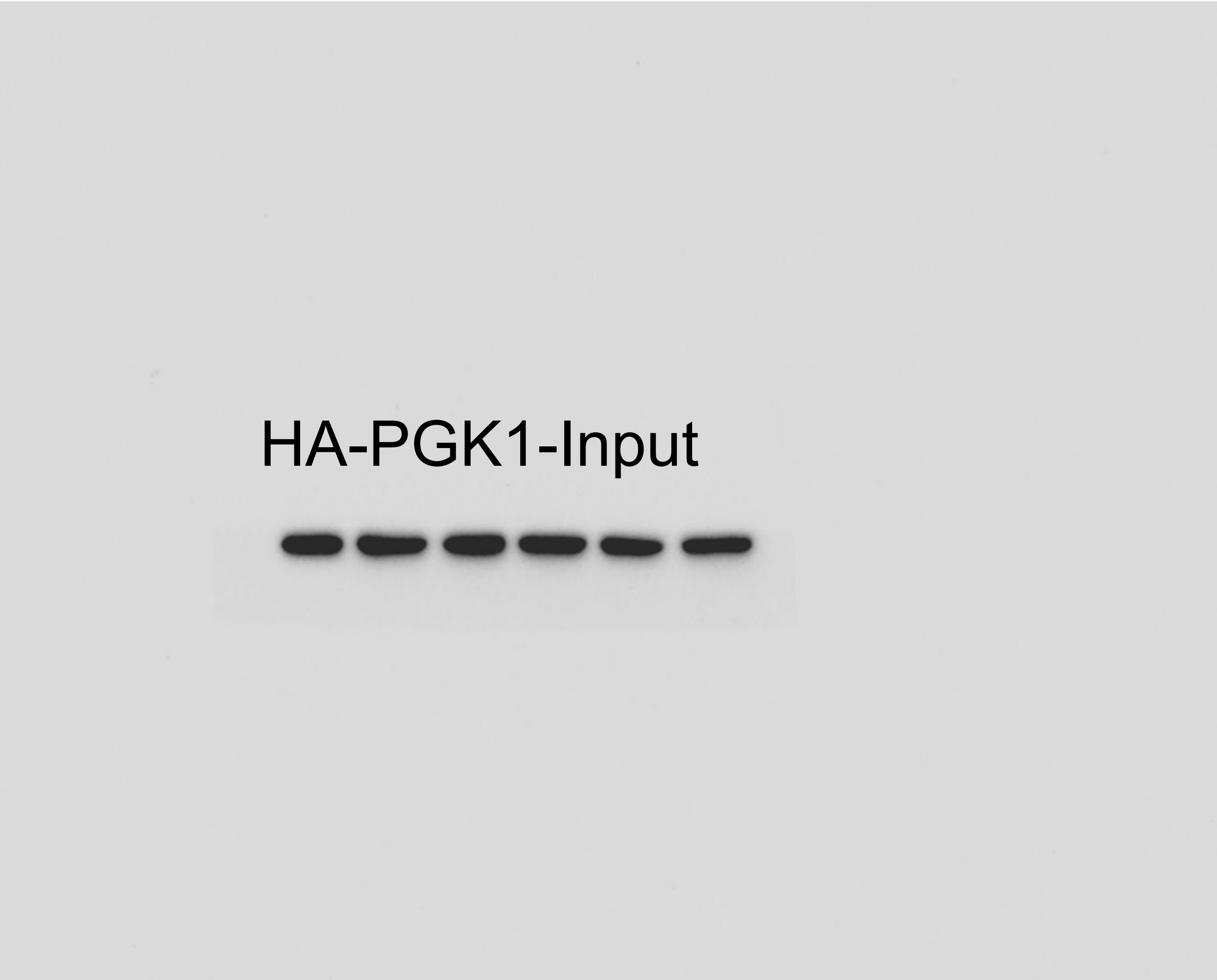

Figure 3E

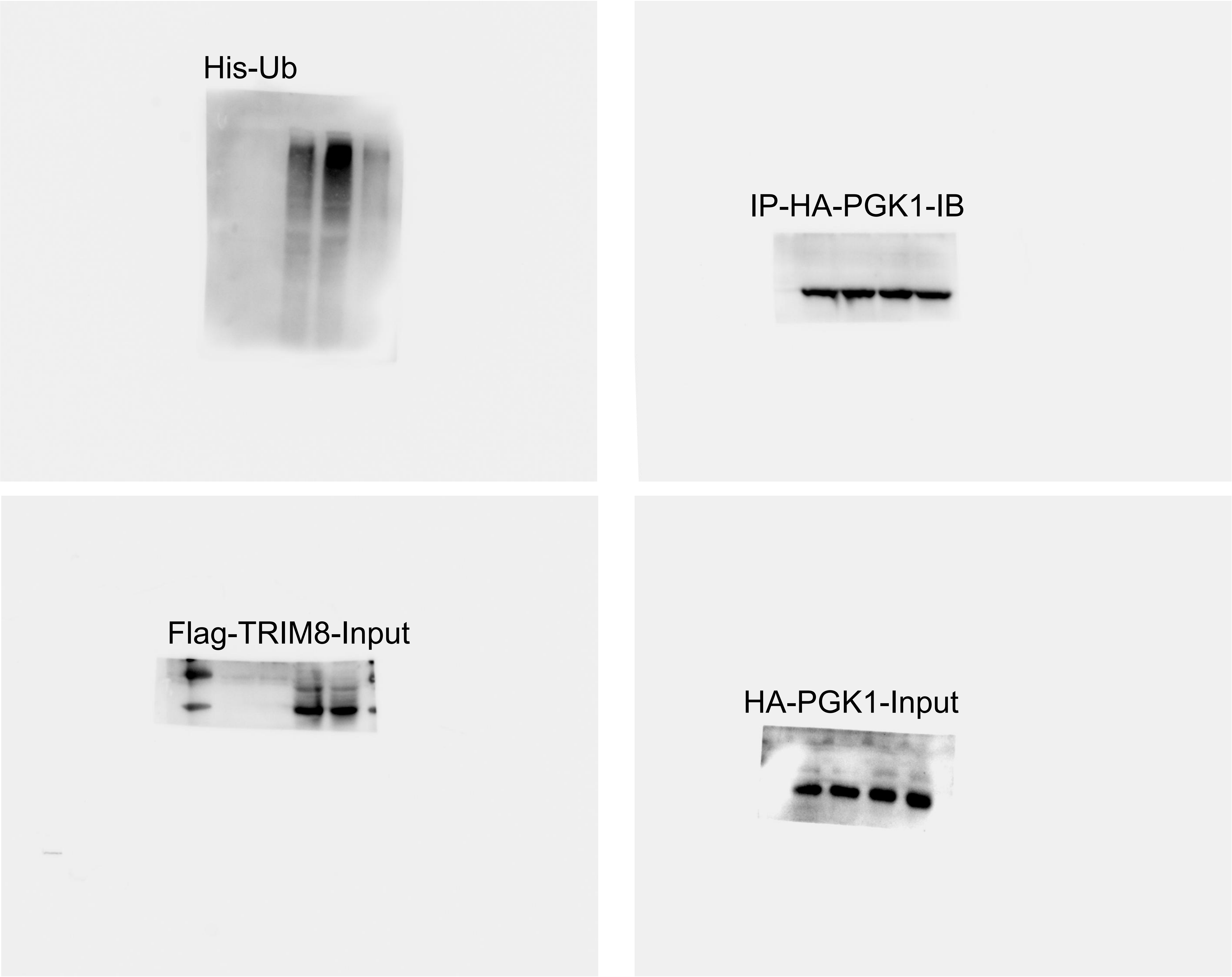

Figure 3F

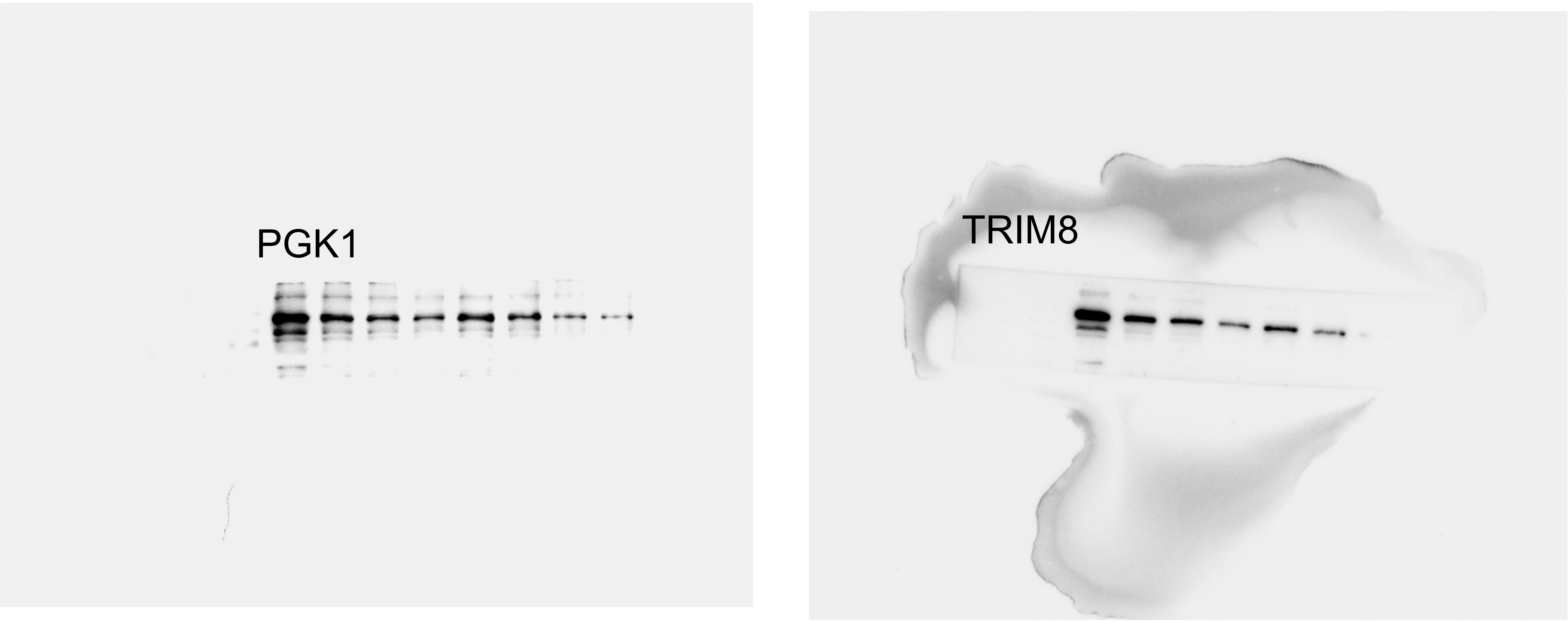

Figure 3F

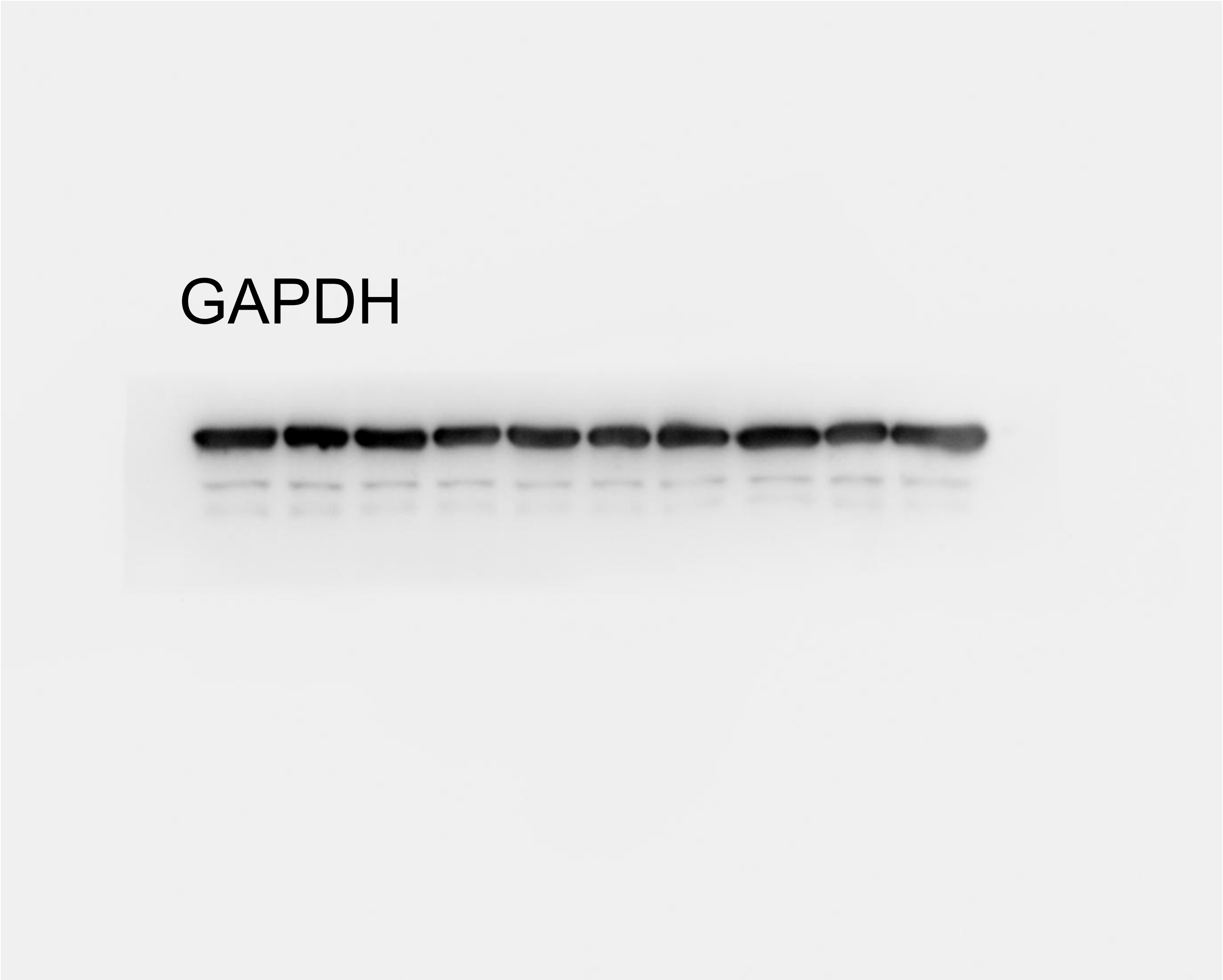

Figure 5A

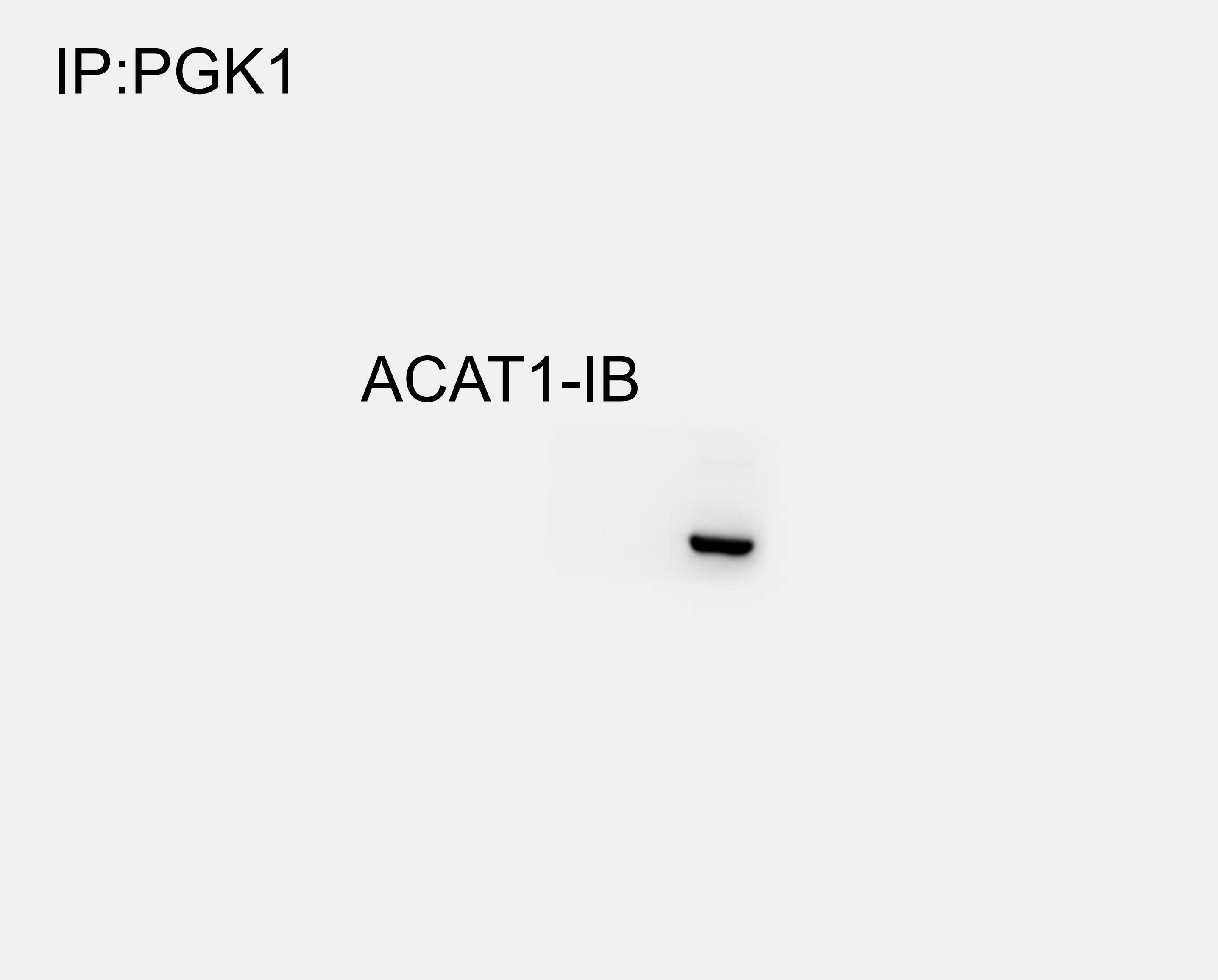

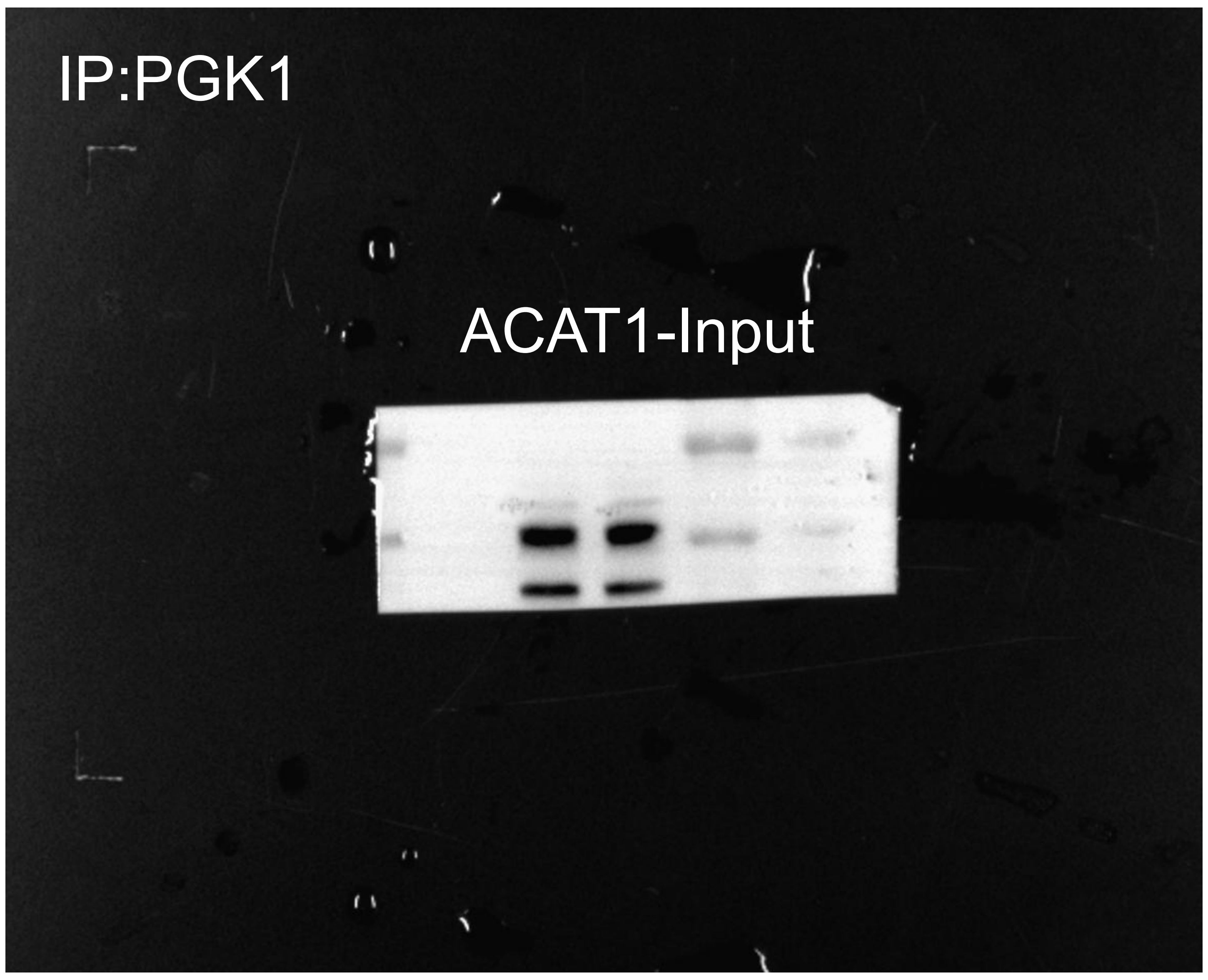

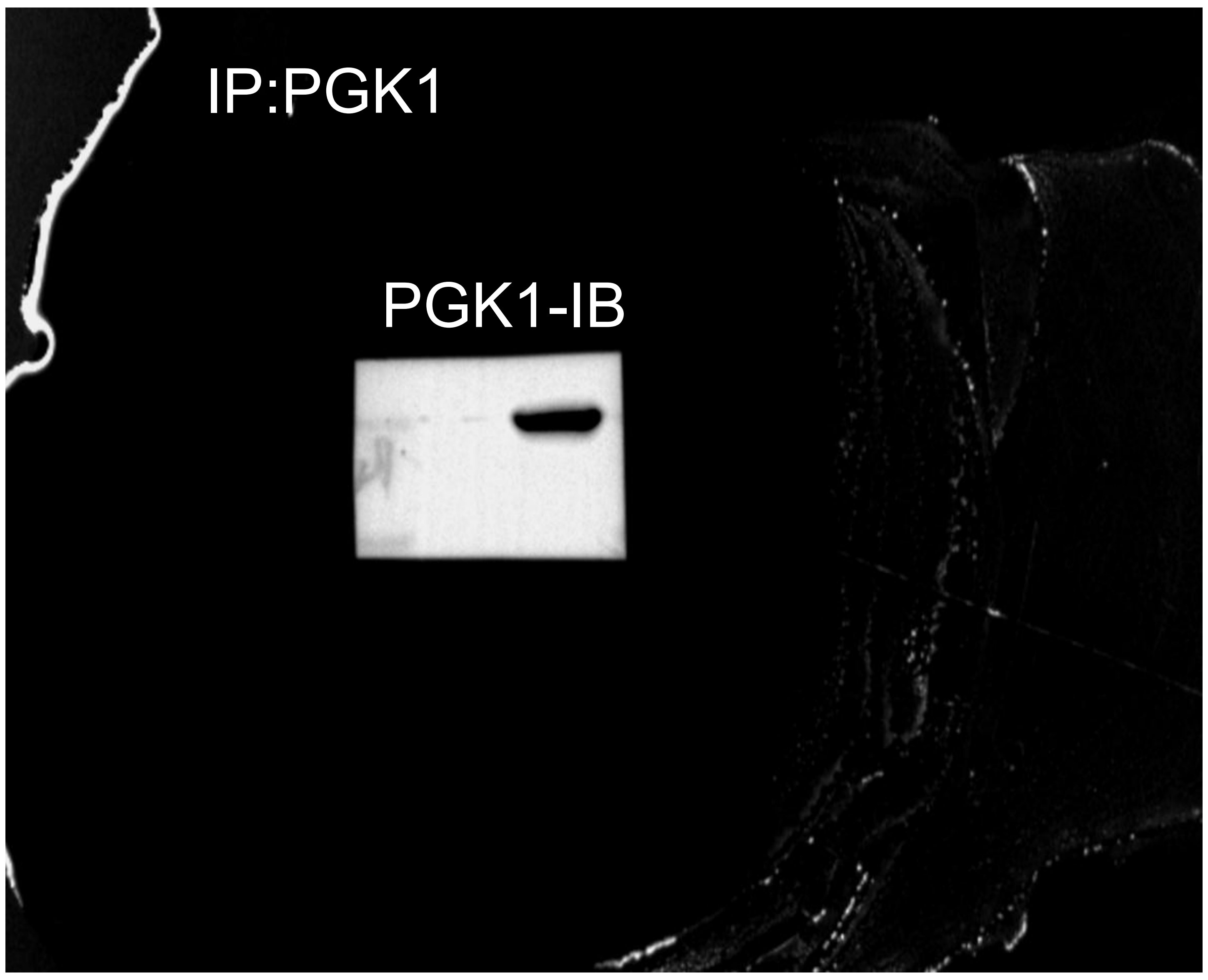

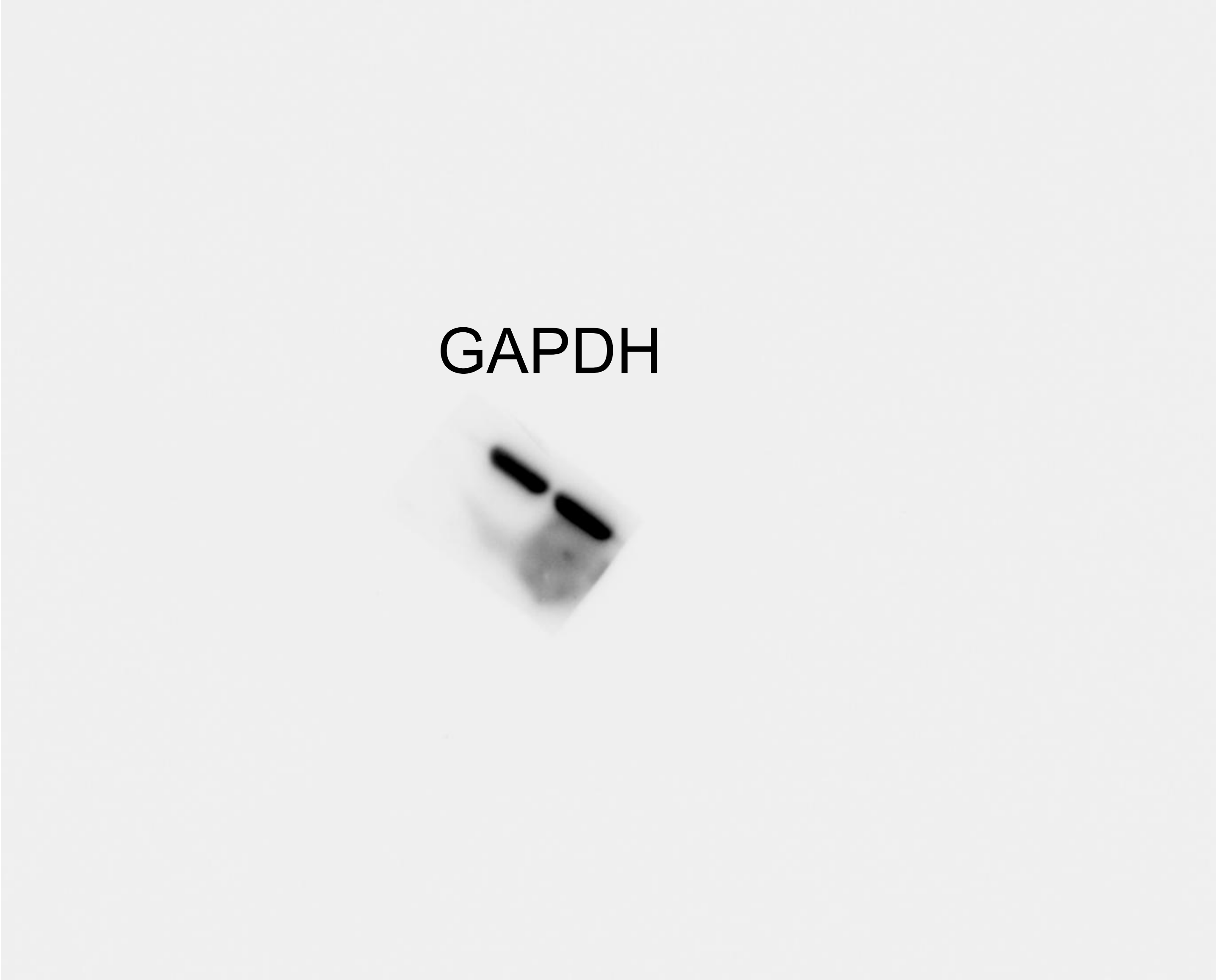

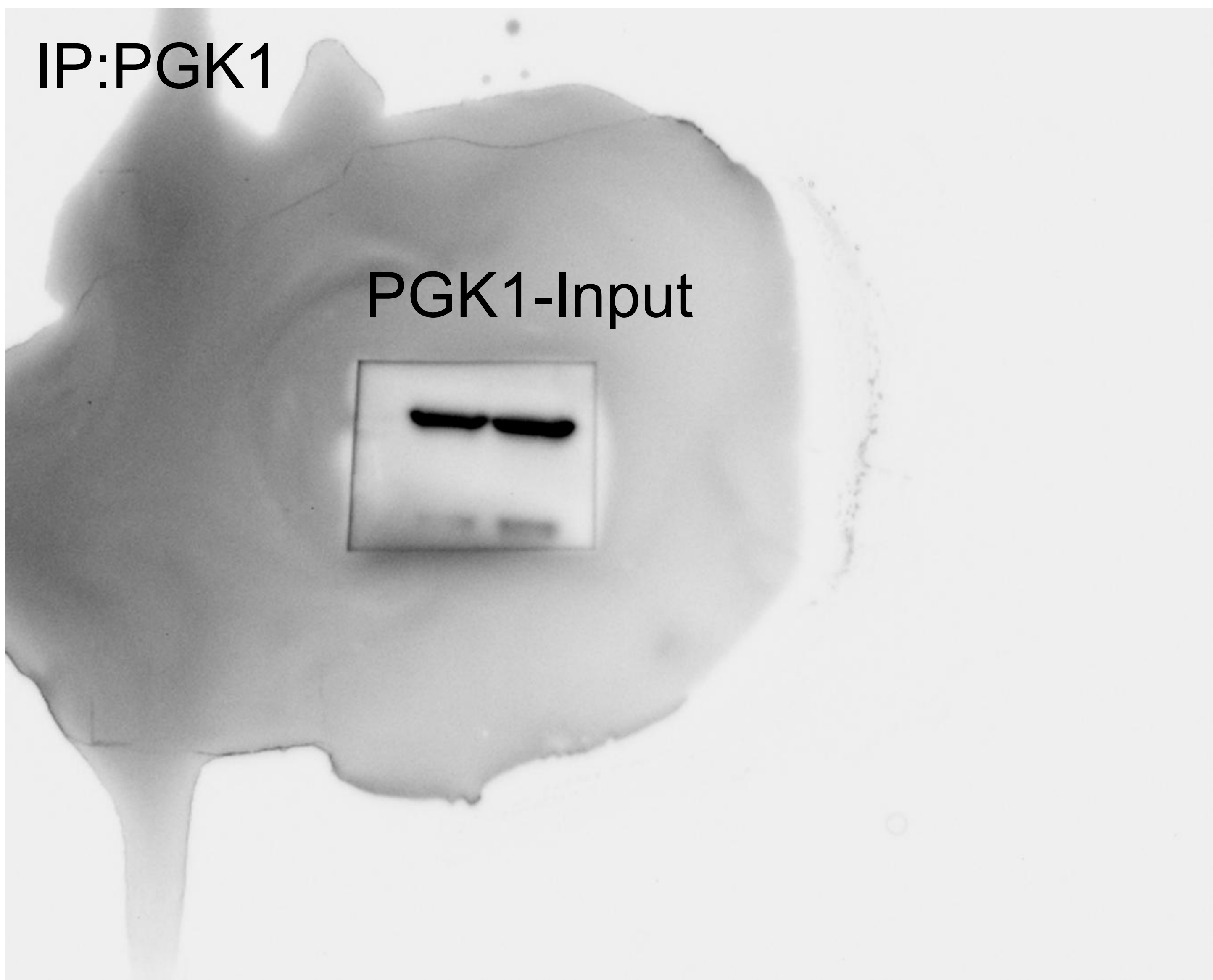

Figure 5B

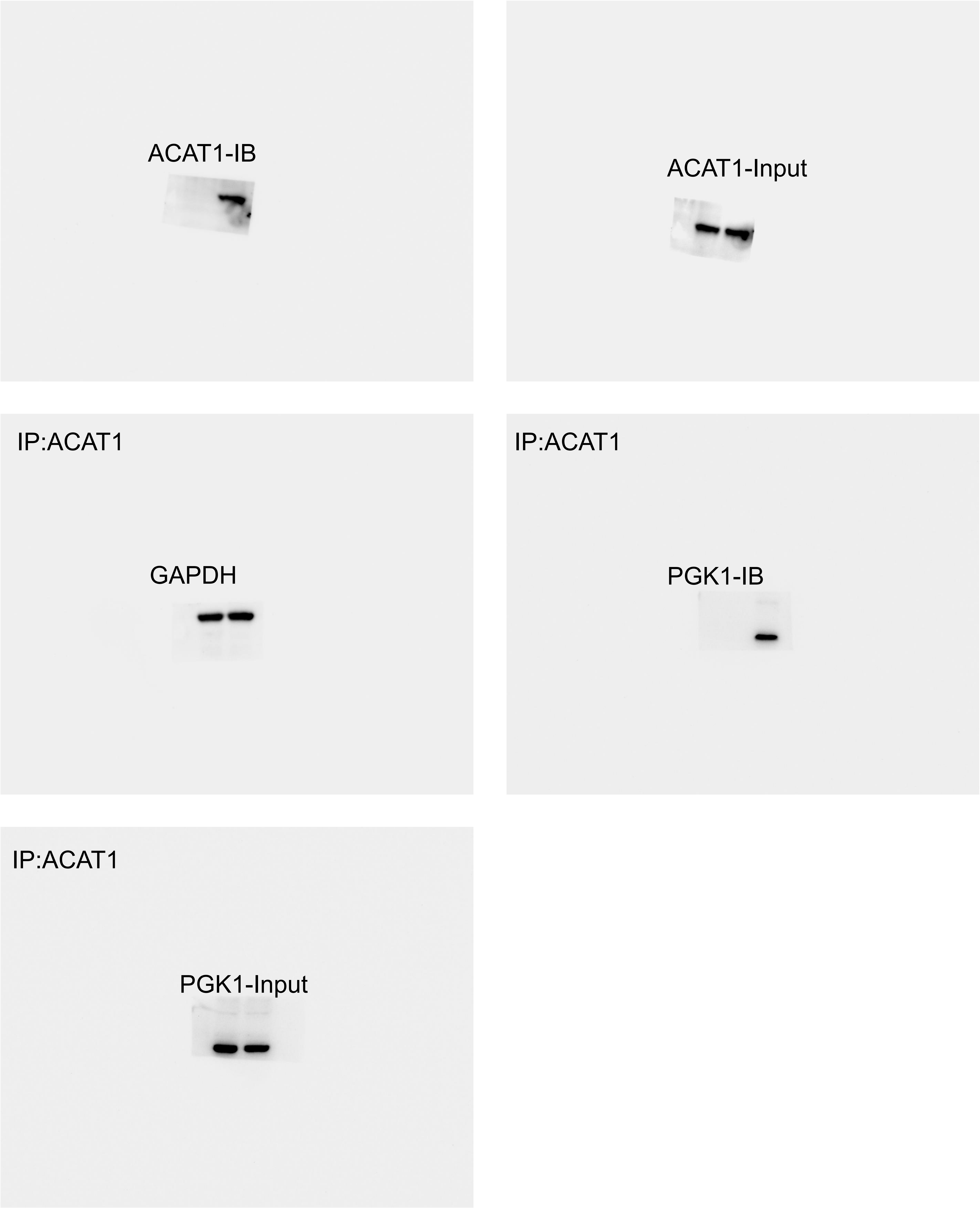
ACAT1-IB
ACAT1-Input
IP:ACAT1
GAPDH
IP:ACAT1
PGK1-IB
IP:ACAT1
PGK1-Input

Figure 5C

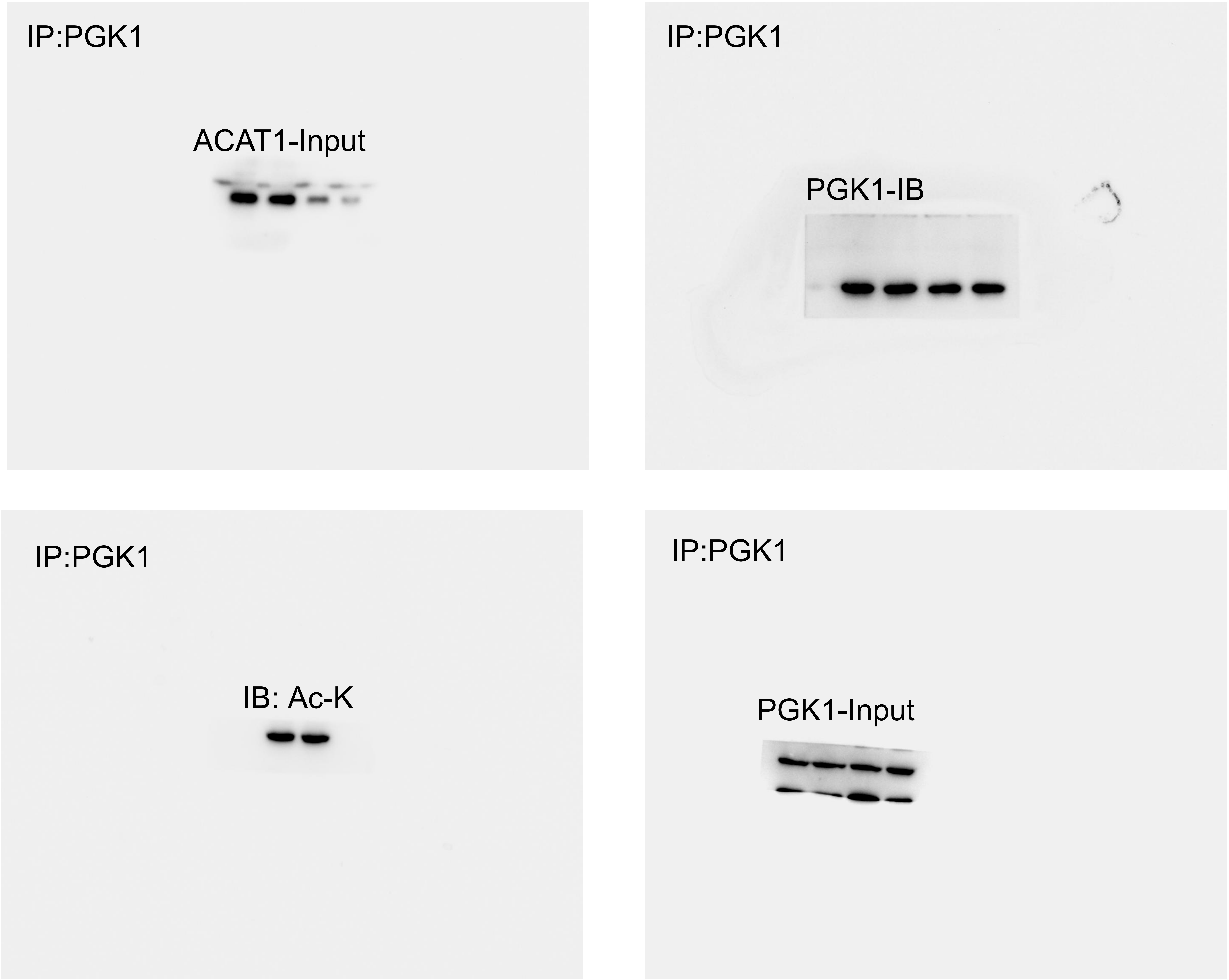

Figure 6A

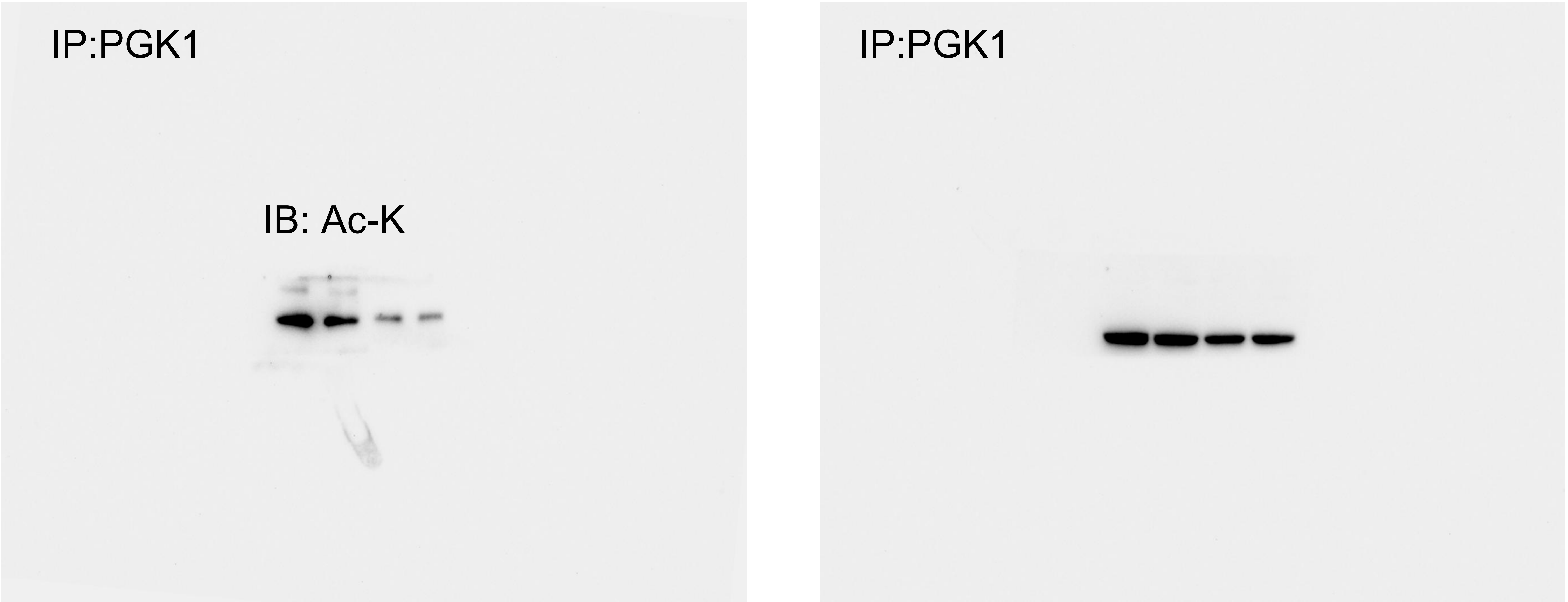

Figure 6A

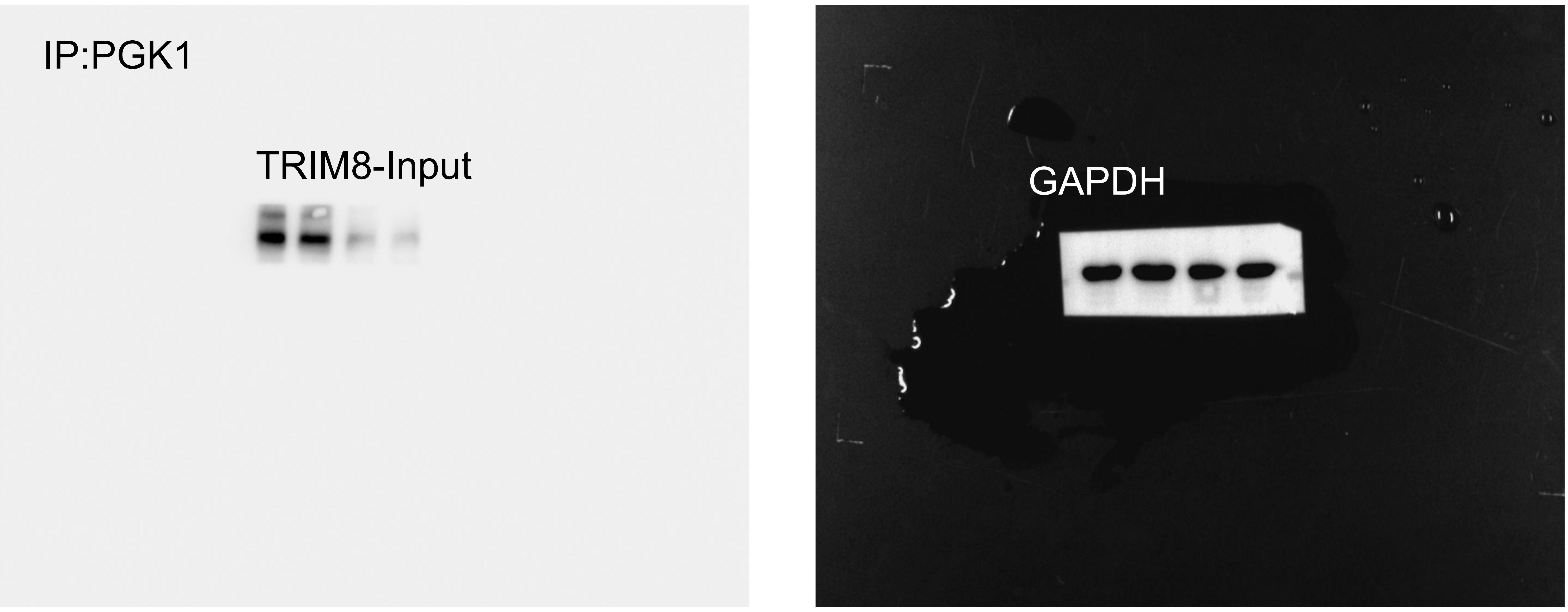

Figure 6B

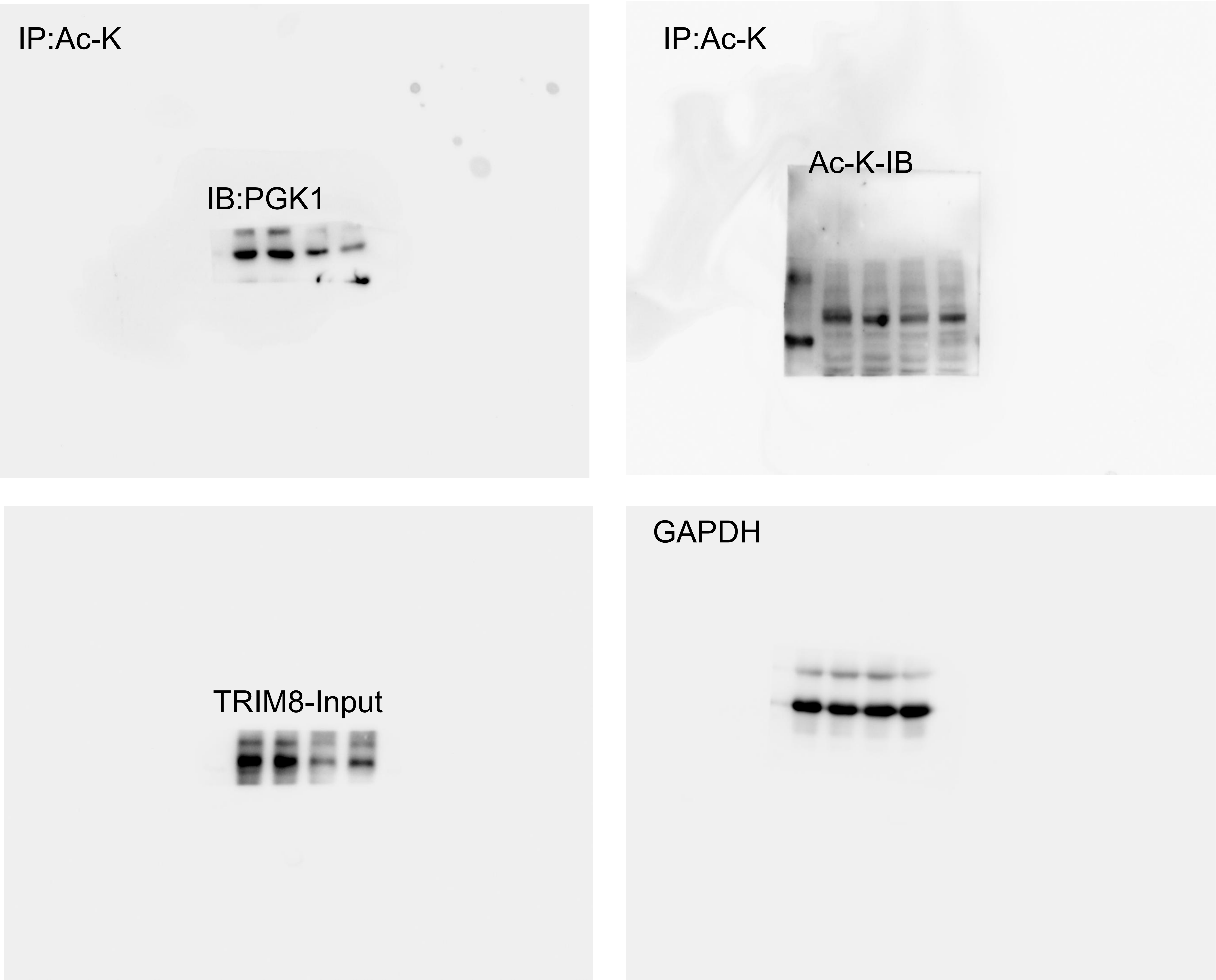

Figure 6C

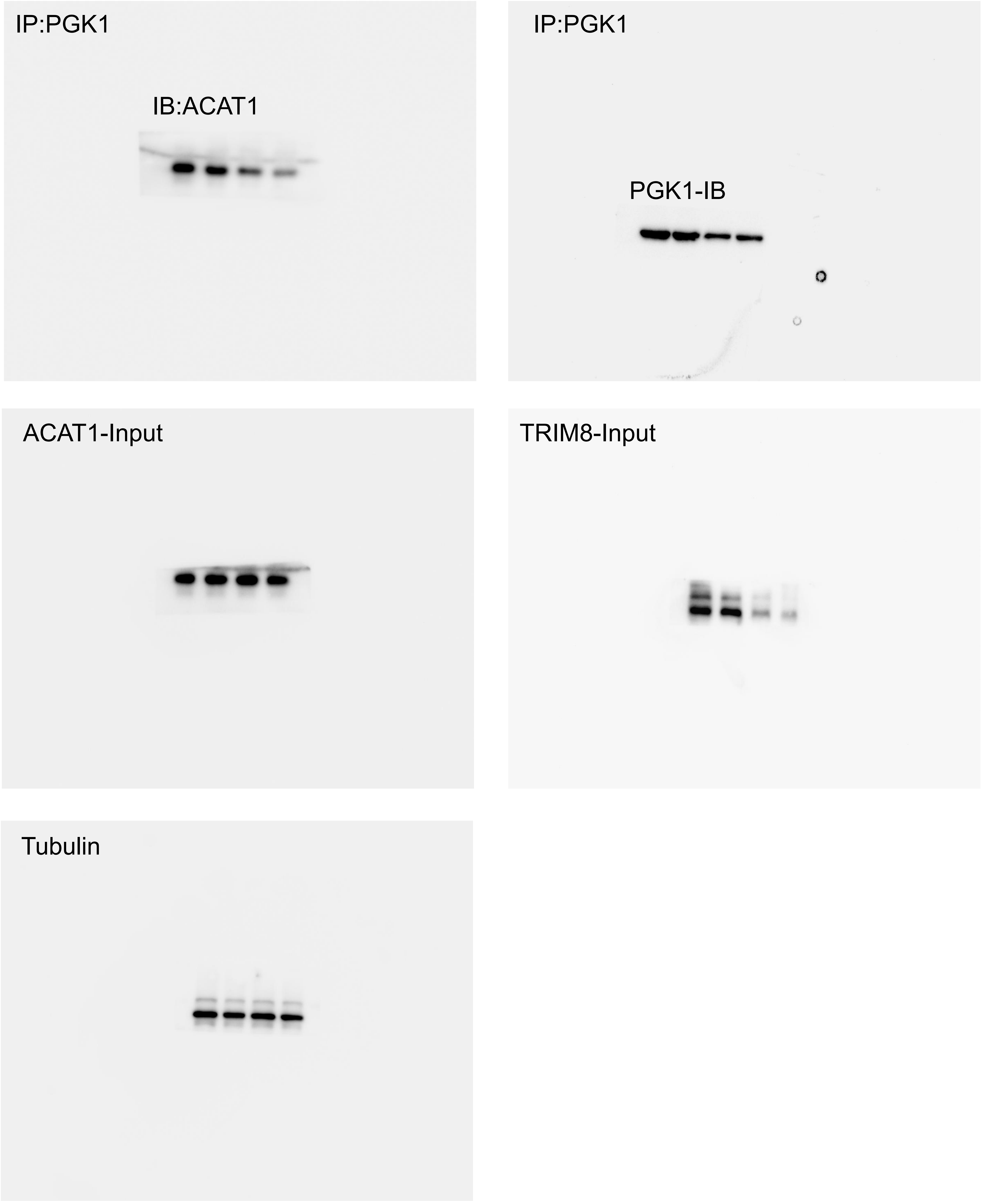
IP:PGK1
IB:ACAT1
IP:PGK1
PGK1-IB
ACAT1-Input
TRIM8-Input
Tubulin

Figure 6D

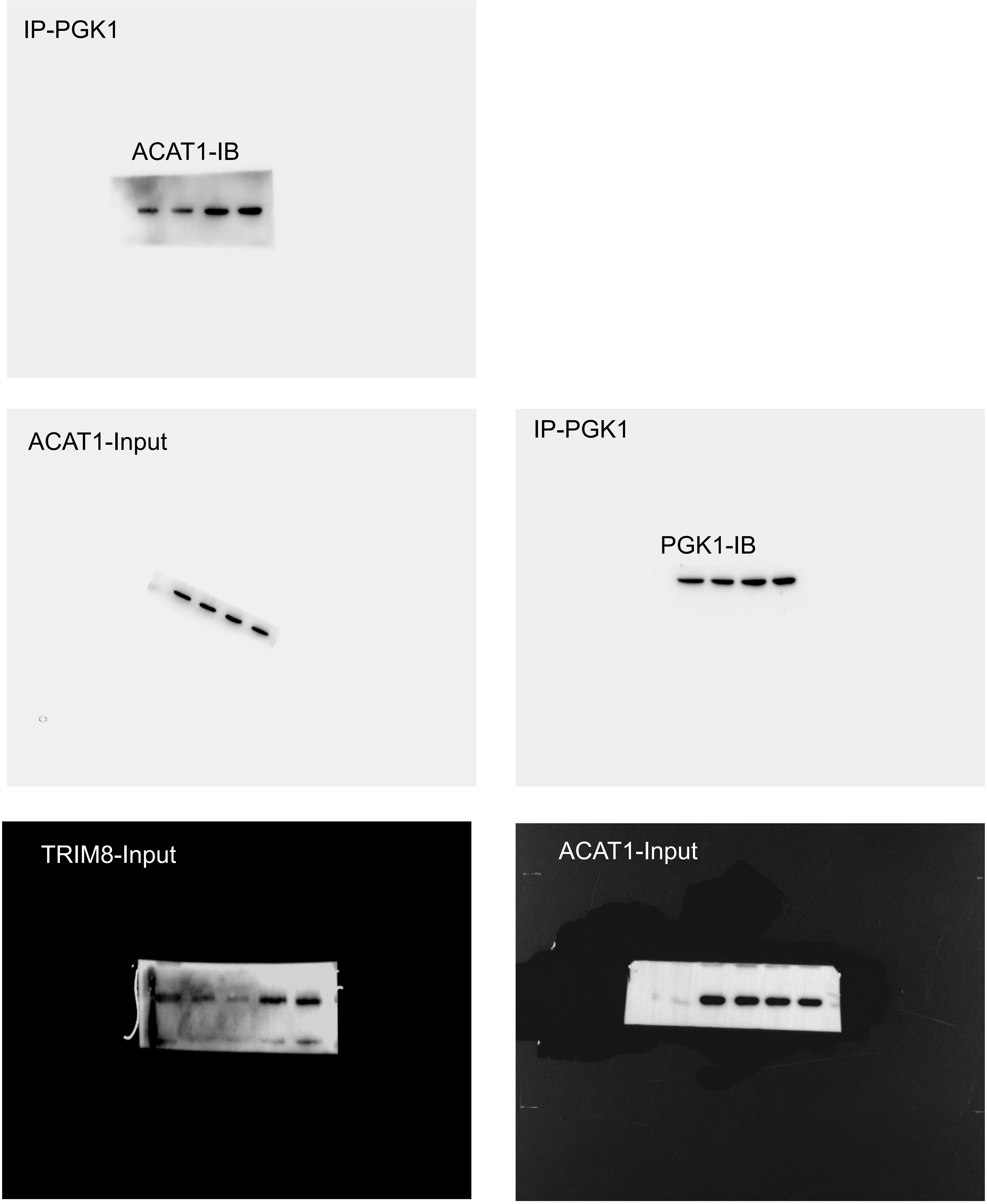

IP-PGK1
ACAT1-IB
ACAT1-Input
IP-PGK1
PGK1-IB
TRIM8-Input
ACAT1-Input

Figure 6D

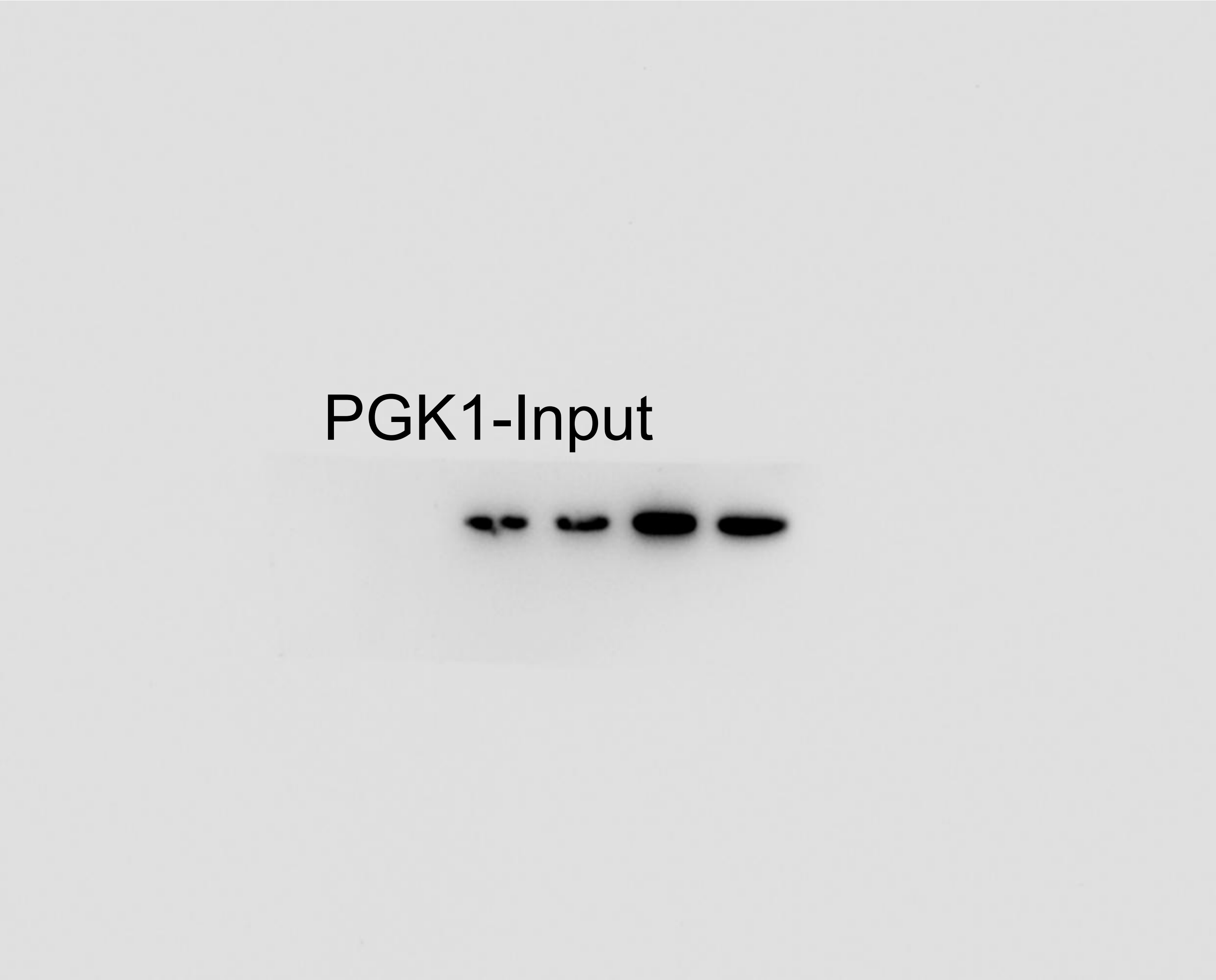

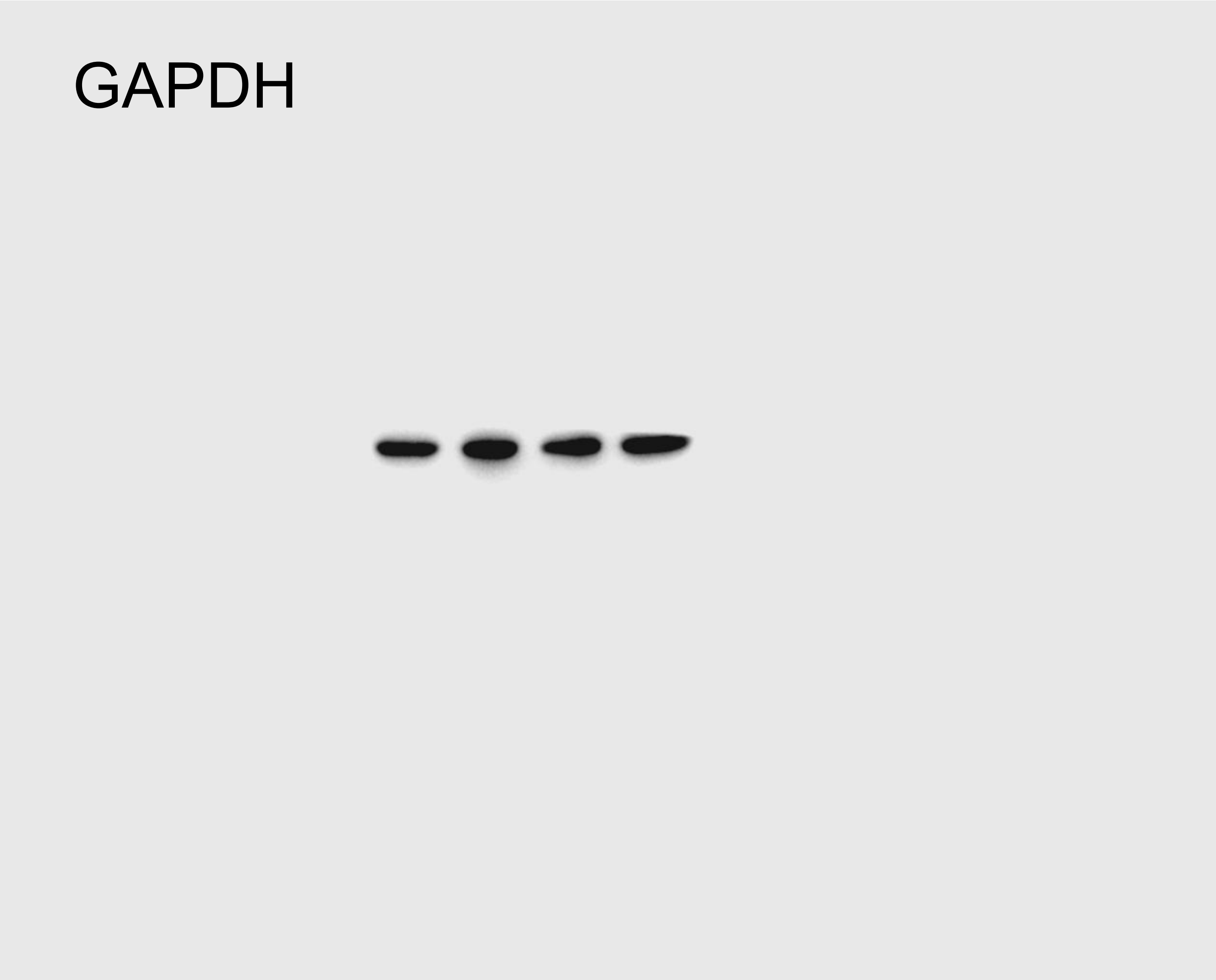

Figure 6E

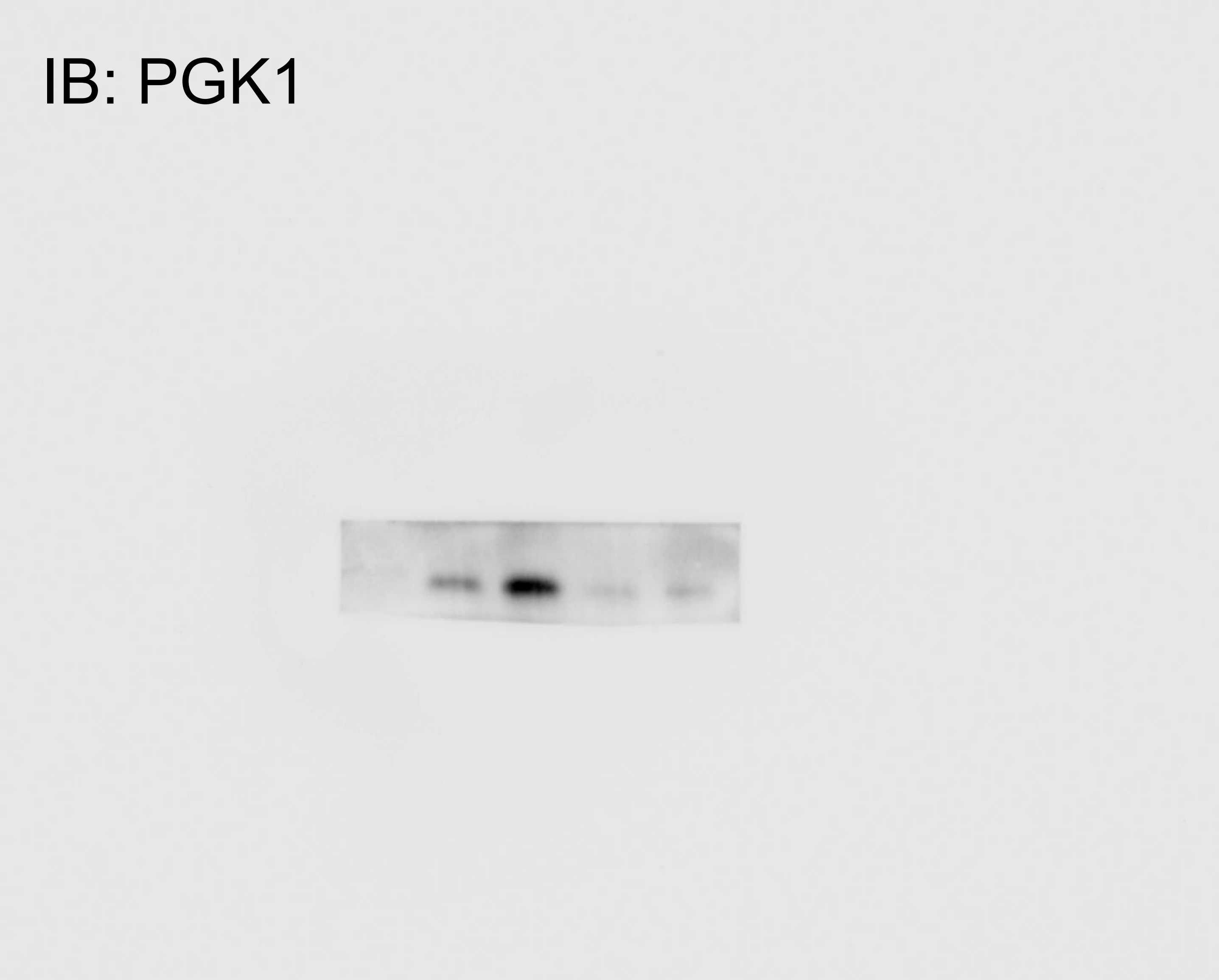

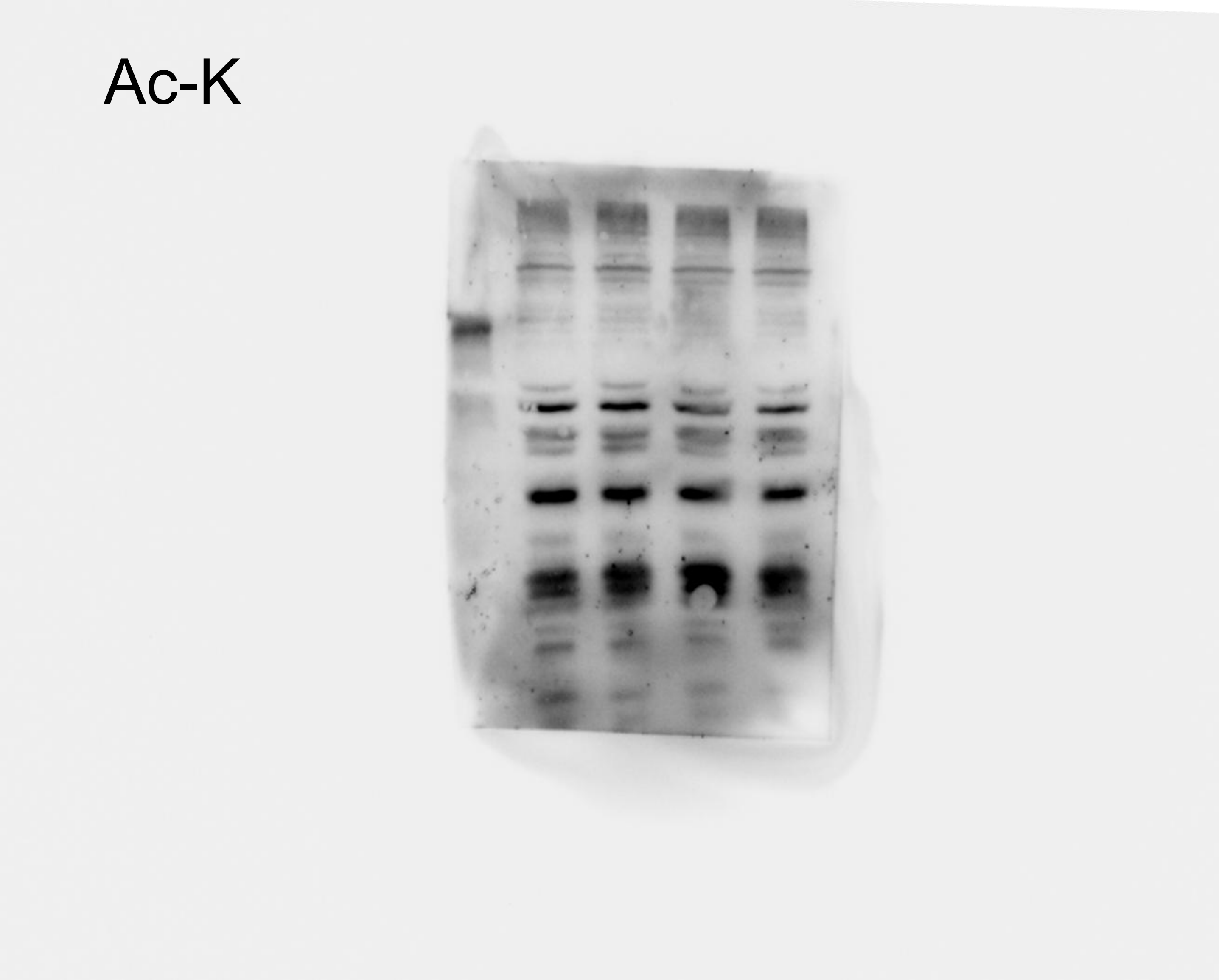

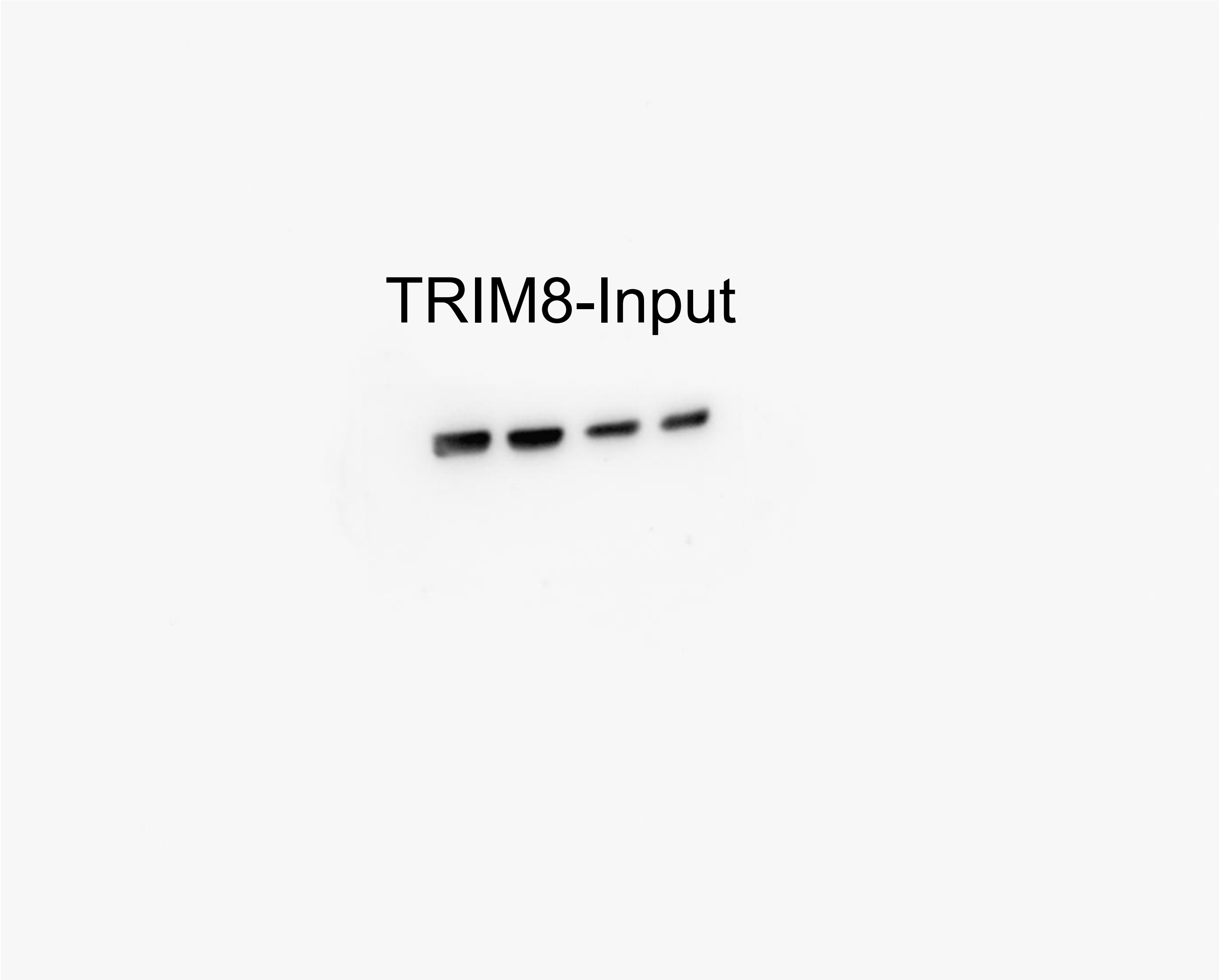

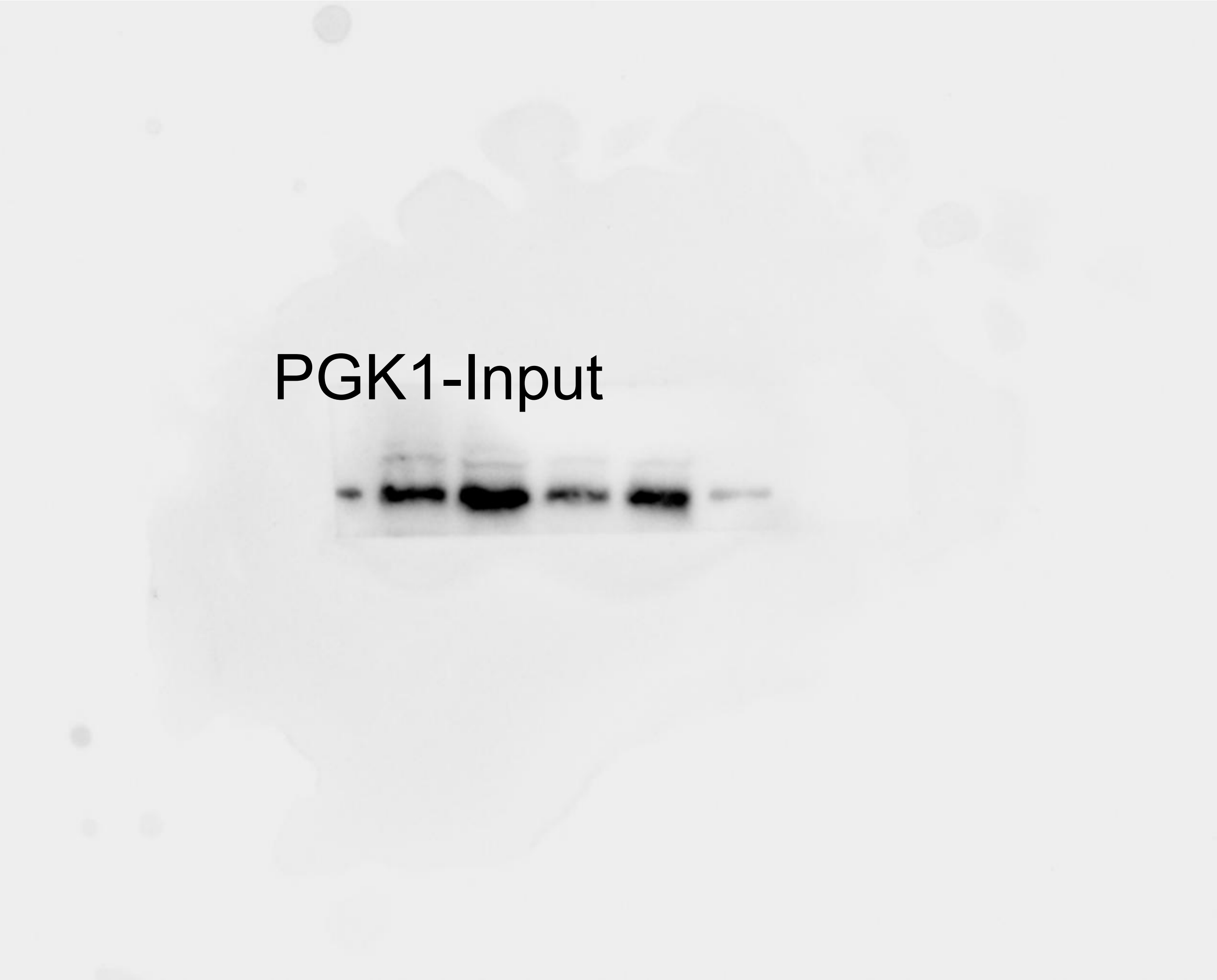

Figure 6E

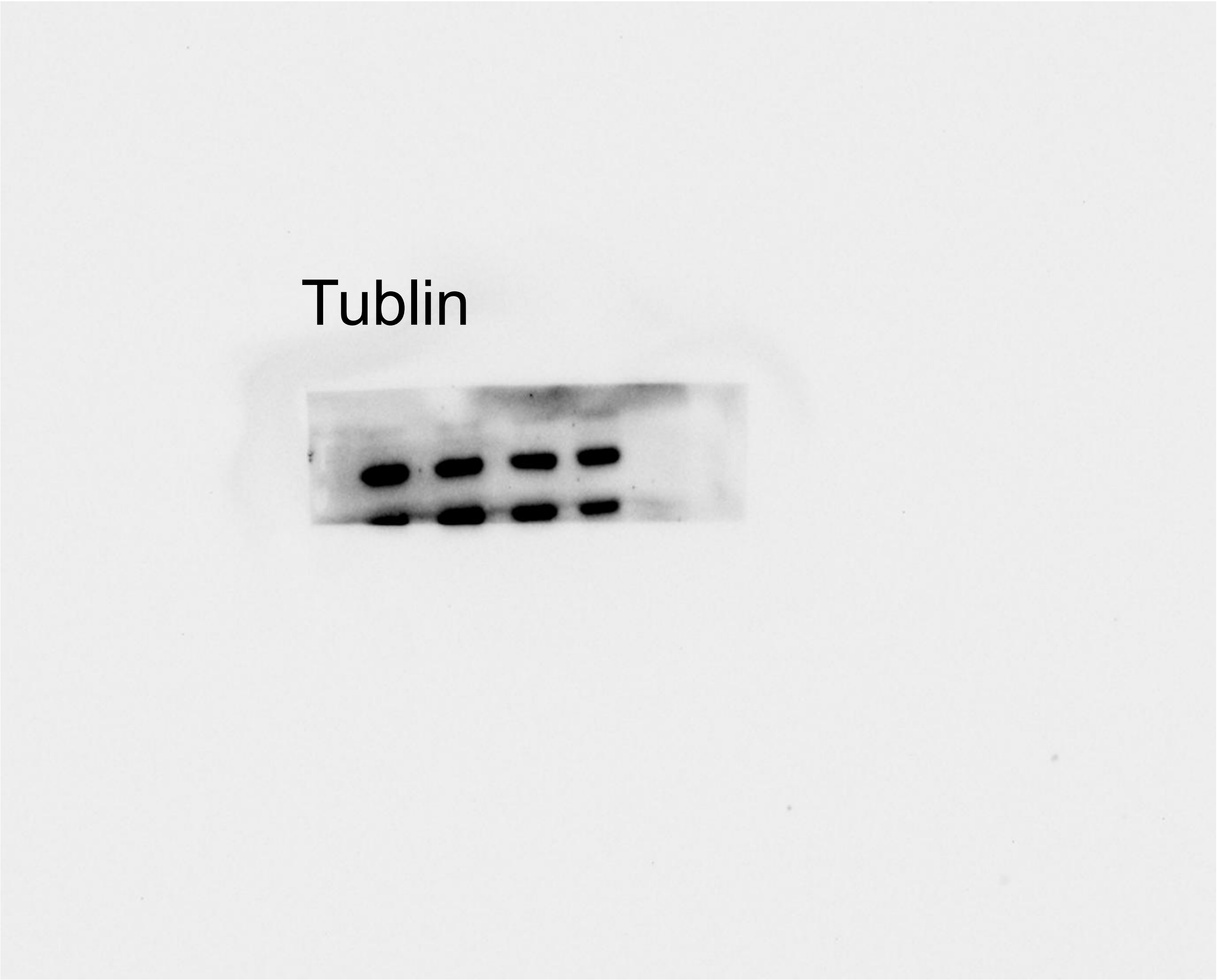

Figure 6F

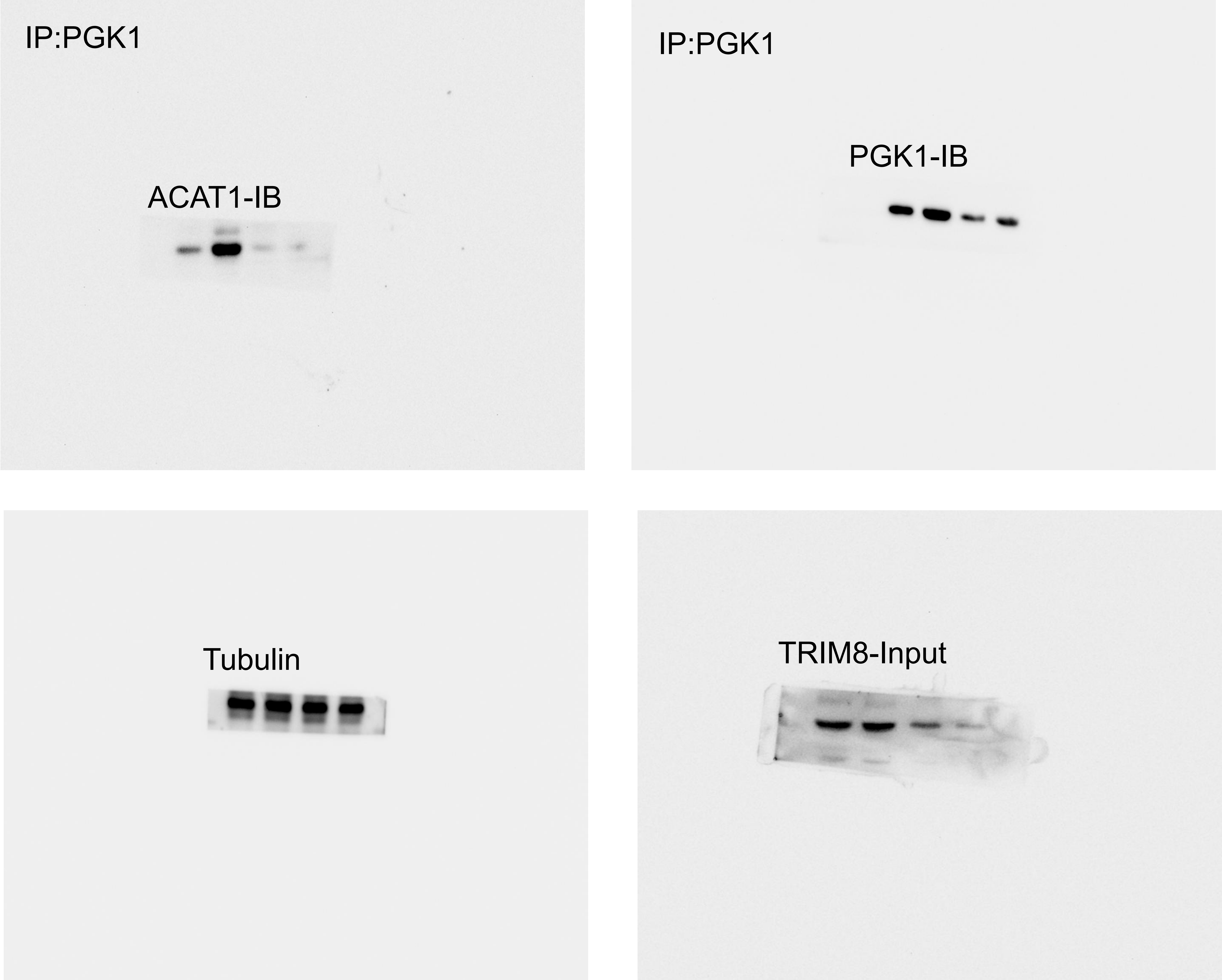

Figure 6F

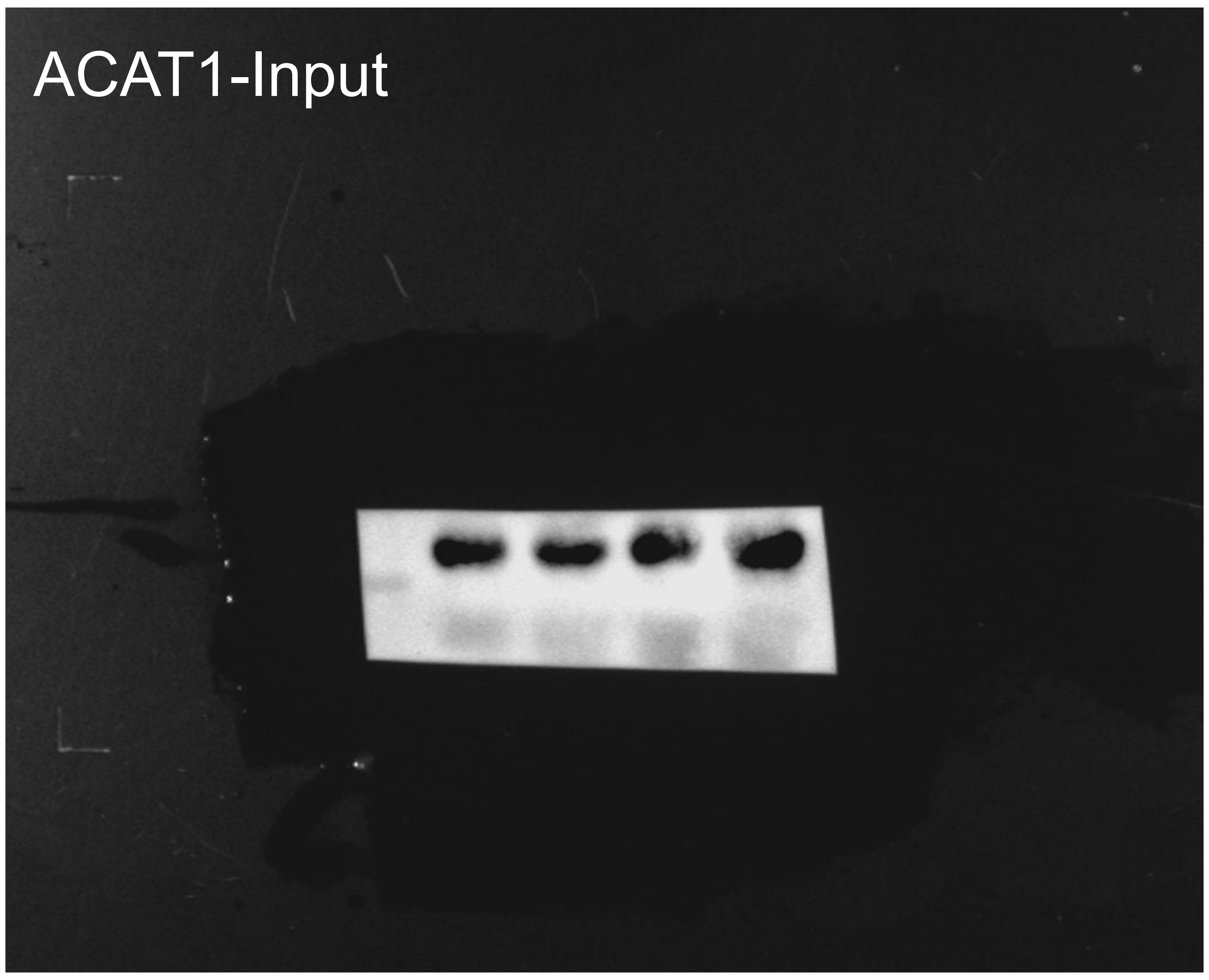

Figure 6H

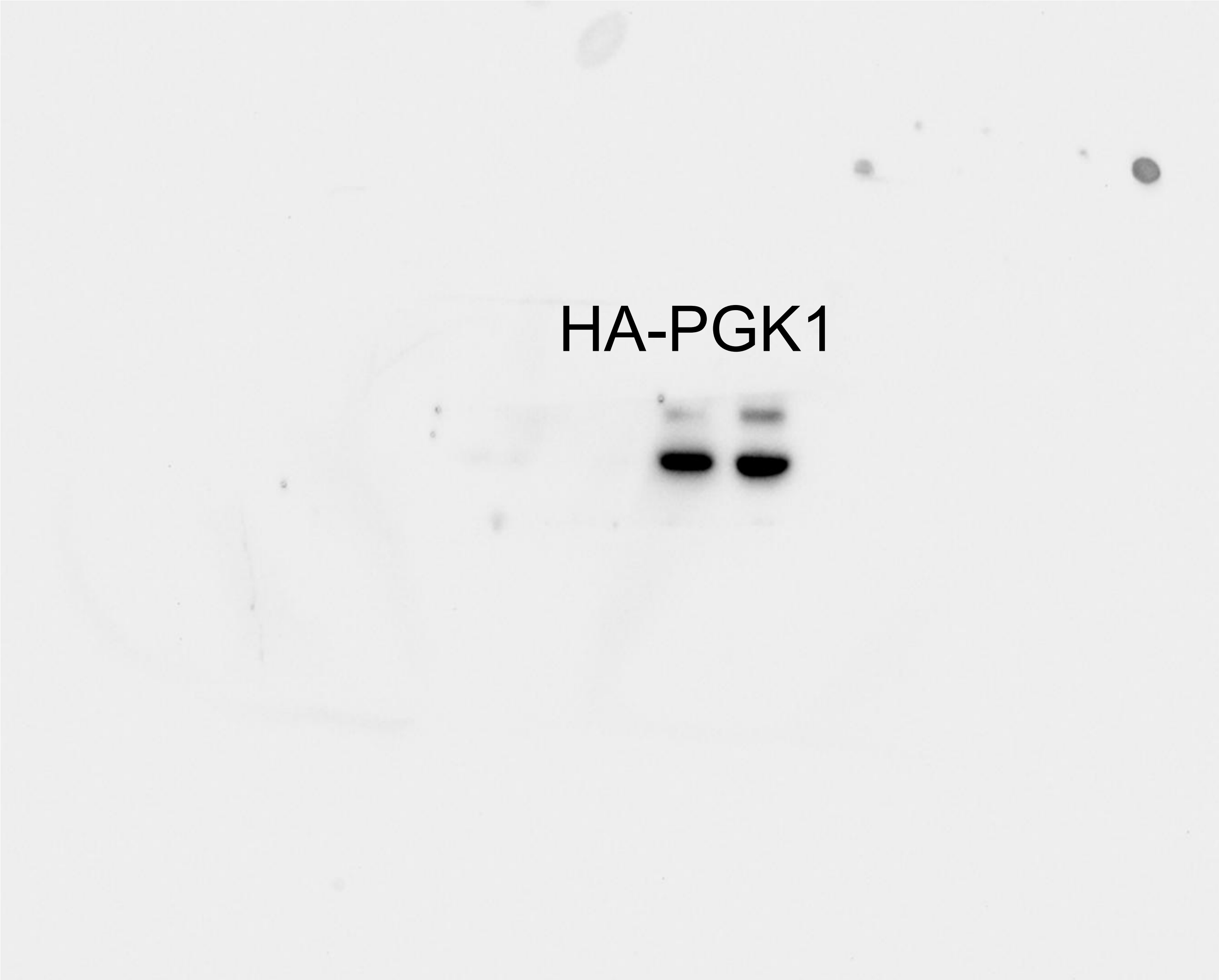

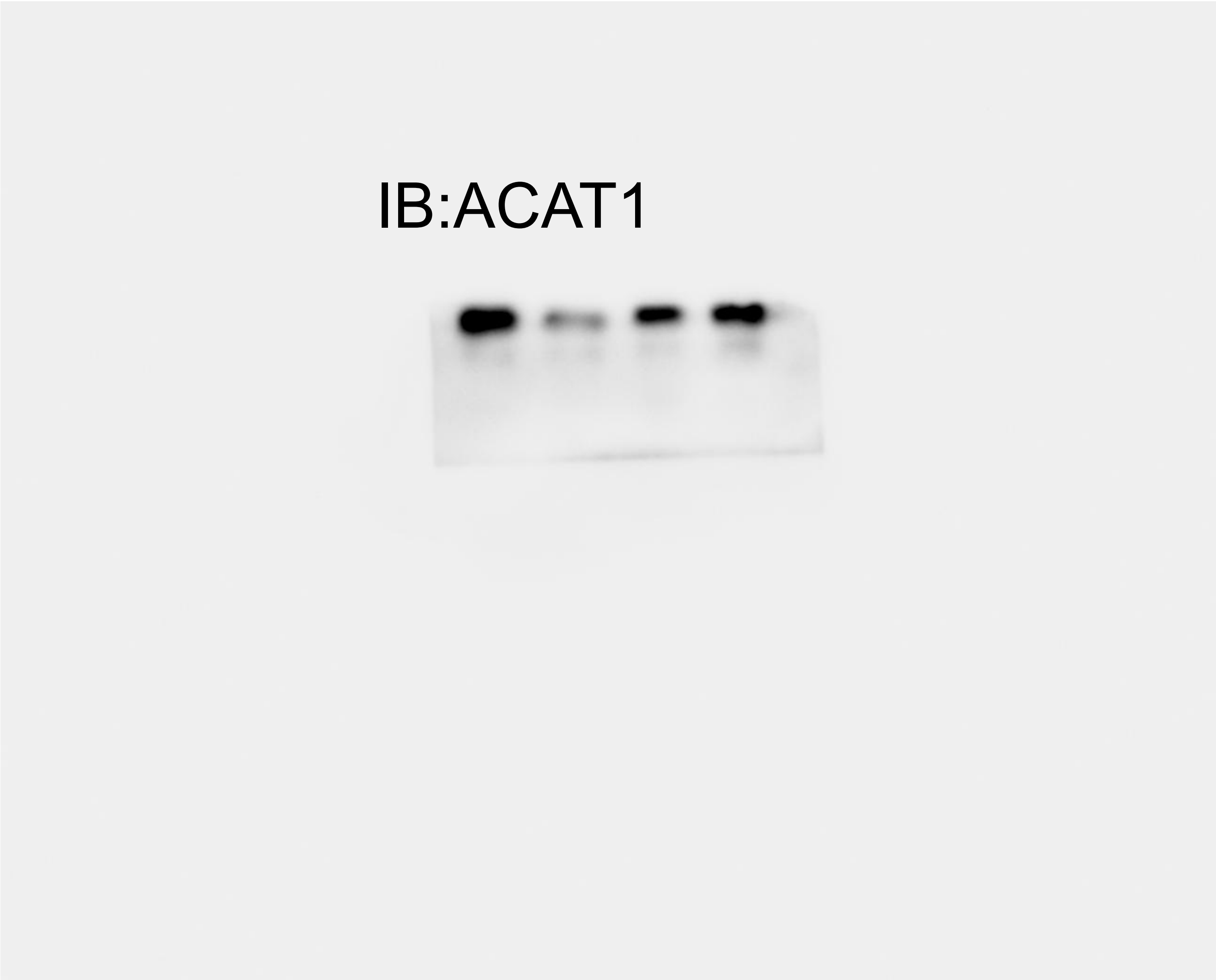

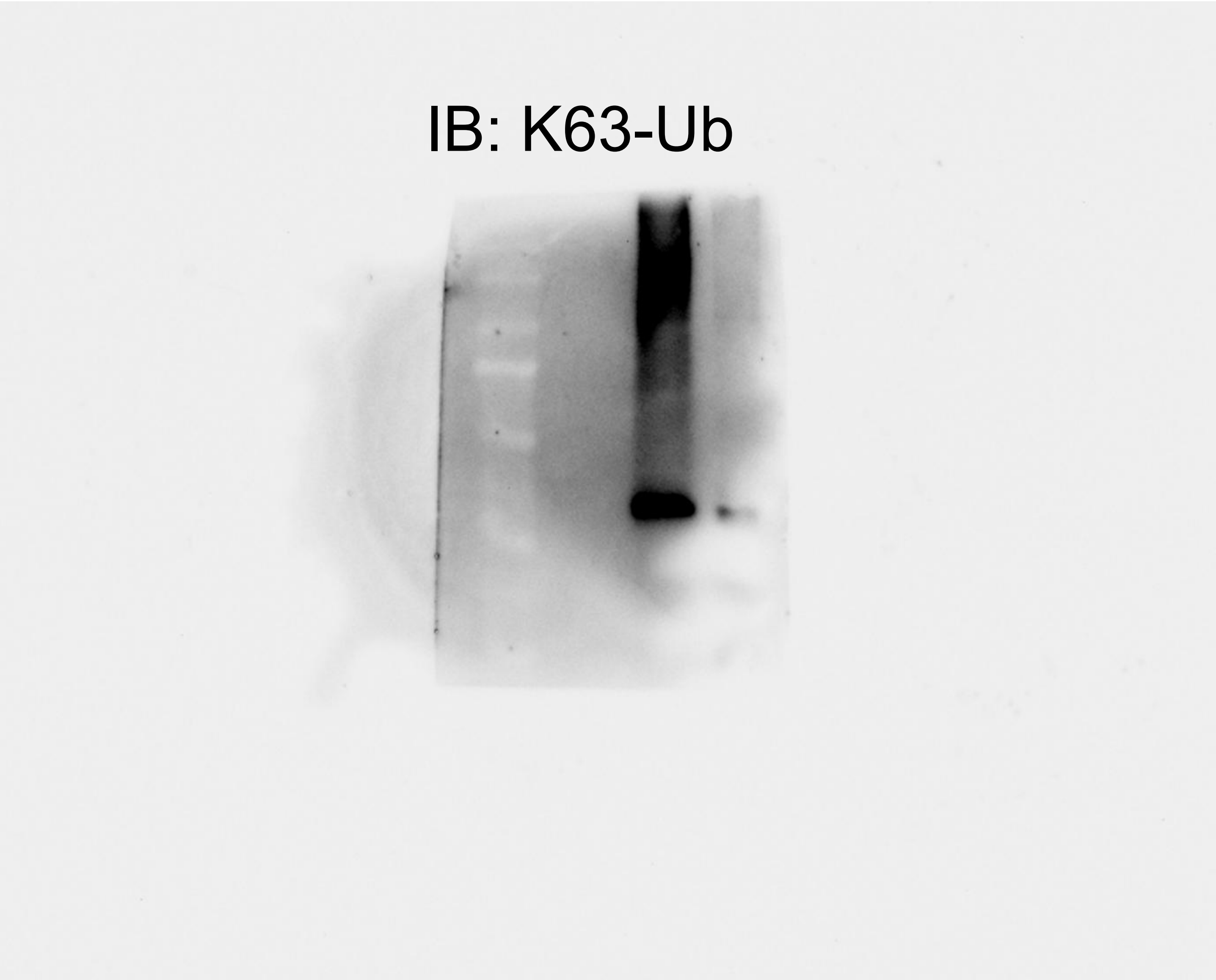

Figure S2A

SGC7901

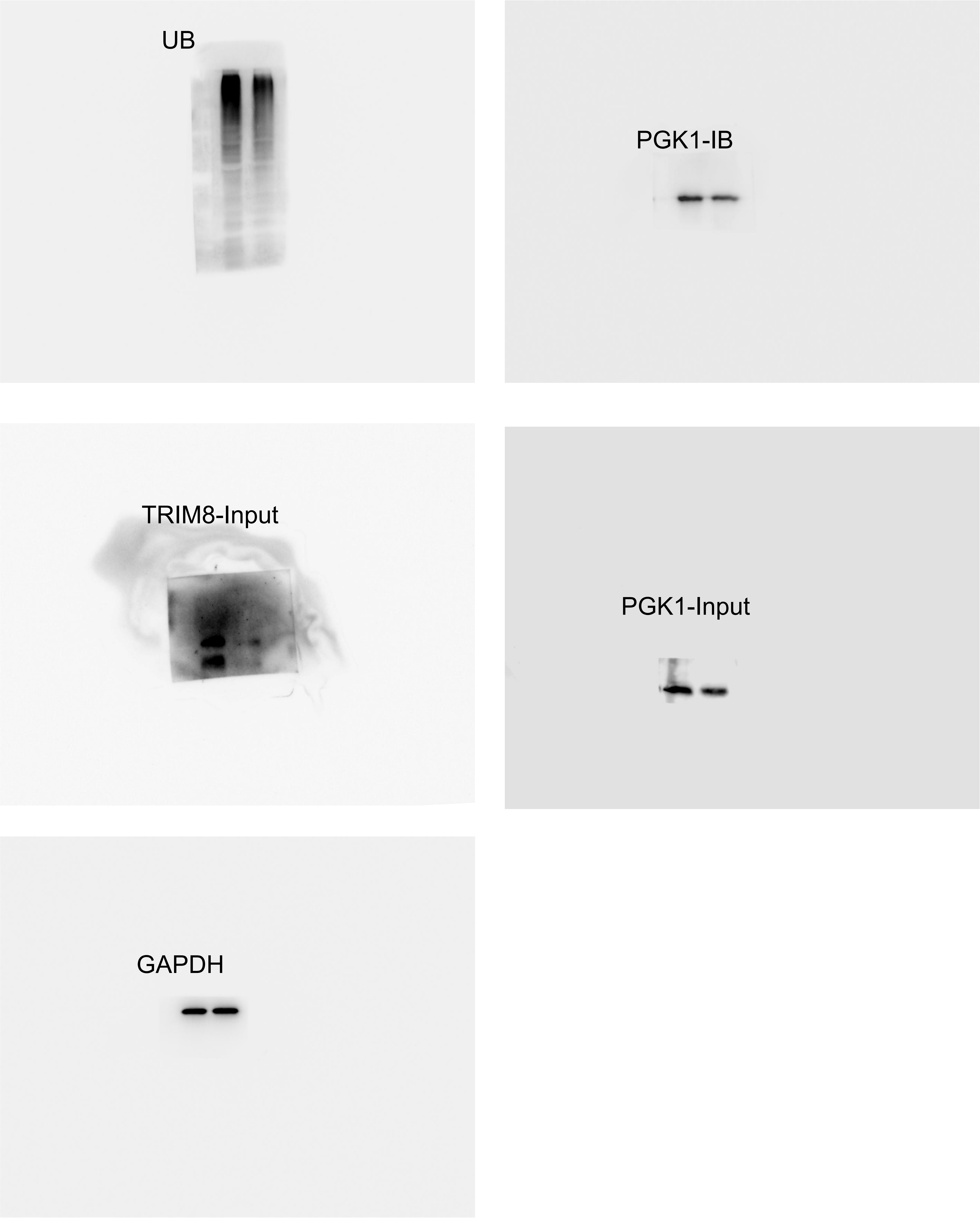

Figure S2A

BGC823

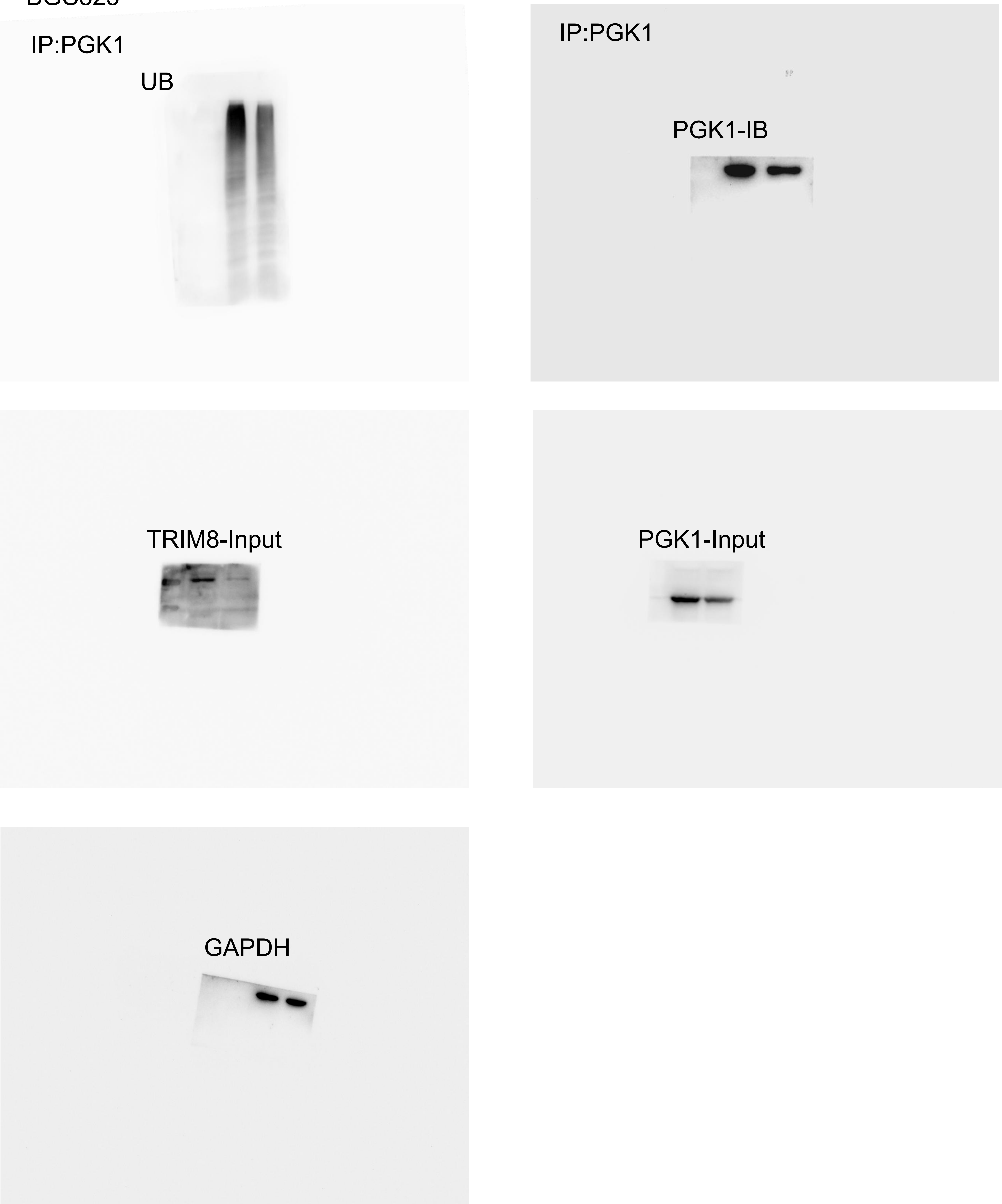

Figure S2B

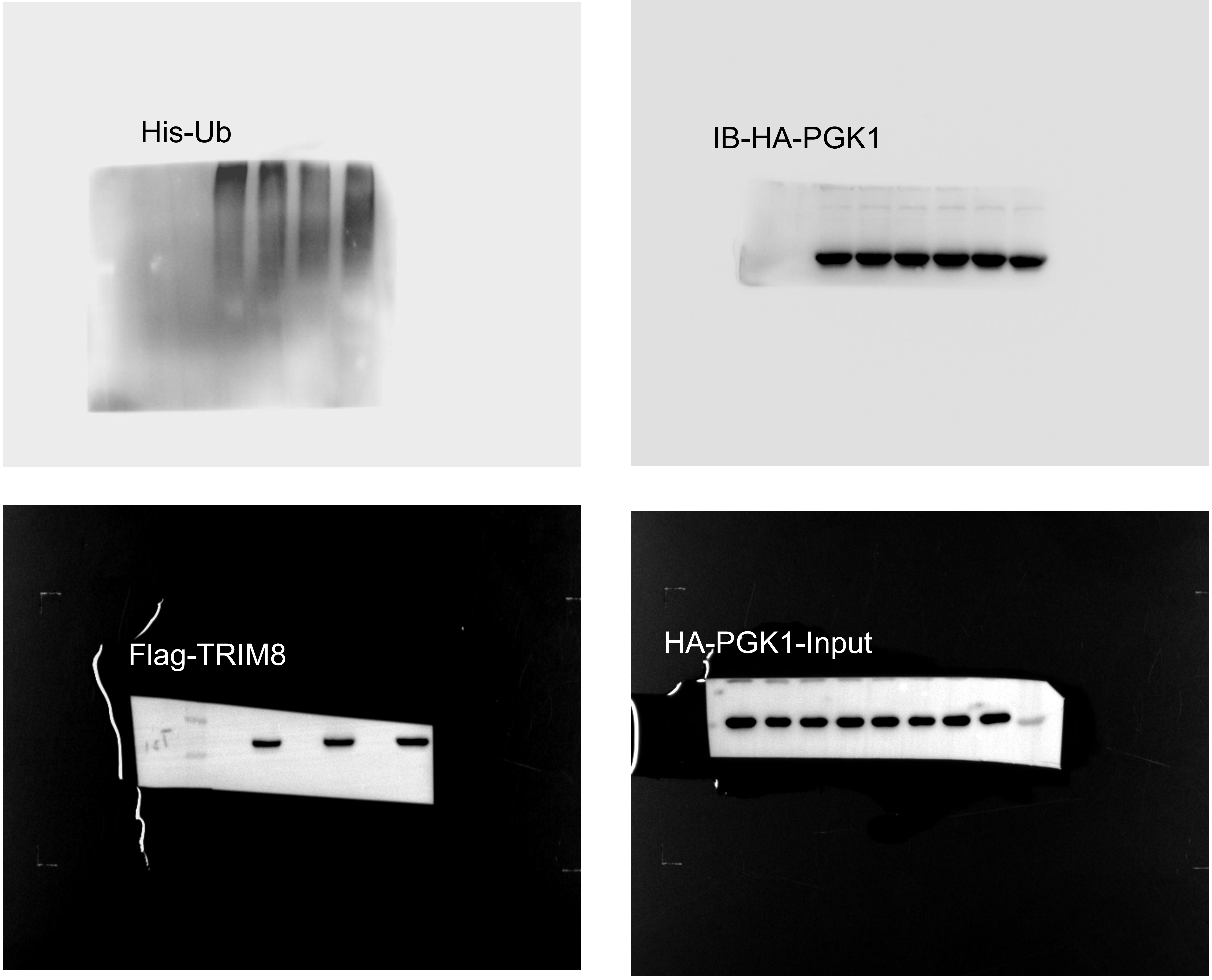

Figure S2C

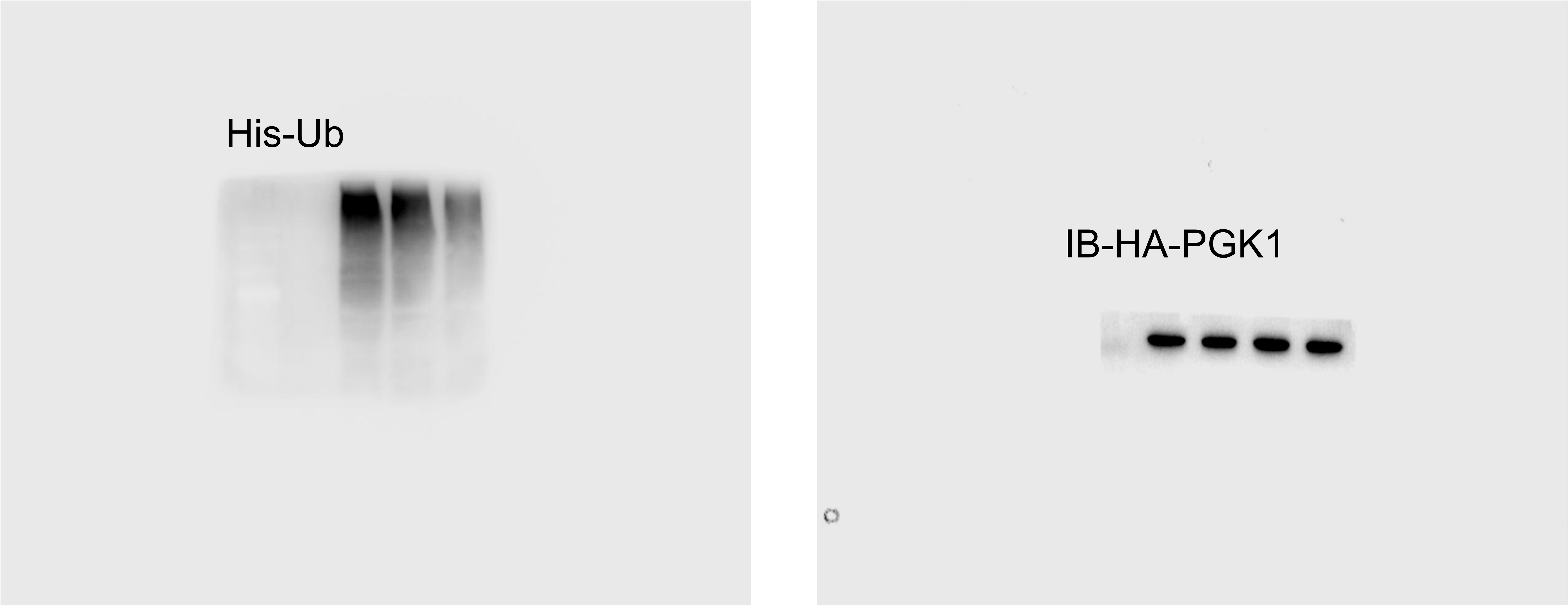

Figure S2C

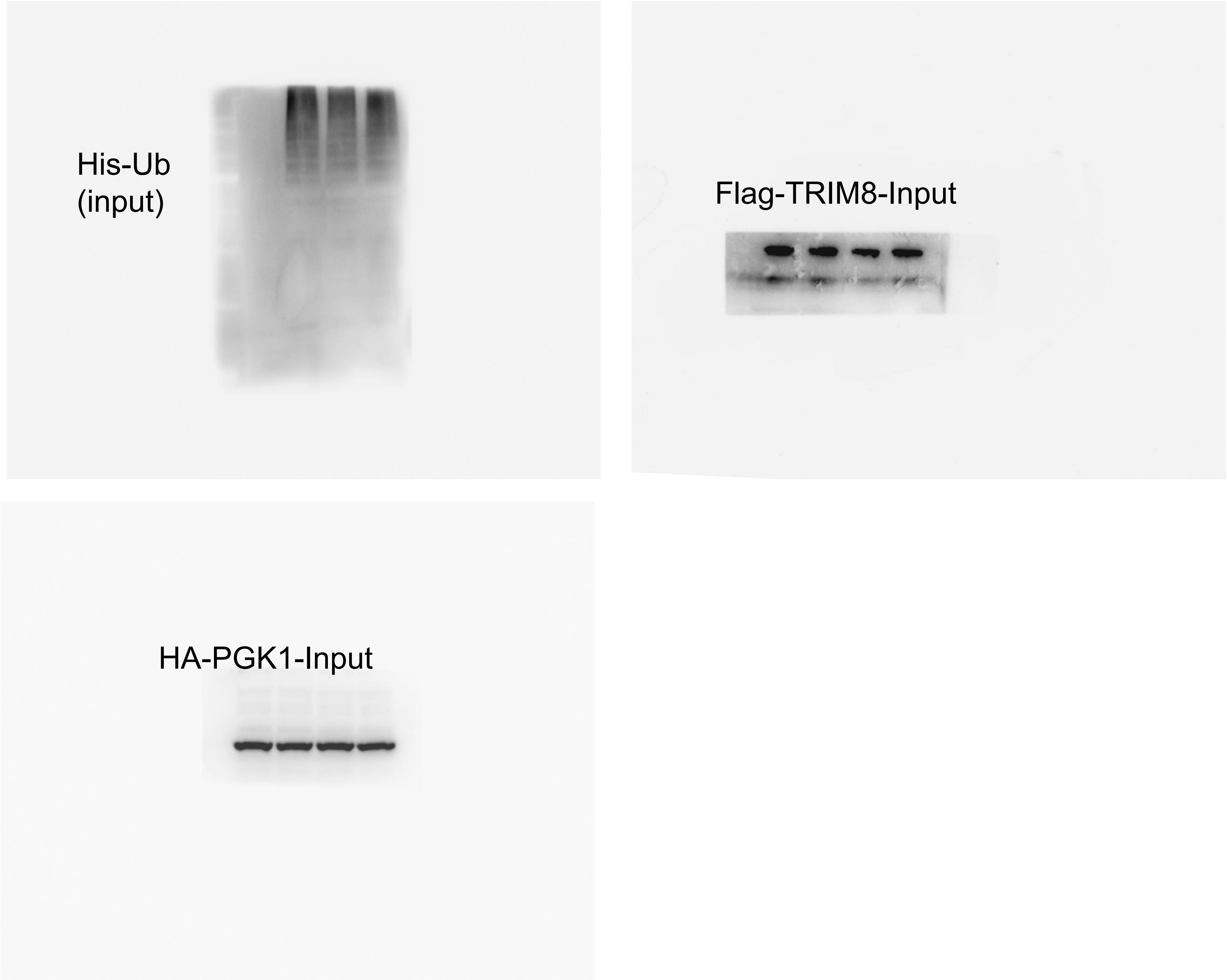

Figure S2D

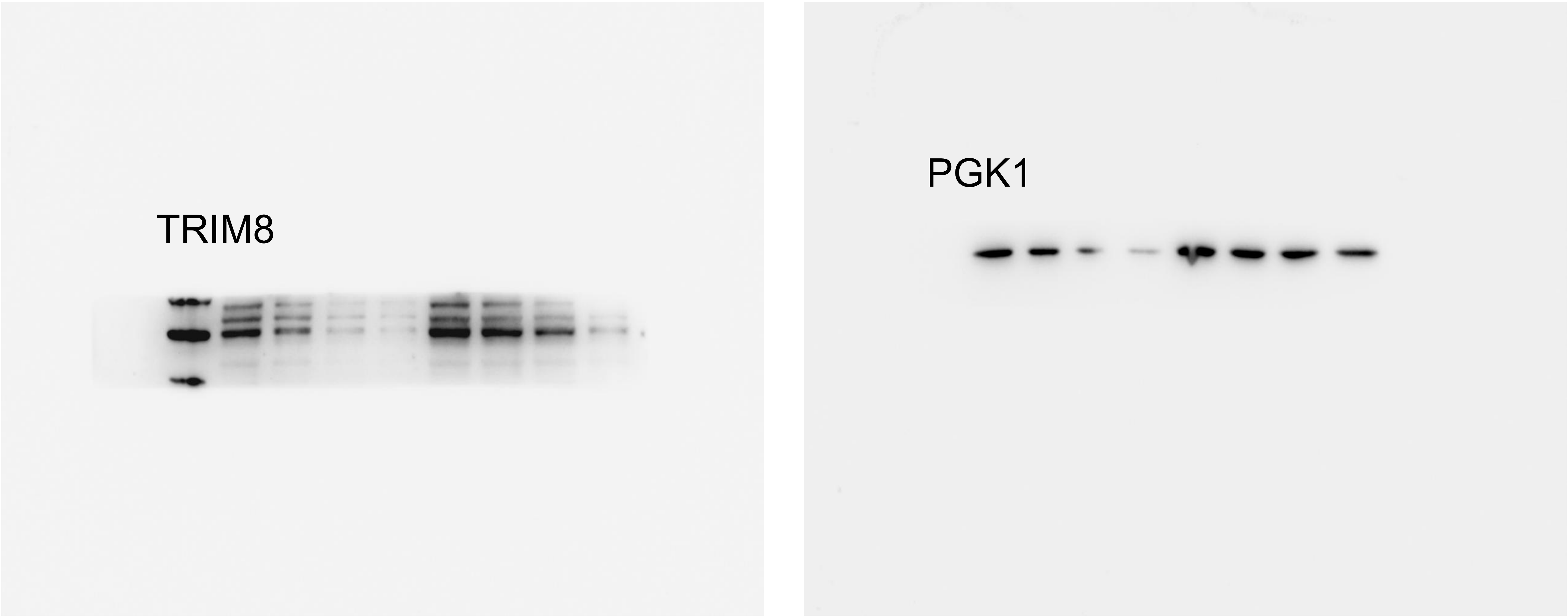

Figure S2D

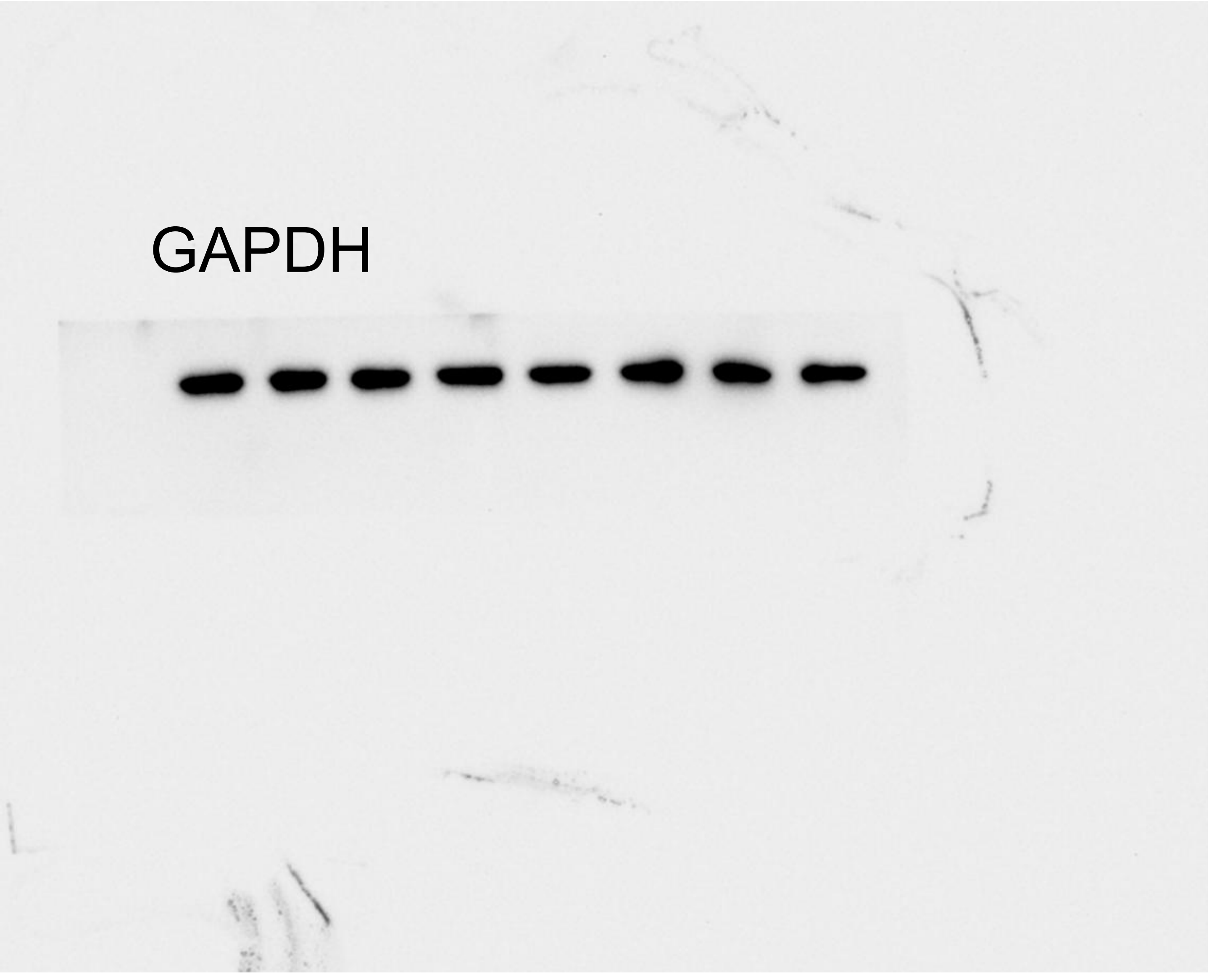

Figure S6A

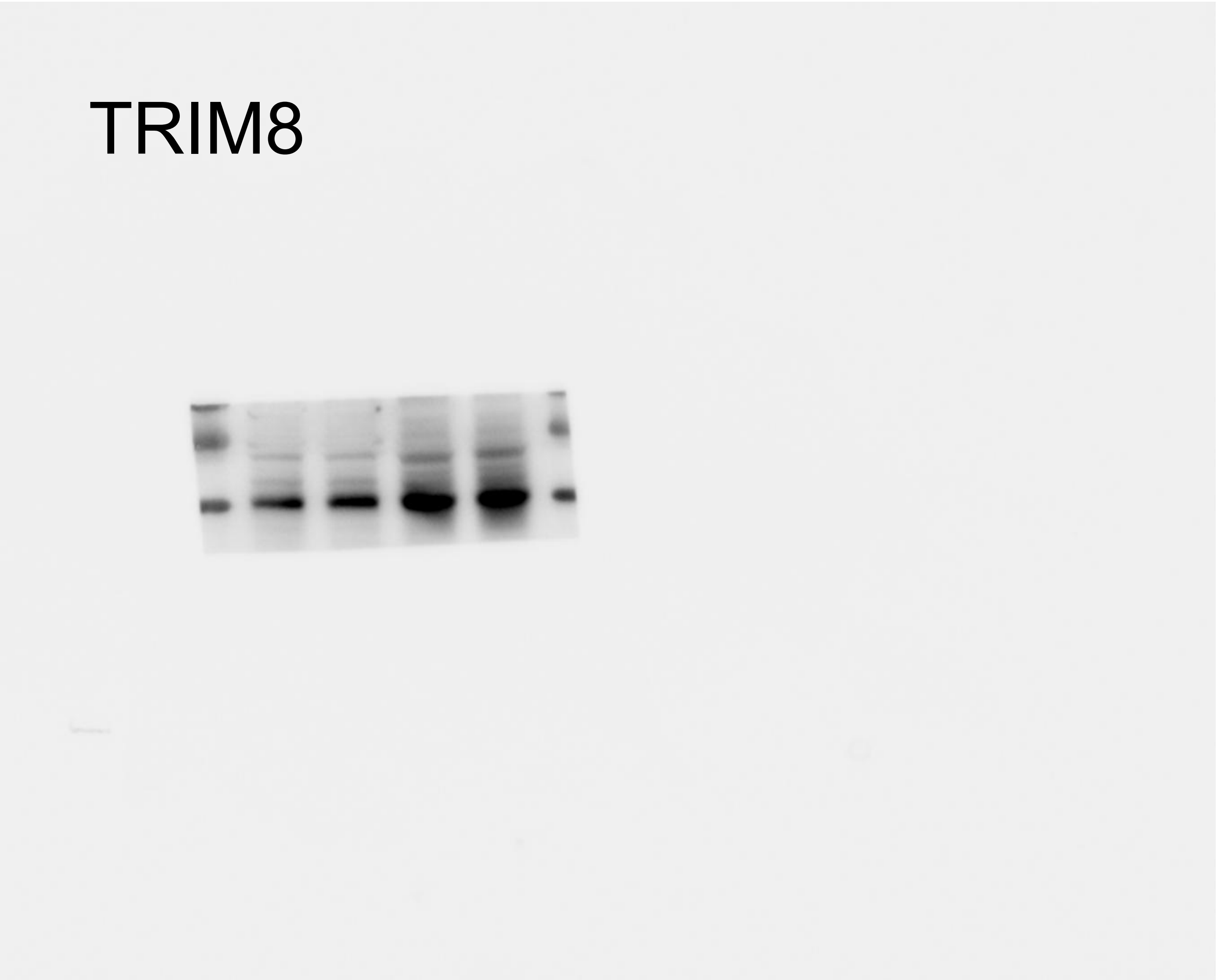

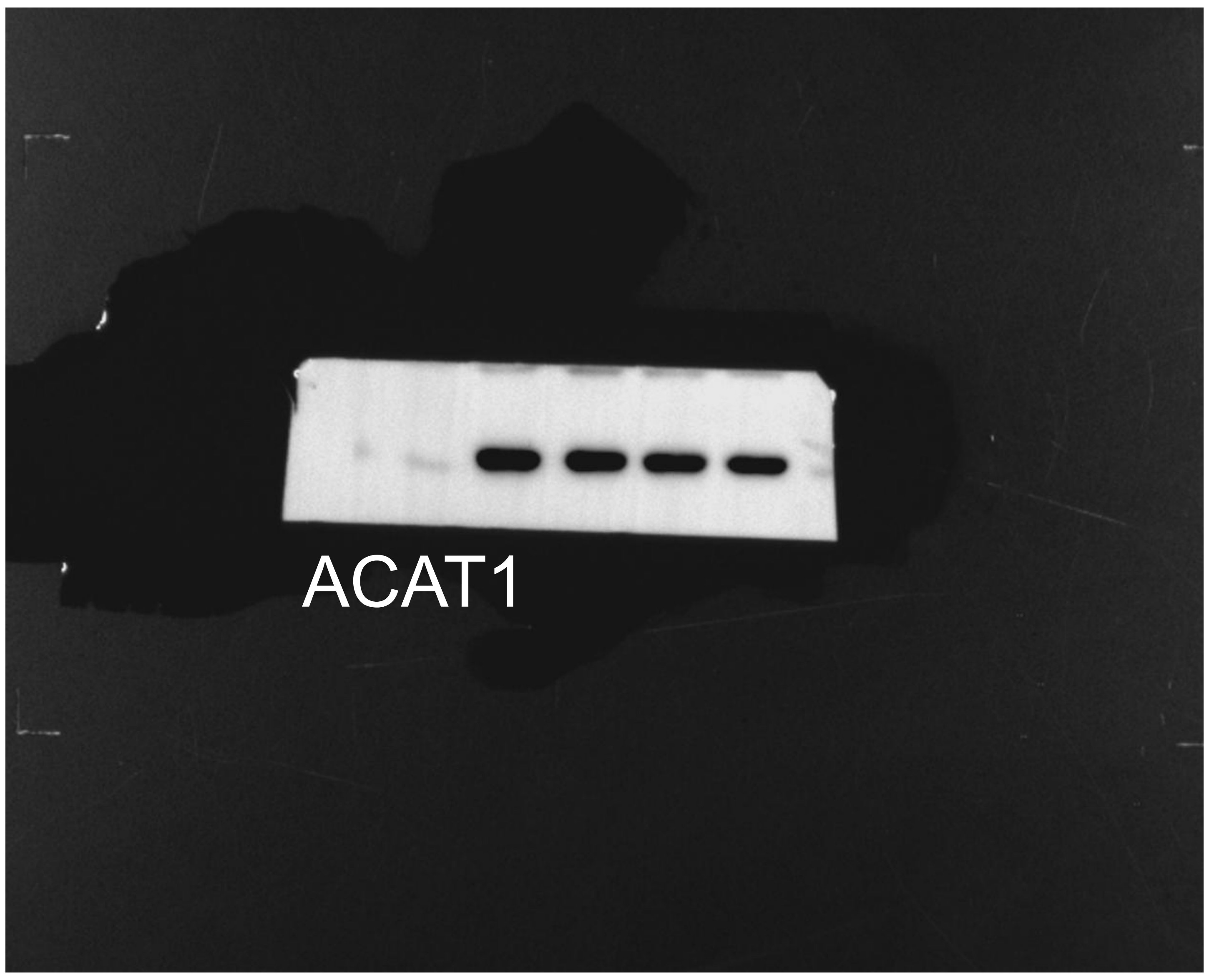

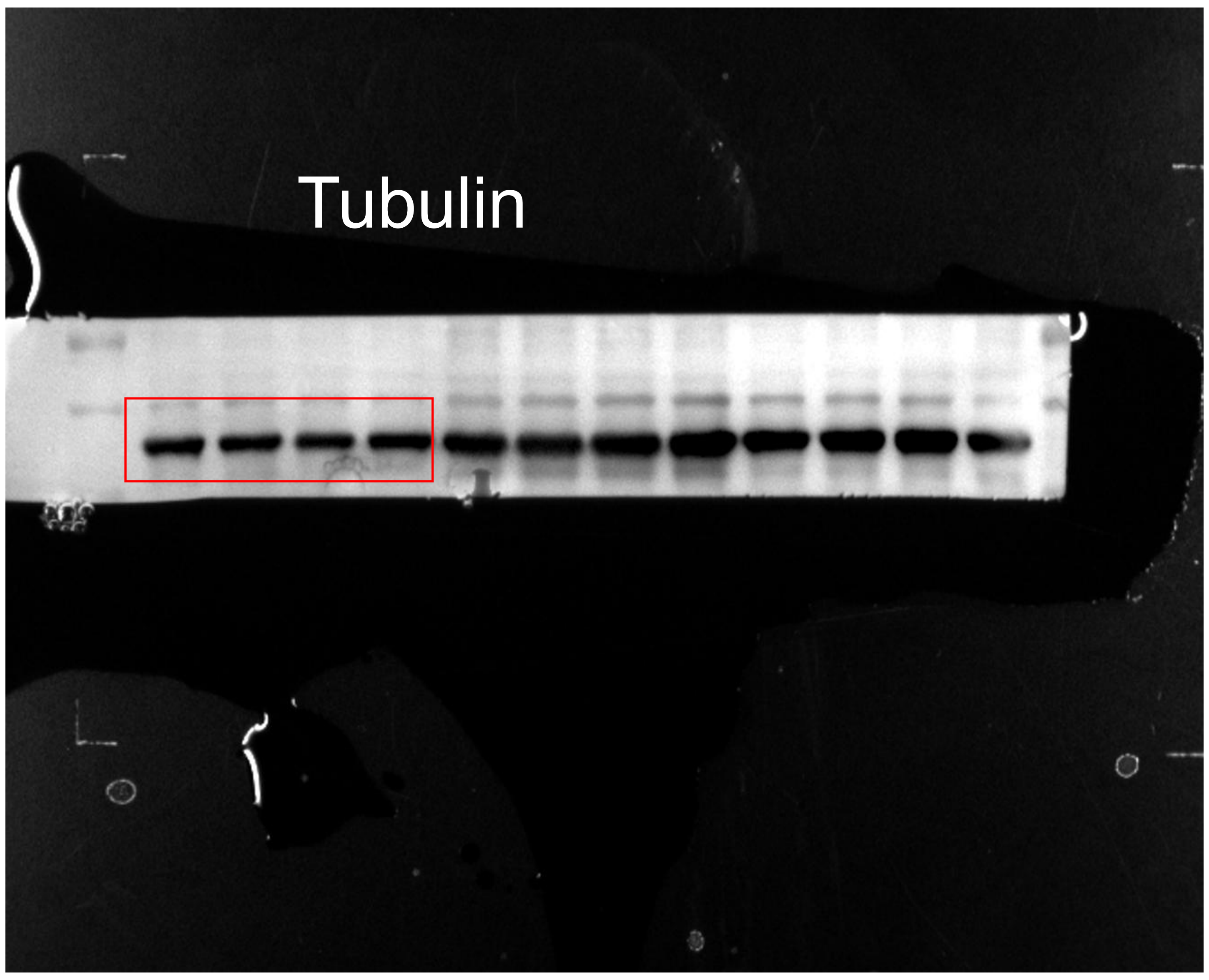

Supplement: Supplementary file 10 — Uncropped original Western blots [file 41419_2025_8015_MOESM10_ESM.pdf]
